# Comprehensive analysis of proline metabolizing genes reveals their functional diversification and abiotic stress response in *Solanum lycopersicum*

**DOI:** 10.1371/journal.pone.0335608

**Published:** 2025-10-27

**Authors:** Md. Afser Rabbi, Md. Muluk Hossain, Md. Rihan Kabir Shuvo, Asifur Rob Bhuya, Ajit Ghosh

**Affiliations:** Department of Biochemistry and Molecular Biology, Shahjalal University of Science and Technology, Sylhet, Bangladesh; South Asian University, INDIA

## Abstract

Proline plays a crucial role in plant stress responses. Proline metabolizing genes (PMGs) are a group of enzymes involved in its catabolism in mitochondria and the biosynthesis in the chloroplast and cytoplasm. A total of 21 PMGs were identified in *Solanum lycopersicum*. Among them, 2 gene pairs were identified as tandemly duplicated, and 6 gene pairs were segmentally duplicated. Phylogenetic analysis revealed distinct gene clusters, suggesting functional diversification. Gene structure analysis provided insights into the arrangement of coding and non-coding regions, while domain analysis highlighted conserved sequences for functional predictions and evolutionary conservation. Microarray expression data of the identified genes revealed that *SlOAT8* exhibited maximum expression in different anatomical tissues, particularly in ovules, and *SlOAT9* showed maximum response at developmental stages associated with shoot growth. Under stress conditions, *SlOAT8* and *SlP5CS1* were upregulated in exposure to drought stress but downregulated in response to heat and salt stress. Meanwhile, *SlOAT4* was strongly expressed only in roots during salt stress. The qRT-PCR analysis demonstrated significant upregulation of *SlOAT8*, *SlP5CDH2*, and *SlP5CR* alongside a significant downregulation of *SlP5CS1* under abiotic stress conditions. Furthermore, biochemical assay indicates the accumulation of proline and H_2_O_2_ under stressed conditions. These findings provide an extensive study on the PMGs, which will help in the development of a stress-resilient tomato plant in further.

## Introduction

Abiotic stress factors, including drought, salinity, extreme temperatures, and heavy metal toxicity, critically reduce agricultural production and create serious concerns for global food security [1,2]. To cope with these stressors, plants have evolved a variety of adaptation strategies, including the accumulation of osmoprotectants, like proline [3], trehalose, glycine betaine, and myoinositol [4]. Among them, proline is a non-essential amino acid that is the only one of the proteogenic amino acids where the α-amino group is present as a secondary amine [5]-, plays a pivotal role in plant stress responses due to its unique properties, including acting as an osmolyte [6], a metal chelator [7], an antioxidant defense molecule [8], and a signaling molecule [9]. For developing stress-resilient crops, it is essential to understand the genetic basis of proline metabolism and how it is regulated under stress conditions [10].

**Fig 1:**
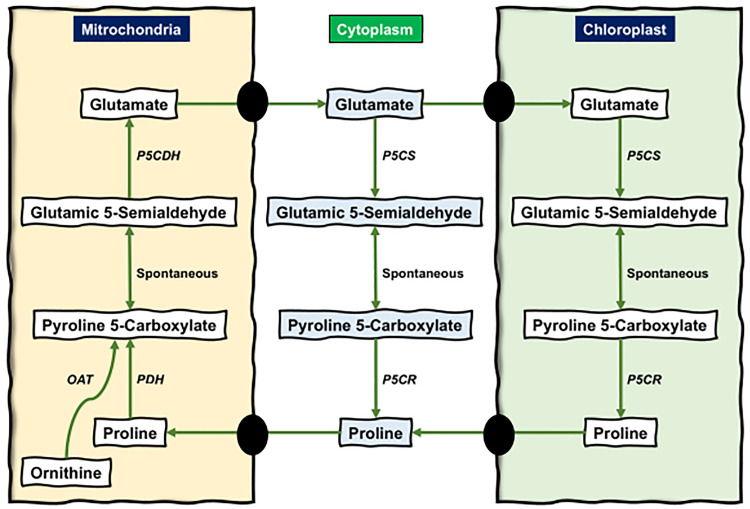
Proline anabolism and catabolism pathways. PDH, OAT, and P5CDH enzymes help in proline catabolism in mitochondria, whereas P5CS and P5CR help in the anabolism of proline in the cytoplasm and chloroplast, respectively. Proline Dehydrogenase (PDH), Pyrroline 5-Carboxylate Dehydrogenase (P5CDH), Pyrroline 5-Carboxylate Synthase (P5CS), Pyrroline 5-Carboxylate Reductase (P5CR), Ornithine Aminotransferase (OAT).

Accumulation of proline was observed in response to different stresses [11]. Proline serves as an osmoprotectant to preserve cell turgor and stabilize the structures of cells [12]. Further studies demonstrated proline’s involvement in scavenging free radicals [13], shielding proteins and membranes [14], and regulating gene expression [15]. The proline metabolism pathway involves several key enzymes that govern the biosynthesis and catabolism of proline. The central enzymes in this pathway include proline dehydrogenase (PDH), pyrroline 5-carboxylate dehydrogenase (P5CDH), pyrroline 5-carboxylate synthase (P5CS), pyrroline 5-carboxylate reductase (P5CR), and ornithine aminotransferase (OAT) [16–18]. In the cytoplasm and chloroplasts, P5CR and P5CS catalyze a two-step reduction process that converts glutamate to proline. However, proline catabolism occurs in the mitochondria and is carried out by the successive actions of P5CDH and PDH (Fig 1), ultimately yielding glutamate. Glutamate can be synthesized from ornithine. This process includes OAT, which converts ornithine to pyrroline 5-carboxylate (P5C), and then P5CDH subsequently converts P5C to glutamate (Fig 1).

Each of the proline catabolizing enzymes plays a unique and crucial role in regulating proline levels within plant cells. PDH function is suppressed by stress, which promotes proline buildup inside the cell [19]. PDH is essential for the defence against pathogens in *Arabidopsis* [20] and protection against hydrogen peroxide (H_2_O_2_) induced cell death [21]. P5CDH is normally downregulated under stressful conditions, which adds to the build-up of proline [22] and may be involved in redox balance and nitrogen absorption [23]. Proline levels rise when P5CS is markedly elevated in response to stress [24]. Distinct P5CS isoforms also play distinct functions depending on the specific stress conditions [25]. P5CR activity is also frequently elevated in response to stress, which facilitates proline accumulation. Different stressors cause various P5CR isoforms to express themselves differently [26]. Evidence suggests that OAT, in specific situations, contributes to proline biosynthesis, but its function in proline metabolism is still debatable [27]. OAT is a highly conserved enzyme found across prokaryotes, fungi, and higher plants. Although its subcellular localization varies among organisms [28]. In prokaryotes and fungi, OAT is localized in the cytosol, whereas in higher plants, it is primarily localized in the mitochondria [28]. Cytosolic localization has been experimentally confirmed in several fungi, including *Neurospora crassa* [29]*, Saccharomyces cerevisiae* [30]*,* and *Agaricus bisporus* [31,32]. Cytosolic OAT activity has been associated with stress-induced proline accumulation, which enhances tolerance to a broad range of abiotic stresses such as drought and salinity, as well as biotic challenges including pathogen attack [28,33]. OAT is found indirectly supporting photosynthetic carbon assimilation in the chloroplast by maintaining nitrogen balance in the *Oryza sativa* plant. *OsOAT is* also found to influence chloroplast-mediated photosynthesis and carbon cycling, ensure energy supply, and cell wall formation [34]. Despite various compartmentalizations, OAT is conserved across species and plays a crucial role in proline metabolism and stress adaptation.

Among the mentioned enzymes, OAT, PDH, and P5CDH are mitochondrial enzymes that catabolize proline, and P5CS and P5CR are cytoplasmic and chloroplastic enzymes which involved in anabolism [35]. The expression of these genes is regulated and varies in response to different biotic and abiotic conditions [36]. *Solanum lycopersicum*, widely known as tomato, is an important horticultural crop with significant commercial value [37]. However, despite extensive research on its stress responses, the genomic identification and expression profiling of proline in tomato are still unexplored. The study aims to identify all proline-metabolizing genes (PMGs) in *S. lycopersicum* utilizing the complete genomic analysis and to examine the expression patterns of these genes under different anatomical tissues, developmental stages, and abiotic stress conditions. Quantitative real-time PCR (qRT-PCR) was performed to validate the expression patterns of selected genes obtained from the transcriptome analysis. The stress-specific accumulation of proline and H_2_O_2_ levels was also measured in response to different abiotic stresses. This research will provide insight into the regulatory processes of proline metabolism in plants and contribute to raising crop stress tolerance.

## Methods and materials

### Identification, annotation, and characterization of PMGs in *S. lycopersicum*

Using previously known proteins from *Arabidopsis thaliana* as query sequences [36], a BLASTp search was performed against the tomato genome ‘*Solanum lycopersicum ITAG4.0*’ in the Phytozome database (https://phytozome-next.jgi.doe.gov/) to find the PMGs of *S. lycopersicum.* The InterProScan (https://www.ebi.ac.uk/interpro/search/sequence/) website was used to confirm the presence of the conserved domain of each protein sequence, namely PF00202 domain for OAT, PF00171 and PF00696 domains for P5CS, PF14748 and PF03807 domains for P5CR, and PF01619 domain for PDH. All the confirmed PMGs were named according to their order of chromosomal locations [38], denoted by the prefix “Sl” for *S. lycopersicum* [39]. The Expasy protparam tool (https://web.expasy.org/protparam/) was used to predict the physiochemical parameters of the protein, mass, and isoelectric point (pI). The chromosomal locations and coding sequence (CDS) coordinates, from 5’ to 3’, were obtained from the phytozome database (https://phytozome-next.jgi.doe.gov/). The subcellular localization of each gene involved in proline metabolism was predicted using the DeepLoc 2.0 server (https://services.healthtech.dtu.dk/services/DeepLoc-2.0/).

### Chromosomal localization and duplication analysis

Circos software [40] was used to display the genomic positions and duplication events of all identified PMGs in *S. lycopersicum*. The plant duplication database (http://chibba.agtec.uga.edu/duplication/index/downloads) provided information on the duplication of proline-metabolizing genes. Sequence similarity of the PMGs was used to predict duplication occurrences [41]. Proteins with sequence similarity of more than 90% were categorized as segmental duplications [42]. Tandem duplicated genes were identified within a 100 kb genomic region [41]. Using information from the same database, the nonsynonymous rate (Ka), synonymous rate (Ks), and evolutionary constraint (Ka/Ks) were calculated to examine these duplications in more detail. Positive, neutral, and negative selection are indicated, respectively, by Ka/Ks ratios that are larger than 1, equal to 1, or less than 1. By using the formula T = Ks/2x to the synonymous mutation rate (x = 6.56 × 10^−9^) substitutions per synonymous site per year), the divergence time of gene pairs was determined [43].

### Evolutionary relationships and phylogenetic analysis

Multiple sequence alignments of five family members of the PMGs were performed for selected plant species, including *S. lycopersicum, A. thaliana, Oryza sativa, Zea mays, Glycine max, Solanum tuberosum, Triticum aestivum, Sorghum bicolor,* and *Cucumis sativus*. These alignments were conducted using MEGA11 software with default parameters. The resulting alignments were used for evolutionary genetic analysis and for constructing a phylogenetic tree. The phylogenetic tree was generated using MEGA11 (https://www.megasoftware.net/) based on the maximum-likelihood method with a bootstrap value of 100. The tree was subsequently colored according to monocot and dicot plant classifications using iTOL (https://itol.embl.de/).

### Gene structure and domain analysis

The Gene Structure Display Server 2.0 (http://gsds.cbi.pku.edu.cn/) was used to analyze the gene structures (intron-exon) of PMGs. Using Pfam (http://www.pfam.xfam.org/) conserved domains in the proline-metabolizing proteins were identified. The TBTools [44] were used to visualize the domain architecture and gene structure.

### Expression profiling of PMGs

Microarray expression data were collected from the publicly accessible Genevestigator database [45] (https://genevestigator.com/). The normalized expression data were collected at six distinct stages of development, including main shoot growth, inflorescence visible, flowering, fruit formation, ripening, and fruit ripening complete. Additionally, expression data have been collected from different anatomical tissues such as fruit, root, seed, ovule, leaf, flower, and pollen. The mean expression levels for each anatomical and developmental stage were used to construct a heat map.

Expression data in response to different abiotic stressors, such as drought, heat, and salinity, are collected from the NCBI database, available in the GEO repository. Drought and heat stress data were retrieved under the accession number GSE151277 (https://www.ncbi.nlm.nih.gov/geo/query/acc.cgi?acc=GSE151277), while salinity stress data were accessed under the accession number GSE217631 (https://www.ncbi.nlm.nih.gov/geo/query/acc.cgi?acc=GSE217631). High-throughput sequencing data were retrieved from those accession numbers, and those data were calculated to obtain fold change values. These values were then used to create a heat map using TBtools software.

### Plant materials and stress treatment

Tomato seeds of the BARI-16 variety were grown in the controlled environmental conditions of 25 ± 2°C temperature. Fifteen days’ old seedlings were used for various abiotic stress treatments, such as salt (200 mM NaCl), drought (150 mM Mannitol), oxidative (30% H_2_O_2_), cold (4°C), and heat (42°C) [46] for 12h; and samples were collected at two time points (6h and 12h) for optimal responsiveness following previously established protocols [41,47,48]. The untreated seedlings were considered as a control for all these stresses.

### RNA extraction, cDNA synthesis, and quantitative real-time PCR analysis

Total RNA was extracted from frozen leaf tissue samples collected 6h post-treatment using the SV Total RNA Isolation System (Promega Corporation, USA), following the manufacturer’s protocol. The concentration and the purity of the extracted RNA were quantified using a Thermo Scientific NanoDrop spectrophotometer, and RNA quality was assessed via agarose gel electrophoresis. The first-strand cDNA was synthesized by taking 10 μg of RNA according to the manufacturer’s protocol of GoScript™ Reverse Transcriptase (Promega Corporation, USA) and random primers. Gene-specific primers (S1 Table) for the selected PMGs were designed using Primer–BLAST (https://ncbi.nlm.nih.gov/tools/primer-blast). Quantitative real-time PCR (qRT-PCR) was conducted using a 96-well plate format on a QUANTSTUDIO®3 REAL-TIME PCR SYSTEM (Applied Biosystems), employing SYBR Green-based qPCR reagents, following the manufacturer’s guidelines. All reactions were performed in triplicate for each experimental condition. Relative gene expression levels were calculated using the 2^−ΔΔCt^ method [39].

### Estimation of proline and H_2_O_2_ content

Proline was quantified according to the previous report [49]. 150 mg of freshly leaf tissue was homogenized with 1.5 ml of 100 mM phosphate buffer (NaH_2_PO_4_; Na_2_HPO_4_) with a pH of 7.8. Following homogenization, the mixture was centrifuged, and the supernatant was collected for proline content estimation. 50μl of the crude mix was added to a 1 ml reaction mixture consisting of 250μl of 3% sulphosalicylic acid, 250μl of acetic acid, and 500μl of 2.5% ninhydrin solution. The mixture was boiled in a water bath for 15 minutes and then cooled on ice for 5 minutes. The absorbance was measured at 520 nm, and the proline content was determined using a standard curve.

H_2_O_2_ was determined as per the previous method [50]. 150 mg of leaf samples were homogenized with 1 ml of 0.1% trichloroacetic acid (TCA). Following this, the resulting extract was centrifuged at 12000 × g for 15 minutes at 4°C. Combining 200μl of the leaf extract supernatant with 300μl of 100 mM potassium phosphate buffer (KH_2_PO_4_, K_2_HPO_4_) at pH 7.8, and then 500μl of a reagent containing 1 M KI was added. The blank control comprised 200μl 0.1% TCA, 300μl of 100 mM potassium phosphate buffer at pH 7.8, and 500μl of the reagent 1M KI. After allowing the reaction to proceed in the dark for 1 hour, the absorbance at 390 nm was measured. The H_2_O_2_ content is determined by using a standard curve.

## Results

### Genome-wide analysis of *S. lycopersicum* identifies twenty-one PMGs

A BLAST search run in the phytozome database revealed the presence of 21 PMGs in *S. lycopersicum*. All candidates that have been discovered display conserved domains that are typical of enzymes involved in proline metabolism, as validated by InterProScan (https://www.ebi.ac.uk/interpro/search/sequence/). The PMGs that were identified were renamed according to the locations on the chromosomes. The bioinformatic study showed that these 21 PMGs exhibit variations in their amino acid composition, with a range of 276–853 residues (Table 1). Most of these PMGs are predicted to be localized in the cytoplasm (Cyto) and mitochondria (Mito), although some are also present in plastids and the nucleus. The proteins have theoretical isoelectric points (pI) ranging from 5.58 to 9.08, and their molecular weights range from 41.32 kDa to 95.02 kDa (Table 1).

**Table 1:** Detailed information of identified PMG family members, including their subcellular localizations and amino acid numbers.

Sl no	Gene Name	Chromosome	Transcript ID	CDS coordinate (5’ to 3’)	CDS (bp)	Amino Acids	Mass (kDa)	pI	Localization
1	*SlOAT1*	4	Solyc04g054310.3.1	51582766..51588297	1422	473	51.71	8.72	Mitochondria
2	*SlOAT2*	4	Solyc04g009200.3.1	2730442..2734386	1449	482	51.50	6.53	Plastid
3	*SlOAT3*	7	Solyc07g043310.3.1	56836347..56843373	1548	515	56.71	7.64	Mitochondria
4	*SlOAT4*	7	Solyc07g006810.3.1	1620714..1631343	2562	853	95.02	5.87	Mitochondria
5	*SlOAT5*	8	Solyc08g048450.4.1	12385627..12397056	1143	380	41.46	6.17	Cytoplasm
6	*SlOAT6*	8	Solyc08g080370.3.1	61781981..61785820	1383	460	48.99	5.96	Plastid
7	*SlOAT7*	8	Solyc08g014610.4.1	4752898..4762821	1377	458	50.54	6.15	Cytoplasm
8	*SlOAT8*	10	Solyc10g076250.2.1	58238533..58242662	1437	478	52.22	6.94	Mitochondria
9	*SlOAT9*	12	Solyc12g006470.2.1	963073..969168	1377	458	50.64	6.43	Cytoplasm
10	*SlOAT10*	12	Solyc12g006450.2.1	928832..934720	1551	516	56.85	6.55	Mitochondria
11	*SlP5CS1*	6	Solyc06g019170.3.1	16130821..16139871	2154	717	77.48	5.58	Cytoplasm
12	*SlP5CS2*	8	Solyc08g043170.4.1	22604683..22617550	2250	749	81.47	6.14	Cytoplasm
13	*SlP5CR*	2	Solyc02g068640.3.1	36609470..36616912	831	276	28.60	8.57	Cytoplasm
14	*SlPDH1*	2	Solyc02g089620.3.1	49418488..49420765	1491	496	55.81	6.21	Mitochondria
15	*SlPDH2*	2	Solyc02g089630.3.1	49421846..49424545	1485	494	55.68	8.65	Mitochondria
16	*SlP5CDH1*	3	Solyc03g114360.4.1	58790585..58793647	1128	375	41.32	8.61	Nucleus, Cytoplasm
17	*SlP5CDH2*	5	Solyc05g005290.4.1	277894..279752	930	309	35.22	9.08	Nucleus, Cytoplasm
18	*SlP5CDH3*	5	Solyc05g005280.4.1	273340..276208	948	315	35.47	6.6	Nucleus, Cytoplasm
19	*SlP5CDH4*	6	Solyc06g066330.3.1	39251326..39256766	1785	594	67.31	7.54	Nucleus
20	*SlP5CDH5*	8	Solyc08g005270.3.1	206049..215501	1803	600	67.86	6.39	Nucleus
21	*SlP5CDH6*	8	Solyc08g076420.3.1	58541206..58550166	1794	597	67.31	7.2	Nucleus

### Chromosomal localization, gene duplication, and Ka/Ks calculation

**Fig 2:**
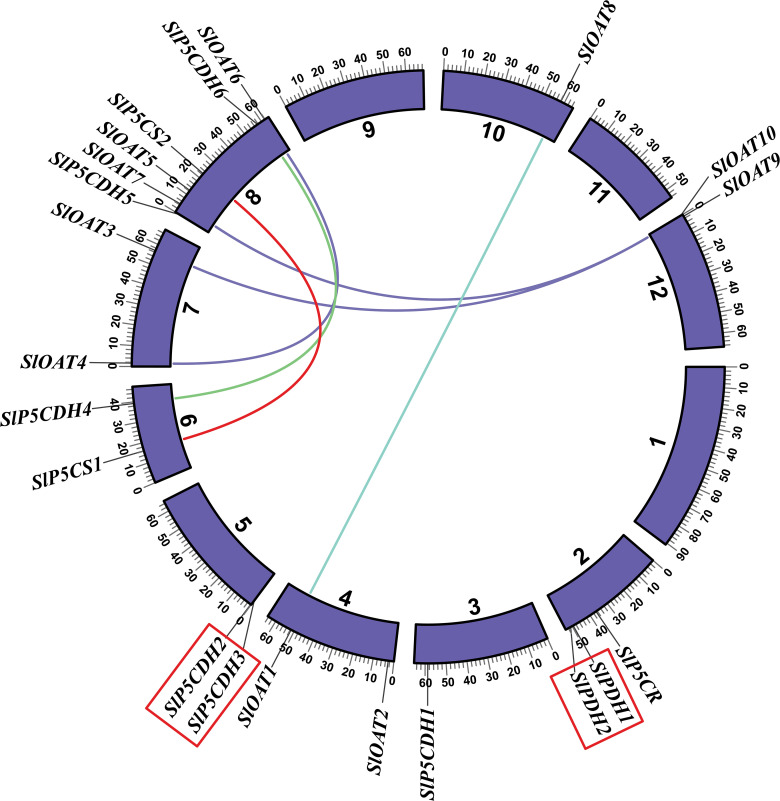
Chromosomal distribution of proline-metabolizing genes. All the identified 21 proline-metabolizing genes were indicated on 12 different chromosomes of *Solanum Lycopersicum*. Chromosomes were depicted with different color bars generated by TBTools software. Tandem duplication events were indicated by a red box outside the gene names, and the colorful lines between different chromosomes indicate segmental duplication. The figure was created using Circos software (http://circos.ca/).

The chromosomal distribution of PMGs in *S. lycopersicum* reveals that these genes are unevenly distributed across 9 out of the 12 chromosomes (Fig 2). Chromosome 8 contains the highest number of PMGs, with a total of 6 genes. Chromosomes 4, 5, 6, 7, and 12 contain two genes each, while chromosome 3 has only one gene. Notably, chromosomes 1, 9, and 11 don’t contain any genes. Both tandem duplication and segmental duplication events have been seen among PMGs.

Tandem duplication gene pairs include *SlPDH*1/*SlPDH*2 and *SlP5CDH*2/*SlP5CDH*3 (Fig 2). Segmental genome duplication pairs identified are *SlP5CS*1/*SlP5CS*2, *SlP5CDH*4/*SlP5CDH*6, *SlOAT*1/*SlOAT*8, *SlOAT*4/*SlOAT*6, *SlOAT*7/*SlOAT*9, and *SlOAT*3/*SlOAT*10. All the duplication pairs are under negative purifying selection pressure as the Ka/Ks values of the pairs are less than 1 (Table 2). Among the duplication events, the one between *SlOAT*7 and *SlOAT*9 is comparatively recent and occurred approximately 20.53 million years ago (Mya). In comparison, the duplication event between *SlOAT*4 and *SlOAT*6 is the most ancient and occurred around 169.92 Mya.

**Table 2:** Gene duplication analysis of PMGs.

Sl no.	Locus_1	Locus_2	Ka	Ks	Ka/Ks	Selection Pressure	Time (MYA)	Duplication type
1	*SlPDH1*	*SlPDH2*	0.149	0.852	0.176	Purifying Selection	64.99	Tandem
2	*SlP5CS1*	*SlP5CS2*	0.082	0.525	0.158	Purifying Selection	40.03	Segmental
3	*SlP5CDH4*	*SlP5CDH6*	0.301	0.955	0.315	Purifying Selection	72.84	Segmental
4	*SlP5CDH2*	*SlP5CDH3*	0.145	0.341	0.426	Purifying Selection	26.01	Tandem
5	*SlOAT1*	*SlOAT8*	0.292	1.821	0.161	Purifying Selection	138.84	Segmental
6	*SlOAT4*	*SlOAT6*	0.865	2.229	0.388	Purifying Selection	169.92	Segmental
7	*SlOAT7*	*SlOAT9*	0.079	0.269	0.296	Purifying Selection	20.53	Segmental
8	*SlOAT3*	*SlOAT10*	0.110	0.748	0.147	Purifying Selection	57.05	Segmental

### Exon-intron organization and functional domain analysis of PMGs

**Fig 3:**
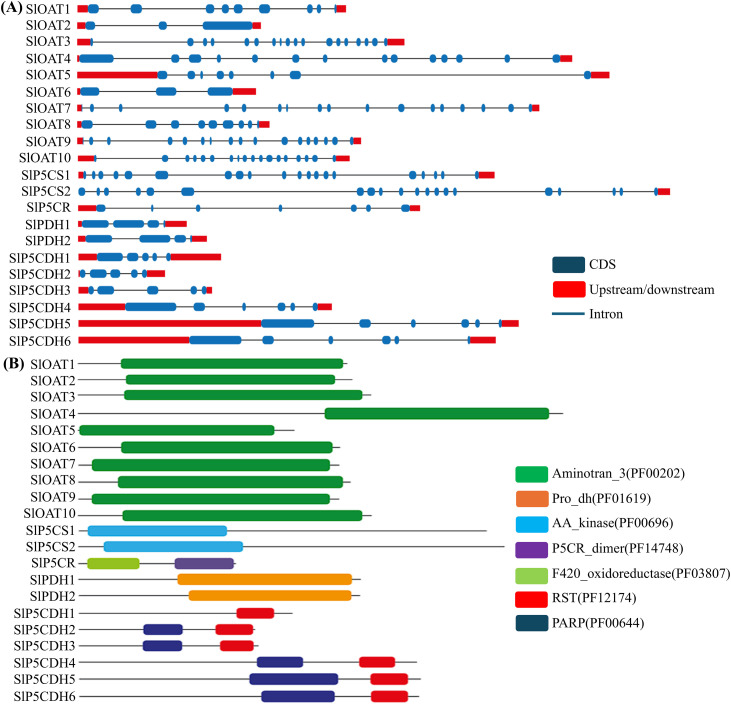
Gene structure and presence of functional domains in proline metabolizing members. **(A)** Exon-intron analyses of proline-metabolizing genes were created with the GSDS tool. Lengths of introns and exons of PMG genes were exhibited proportionally. Exons are shown as blue boxes, and introns are shown as black lines, while upstream/downstream regions are shown as red boxes. **(B)** The conserved domain structure of 21 proline-metabolizing genes, represented in the colored boxes, was identified using protein sequences with the NCBI batch web Conserved Domains Search tool. The green box represents the Aminotran_3 domain, the yellow box represents the Pro_dh domain, the blue box represents the AA_kinase domain, the indigo box represents the P5CR_dimer domain, the light green box represents the F420_oxidoreductase domain, the red color represents the RST domain, and the blue color represents the PARP domain.

Through an analysis of the coding DNA sequences of each gene involved in proline metabolism alongside their corresponding genomic DNA sequences, the Gene Structure Display Server 2.0 (http://gsds.cbi.pku.edu.cn/) visually illustrated the transcript structure of each PMG. Evaluation of 21 distinct proline-metabolizing transcripts uncovered notable variations in their gene compositions (Fig 3A). These PMGs differed in size and featured a range of introns, ranging from two to eight. Certain genes like *SlPDH1, SlPDH2*, and *SlOAT6* had just 3 exons, while others possessed 3, 6, or 8 exons (Fig 3A). Patterns in the number and positioning of exons and introns were shared across different exon-intron distributions. Despite similarities within groups, unique distinctions among individuals were also identified.

Domain analysis showed that SlP5CR and all the SlP5CDH members have 2 functional domains (Fig 3B). All the SlOAT, SlP5CS, SlPDH gene members and SlP5CDH1 have a single domain. SlOAT contains an Aminotran_3(PF00202) domain that transfers an amino group from acetylornithine to alpha-keto-glutarate. SlP5CS contains an AA_kinase (PF00696) domain that moves a phosphate group onto proteins by a process known as phosphorylation. SlPDH contains Pro_dh (PF01619) domain. Furthermore, SlP5CR protein contains 2 functional domains, i.e., F420_oxidoreductase (PF03807) and P5CR_dimer (PF14748) and SlP5CDH contain PARP(PF00644) and RST(PF12174) domains (Fig 3B).

### Phylogenetic analysis of PMGs

**Fig 4:**
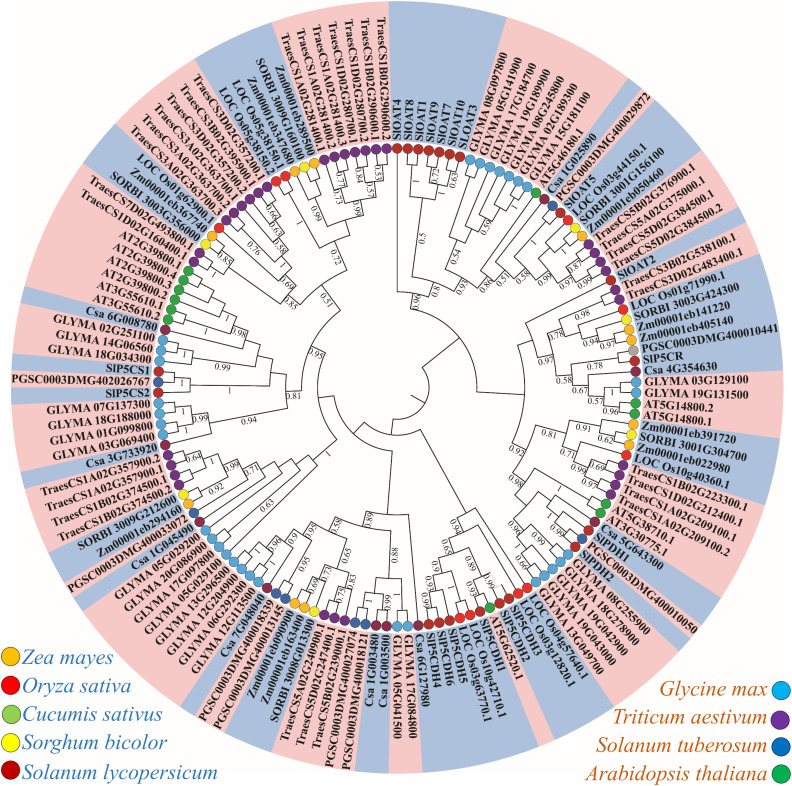
Phylogenetic analysis of proline metabolizing members. Proline metabolizing proteins from various species, including *Arabidopsis thaliana, Glycine max, Solanum tuberosum, Cucumis sativus, Solanum lycopersicum, Oryza sativa, Zea mays, Sorghum bicolor,* and *Triticum aestivum,* were collected from various databases. A total of 143 protein sequences from 9 different species were aligned by ClustalW, followed by the construction of a maximum-likelihood tree using MEGA11 with 100 bootstrap replicates. Bootstrap values greater than 0.5 were shown in the different branching points of the tree, indicating significant clustering. In the tree, the light blue color represents dicots the orange color represents monocots, and the smaller circular color denotes distinct plant species.

To explore the evolutionary relationships and genetic similarities among gene families responsible for proline metabolism, a phylogenetic analysis was conducted using 143 protein sequences (S1 Dataset) from both monocot plants, including *S. lycopersicum, O. sativa, Zea mays, Sorghum bicolor,* and *Triticum aestivum,* and dicot plants, including *A. thaliana, Glycine max, Solanum tuberosum,* and *Cucumis sativus*. Using the proline metabolizing protein sequences from these species, a maximum likelihood phylogenetic tree was constructed (Fig 4). Remarkably, several common clades were observed among PMGs from various species, with these genes distributed throughout the entire phylogenetic tree. To distinguish between monocot and dicot plants, a color scheme was employed in the phylogenetic tree where light blue represented dicots and orange represented monocots. This graphical visualization illustrates the evolutionary relationships and unique features of PMGs among the species. The analysis indicates that PMGs of *S. lycopersicum* are phylogenetically like those of monocot plants. Observation further reveals that most *SlOAT* proteins group together within the same families. Notably, *SlOAT2* and *SlP5CR* exhibit the greatest phylogenetic distance from the other PMGs of *S. lycopersicum.* Moreover, specific families, including *SlP5CDH*, *SlPDH*, and *SlP5CS*, form distinct subclusters within their respective members. Additionally, PMGs from various plant species are observed to form smaller clusters within the phylogenetic tree.

### Variation of transcript abundance across different developmental stages and anatomical tissues

**Fig 5:**
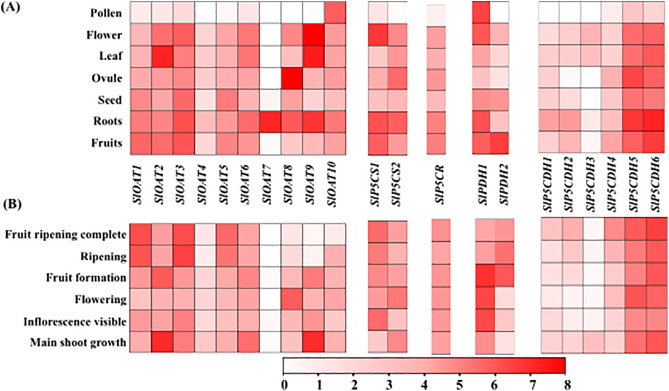
Expression profiling of proline-metabolizing transcripts in different developmental stages and anatomical tissues. **(A)** Several anatomical tissues were used to evaluate the expression of PMGs. A heat map showing the PMGs distinct patterns of expression. **(B)** Several developmental stages were used to evaluate the expression of PMGs as heat map. Where white indicates the least expression and red represents the most expression, the intensity of the colour is directly proportional to the relative expression levels. TbTool is used to produce the heatmap.

Unraveling the functions of genes related to proline metabolism involved examining their expression levels throughout six distinct developmental phases and seven different anatomical tissues (Fig 5). The data revealed that 21 PMGs displayed varying patterns of expression throughout all stages of development and in different anatomical tissues. The study utilized microarray data from genevestigator to examine the expression profiles of these genes. Within anatomical tissues, the focus was on assessing expression levels in fruits, roots, seeds, ovules, leaves, flowers, and pollen. Notably, certain genes exhibited varying degrees of expression, with *SlOAT9, SlP5CDH6, SlP5CDH5*, and *SlPDH1* showing the highest levels of expression, while *SlOAT7* exhibited the lowest (Fig 5A). This study also analyzed the expression profiles during six developmental stages of *S. lycopersicum*: main shoot growth, inflorescence visibility, flowering, fruit formation, ripening, and complete fruit ripening (Fig 5B). It was observed that several PMGs showcased distinct patterns of expression specific to each stage of development. Among these, *SlOAT2, SlOAT9*, and *SlP5CDH6* displayed the highest levels of expression, whereas *SlOAT7* and *SlP5CDH3* consistently exhibited the lowest levels of expression (Fig 5B). Conversely, the *SlP5CDH5* and *SlP5CDH6* genes show high expression levels across various anatomical tissues and developmental stages.

### Expression profiling under different abiotic stresses

**Fig 6:**
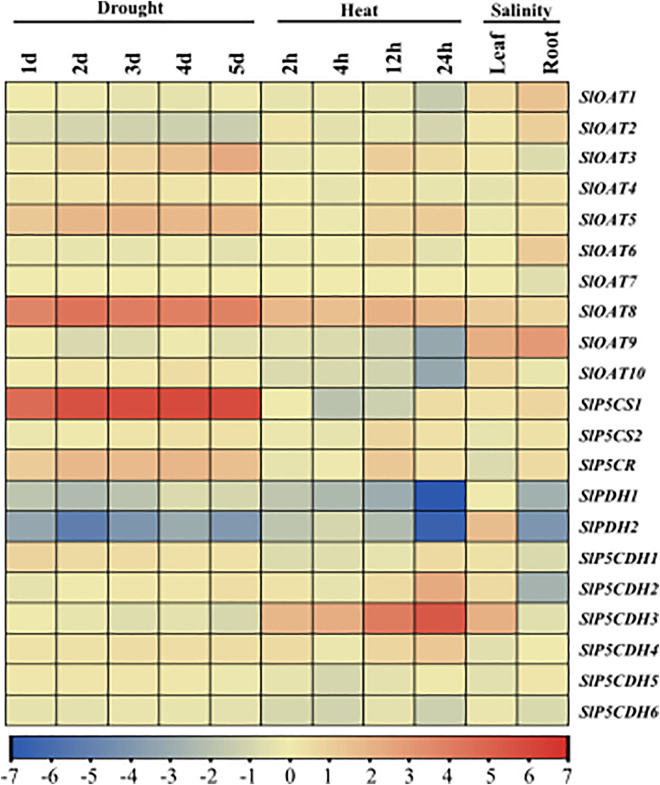
Expression analyses of proline-metabolizing genes in response to various abiotic stresses. Relative expression data of all the available transcripts were retrieved from NCBI GEO datasets. The datasets for drought (5-time points) and heat stress (4-time points) were obtained under accession number GSE151277 (https://www.ncbi.nlm.nih.gov/geo/query/acc.cgi?acc=GSE151277), and salinity (at leaf and root level) stress data was retrieved from GSE217631 (https://www.ncbi.nlm.nih.gov/geo/query/acc.cgi?acc=GSE217631). The red color represents the highest expression level, and the blue color represents the lowest expression levels of the genes in response to abiotic stresses. Tbtools were used to construct the heatmaps, indicating up-regulation indicated by red color and downregulation indicated by the deep blue color of the corresponding transcripts.

To understand the role of PMGs in stress regulation, the expression of all the identified transcripts for *SlOAT, SlP5CS, SlP5CR, SlPDH*, and *SlP5CDH* was investigated under a variety of abiotic stressors, including drought, heat, and salinity, at different time points and tissues. Different transcripts are expressed differently under different stresses. Under drought stress conditions, the expression of *SlOAT8* and *SlP5CS1* genes was upregulated consistently from 1d to 5d of stress exposure. However, these genes were downregulated in response to heat and salt stress (Fig 6). Conversely, *SlPDH1* and *SlPDH2* were consistently downregulated in response to all stressors, particularly after 24h of heat exposure. Notably, *SlOAT4* expression was observed exclusively under salt stress at the root level. *SlP5CDH3* expression was detected only during heat stress, specifically after 12h and 24h of exposure (Fig 6). In contrast, *SlOAT9* and *SlOAT10* were downregulated in 24h heat stress exposure. Overall, most transcripts were slightly downregulated in response to heat, salt, and drought stressors.

### Validation of selected genes under various abiotic stresses

**Fig 7:**
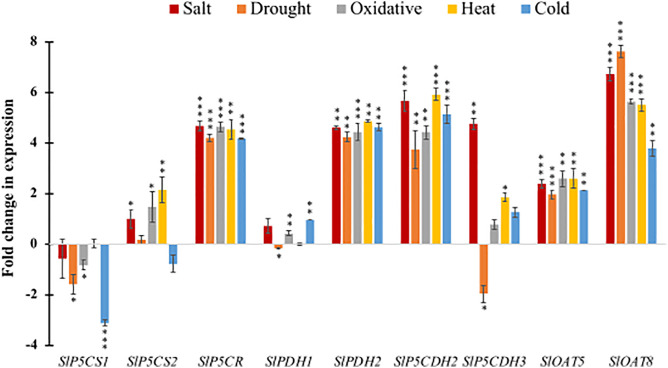
Abiotic stress responsiveness of proline-metabolizing genes in tomato by qRT-PCR. The expression pattern of 9 selected genes, including *SlP5CS1, SlP5CS2, SlP5CR, SlPDH1, SlPDH2, SlP5CDH2, SlP5CDH3, SlOAT5,* and *SlOAT8,* was investigated by exposing them to salinity, drought, oxidative, heat, and cold stress. Different colors in the bar diagram correspond to different stresses, and the average fold change in expression was calculated against the respective control. The significance level of the paired t-test is denoted by *, **, and *** with a p-value < 0.05, < 0.01, and <0.001, respectively.

To validate the abiotic stress-responsiveness of PMGs in tomato, qRT-PCR was conducted under various stress conditions, including salinity, drought, oxidative, heat, and cold stresses. Nine genes were selected for expression profiling under different abiotic stresses (Fig 7). These selected genes include *SlP5CS1, SlP5CS2, SlP5CR, SlPDH1, SlPDH2, SlP5CDH2, SlP5CDH3, SlOAT5,* and *SlOAT8.* Among them, *SlP5CS1* generally exhibits downregulation under stress conditions, particularly cold stress, while *SlP5CS2* is notably upregulated by oxidative and heat stress, with moderate induction by salt and drought (Fig 7), promoting proline accumulation, cell survival, and recovery after stresses. *SlP5CR* consistently demonstrates strong upregulation across all five stress treatments, suggesting a significant role in the plant’s stress response. For the *SlPDH* genes, *SlPDH1* shows modest upregulation under salt and cold stress, whereas *SlPDH2* is significantly upregulated by salt, drought, oxidative, and heat stress, and moderately by cold stress (Fig 7). Among the *SlP5CDH* genes, *SlPCDH2* displays significant upregulation under salt, heat, and cold stress, alongside moderate upregulation by drought and oxidative stress. In contrast, *SlP5CD3* is distinctly downregulated by drought yet upregulated by salt, oxidative, and heat stress (Fig 7). Both *SlOAT5* and *SlOAT8* are upregulated by all abiotic stresses, with *SlOAT8* showing the highest induction, especially under salt, drought, and heat conditions, indicating its critical involvement in these specific stress pathways, detoxification of Reactive Oxygen Species (ROS), and osmotic adjustment via proline accumulation.

### Quantification of proline and H_2_O_2_ contents in tomato under abiotic stress conditions

**Fig 8:**
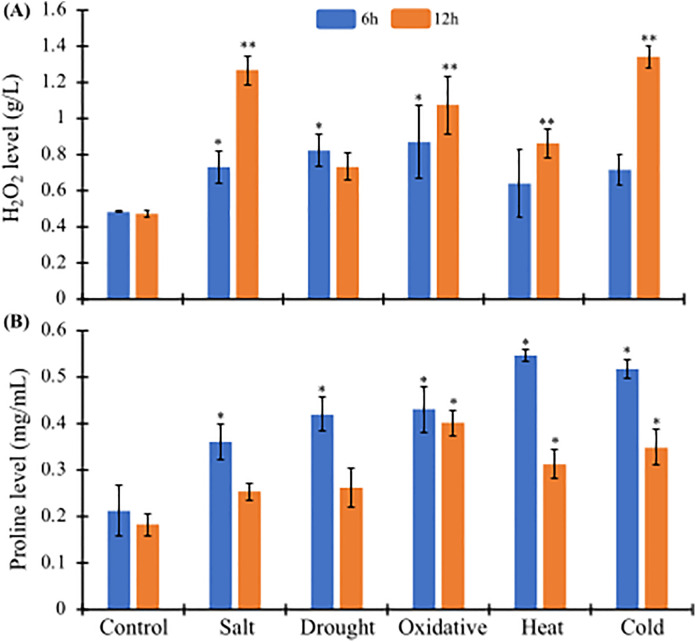
Measurement of H_2_O_2_ and proline content in different abiotic stresses. Levels of H_2_O_2_ (g/l) and proline (mg/ml) were measured in response to various abiotic stresses, such as salt, drought, oxidative, heat, and cold stresses at two different time points of stress exposure (6h and 12h). All the tests were conducted in duplicate, and the results are represented as the mean ± standard deviation (n = 3). Each bar is distinctly colored based on the time of stress exposure. The significance level of the paired t-test is denoted by *, **, and *** with a p-value < 0.05, < 0.01, and <0.001, respectively.

To gain a deeper understanding of abiotic stress adaptation in tomatoes, we measured proline and H_2_O_2_ contents under various stress conditions, including salinity, heat, cold, H_2_O_2,_ and drought, and compared them with untreated control conditions. These measurements were taken at two different time points, 6 hours and 12 hours, with proline and H_2_O_2_ contents expressed as mg/ml and g/l, respectively. H_2_O_2_ contents were significantly induced, showing a 0.5-to-3.0-fold increase compared to the control across all stress conditions. Under heat and H_2_O_2_ stress, H_2_O_2_ levels were slightly higher at 12 hours compared to 6 hours of stress exposure (Fig 8A). In contrast, cold and NaCl stress resulted in a doubling of H_2_O_2_ content after 12 hours compared to 6 hours. Conversely, under drought (mannitol) stress, H_2_O_2_ levels slightly decreased after 12 hours compared to 6 hours of exposure (Fig 8A). Similarly, proline accumulation was significantly induced by 0.5 to 2.5 times compared to the control across all stress conditions (Fig 8B). However, under heat, cold, NaCl, and drought (mannitol) stress conditions, proline levels were markedly reduced from 6 to 12 hours of exposure. In contrast, H_2_O_2_ stress led to a slight reduction in proline contents at 12 hours compared to 6 hours of exposure (Fig 8B).

## Discussion

Accumulation of proline has been seen in numerous plant species under various stress conditions, including salinity, drought, heat, and heavy metals [51–53], by increasing the rate of synthesis and decreasing the degradation rates [54]. Proline metabolism is compartmentalized throughout the entire cytoplasm, chloroplasts, and mitochondria (Fig 1). Proline plays an important role in the stabilization of subcellular structures, scavenging free radicals, and buffering cellular redox potential. In addition, it chelates heavy metals, modulates cellular functions, and triggers gene expression [55]. Proline also possesses chemical properties, including high solubility and zwitterionic structure, that are characteristic of protective and compatible solutes [56].

This study presents a comprehensive genome-wide analysis of PMGs in *S. lycopersicum*, revealing 21 distinct genes involved in proline metabolism. Previous studies had identified PMGs in different plant species, including *A. thaliana* (13 genes) and *O. sativa* (5 genes) [36]. Most plants tend to duplicate their genes to adapt to different adverse conditions of the environment during developmental stages [57]. Recent duplication events were shown between *SlOAT7* and *SlOAT9,* and more ancient events were shown between *SlOAT4* and *SlOAT6* (Fig 2). In plants, these duplication events have led to gene gain or loss and abiotic stress regulation [58]. The phylogenetic analysis provides further insights into the evolutionary relationship and genetic similarities among PMGs from both monocot and dicot plant species, including *Arabidopsis* and *O. sativa*. The constructed maximum likelihood phylogenetic tree revealed several common clades among PMGs from various plant species (Fig 4), denoting the evolutionary conservation and divergence of these genes.

The expression analysis of PMGs across developmental stages and anatomical tissues showed their vital roles in tomato plant adaptation. Microarray data revealed variable expression patterns, with genes such as *SlOAT9, SlP5CDH6, SlP5CDH5*, and *SlPDH1* showing high expression in various tissues, while *SlOAT7* exhibits low expression (Fig 5A), suggesting their contribution to fundamental metabolic processes. During developmental stages, genes like *SlOAT2, SlOAT9*, and *SlP5CDH6* were highly expressed, highlighting their importance in these processes (Fig 5B) highlight their involvement in growth related regulation. Under abiotic stress, *SlOAT8* and *SlP5CS1* were upregulated during drought but downregulated under heat and salt stress. Indicates their correlation with increased proline content (Fig 6). These stress-specific strategies suggest that drought primarily enhances proline synthesis via ornithine and glutamate-dependent pathways, whereas heat and salinity activate alternative protective mechanisms. The significant upregulation of proline biosynthetic genes, particularly *P5CS*, has been previously linked to enhanced cold stress tolerance [59].

Similarly, *P5CS* overexpression in petunia has been shown to improve cold stress resilience [60]. Conversely, *SlPDH1* and *SlPDH2* were consistently downregulated across all stressors, particularly after 24 hours of heat exposure (Fig 6), likely contributing to reduced proline degradation to maximize intercellular retention under prolonged heat stress. Expression of *SlOAT4* only under salt stress in roots and *SlP5CDH3* induction by heat stress further emphasize the distinct roles of these genes in stress responses. Moreover, OAT overexpression has been demonstrated to confer tolerance to drought, salinity, and heat stress [61]. Another key gene in the proline biosynthesis pathway, P5CR [62], was slightly upregulated under multiple abiotic stress conditions, supporting its role in proline accumulation. Overall, most transcripts showed slight downregulation in response to heat, salt, and drought stresses. The coordination of proline biosynthetic genes like P5CR and the suppression of proline-degrading genes like PDH is critical for proline accumulation and stress tolerance [63].

The differential expression patterns of *SlP5CS1, SlP5CS2, SlP5CR, SlPDH1, SlPDH2, SlP5CDH2, SlP5CDH3, SlOAT5,* and *SlOAT8* genes in *S. lycopersicum*, as observed through RNA-seq data (Fig 6) and validated by the qRT-PCR analysis (Fig 7), highlighted their integral roles in modulating abiotic stresses. There are some differences observed in the two expression profiling data, which might be due to the variations in plant growth conditions, age of the plant, stress intensity, stress exposure time, and plant material extraction protocol. Gene-specific expression patterns were evident, reflecting a tightly regulated and stress-specific transcriptional network. For instance, *SlOAT8* and *SlP5CS1* were upregulated under drought stress but downregulated under heat and salt stress (Fig 7), indicating their context-dependent roles. The upregulation of *SlOAT8* under drought highlights its potential role in proline accumulation via the ornithine pathway, whereas its downregulation under heat and salt suggests that these stresses may favor the glutamate-dependent route for proline biosynthesis. In *Arabidopsis*, *P5CS1* regulation acts as a ‘memory hub’ for stress response in the presence of light [64] and a rate-limiting enzyme in the biosynthesis of proline [65]. *SlP5CS2* expression was markedly induced under salt, drought, and oxidative stress conditions, with the strong upregulation observed during heat stress, whereas its expression was downregulated under cold stress (Fig 7), reflecting its role in proline biosynthesis and stress adaptation. But it was observed that, in the plant *Saccharum officinarum L.*, under drought treatment, *SoP5CS1* exhibited higher transcript levels compared to *SoP5CS2*, with a significant increase observed at 12 hours. These results suggest that *SoP5CS1* plays a more prominent role than *SoP5CS2* in proline biosynthesis during drought stress [66]. In contrast, *SlP5CR* showed strong and consistent induction across all stress conditions (Fig 7), suggesting a central role in stress adaptation. Since *SlP5CR* catalyzes the final step of proline biosynthesis, its ubiquitous induction likely ensures continuous proline accumulation, making it a vital node in proline metabolism. The selective expression of isoforms further illustrates the complexity and redundancy built into the proline metabolic pathway, ensuring adaptability under diverse environmental changes. *SlPDH1* and *SlPDH2*, both involved in proline catabolism, were predominantly downregulated, particularly under heat stress, indicating a deliberate suppression of proline degradation. This suggests a protective mechanism to maximize intracellular proline retention, thereby enhancing osmoprotectant, ROS scavenging, and redox buffering. Conversely, *SlP5CDH2* was significantly upregulated under salt, heat, and cold stress (Fig 7), indicating functional divergence within the gene family. The selective upregulation of *SlP5CDH2* may reflect its role in modulating proline turnover under specific stress conditions, potentially balancing proline levels with other metabolic demands. These findings align with patterns reported in *Arabidopsis thaliana* and *Oryza sativa* [36], where proline biosynthetic genes (*AtP5CS1, AtP5CS2, OsP5CS1*) are generally upregulated and catabolic genes are repressed under abiotic stress. In summary, these findings elucidate the differential expression and regulatory mechanisms of PMGs in *S. lycopersicum*, contributing to our understanding of their roles in abiotic stress responses.

This study also provides valuable insights into the biochemical stress conditions of tomatoes under various stress conditions (Fig 8). Both proline and H_2_O_2_ levels were significantly elevated across all stress conditions compared to the control, highlighting their crucial roles in stress adaptation. The accumulation of H₂O₂ across all stress conditions indicates the generation of oxidative stress as a common response (Fig 8A). Notably, H₂O₂ levels increased most markedly under cold and NaCl stress after 24 hours (Fig 8A), suggesting that these stresses strongly disrupt redox homeostasis. Elevated H₂O₂ serves both as a stress signal and as a damaging agent, necessitating protective mechanisms such as proline accumulation. Conversely, proline levels were significantly elevated under all stress treatments compared to the control (Fig 8B), highlighting proline’s role as a universal osmoprotectant and redox regulator. Particularly strong proline accumulation was observed under heat and cold stress after 6 hours, indicating a rapid induction of the proline biosynthetic pathway in response to extreme temperature. ROS accumulation under abiotic stress acts as a signal to trigger proline accumulation. Proline, in turn, acts as a ROS scavenger to enhance cellular redox homeostasis [67]. Controlled proline catabolism further generates ROS for signaling, forming a regulatory feedback loop in stress adaptation [68]. The correlation between elevated proline and H₂O₂ suggests that proline accumulation may function as an adaptive strategy to mitigate oxidative damage. This observation aligns with earlier findings in maize, where H₂O₂ treatment induced the rapid accumulation of proline [69], confirming that H₂O₂ acts as a signal in stress-induced proline biosynthesis. Recent studies on CRISPR-edited *Solanum lycopersicum* have shown that targeted gene editing enhances proline accumulation under drought and salinity stress [70], highlighting its protective role.

Further, this study facilitated the screening of the appropriate candidate genes for functional characterization to develop transgenic plants through the overexpression of selected members. Additionally, exploring their roles in this economically significant crop could provide insights into developing stress-resilient plant varieties, which is crucial in the face of changing climatic conditions and increasing agricultural challenges.

## Conclusion

Taken together, we were able to identify 21 proline-metabolizing genes distributed in 9 different chromosomes, underscoring their significant roles in plant stress modulation. Expression profiling revealed clear gene-specific and stress-dependent expression patterns with several PMGs showing strong upregulation (e.g., *SlP5CR* consistently induced across all stresses) while others were downregulated (*SlPDH1/2*, particularly under heat stress). Such contrasting expression highlights the tightly regulated and context-dependent roles of these genes in proline metabolism. Validation through qRT-PCR analysis confirmed these stress-specific transcriptional responses, reinforcing the reliability of the RNA-seq data. The novelty of this study lies in providing the first comprehensive genome-wide identification and expression analysis of PMGs in tomato, coupled with integrative phylogenetic, structural, and expression analysis helps identify important candidate genes for further functional studies. Overall, the differential regulation of PMGs not only improves our understanding of proline metabolism but also provides practical opportunities for developing stress-tolerant tomato varieties through molecular breeding and genome editing approaches. Such advances can contribute to crop improvement aimed at enhancing productivity under adverse environmental conditions.

## Supporting information

S1 Table Primers used in the study. (PDF)

S1 Dataset Sequences used for the phylogenetic analysis. (PDF)

## Abbreviations

PMGs: proline metabolizing genes
aa: amino acid
bp: base pair
CDS: coding DNA sequence
KDa: kilo dalton
pI: Iso electric point
h: hour
PDH: proline dehydrogenase
P5CDH: pyrroline 5-carboxylate dehydrogenase
P5CS: pyrroline 5-carboxylate synthase
P5CR: pyrroline 5-carboxylate reductase
OAT: ornithine aminotransferase
P5C: pyrroline 5-carboxylate
H_2_O_2_: hydrogen peroxide
Ka: nonsynonymous rate
Ks: synonymous rate
Mya: million years ago
