# Supplementary material for: Comprehensive analysis of proline metabolizing genes reveals their functional diversification and abiotic stress response in Solanum lycopersicum
Source: PLoS One. 2025 Oct 27;20(10):e0335608. doi: 10.1371/journal.pone.0335608 (PMC12558507; doi:10.1371/journal.pone.0335608)
Supplement: S1 Table — (PDF) [file pone.0335608.s001.pdf]

S1 Table: Primers used in the study

No.	Oligo Name	5` - Oligo Seq - 3`	Tm	Product size
1	SlP5CS1_F	ACGCGGAAAGCTCCTTATGA	58	141
2	SlP5CS1_R	GGTTGGAGGGCCAGTGTAAA	59	
3	SlP5CS2_F	TGGAAGGCATGTTATTGCCC	57	146
4	SlP5CS2_R	CACCAAGTCCAAAGCGGAATC	58	
5	SlP5CR_F	GTCTCCGGTATGAGCAAGCA	59	70
6	SlP5CR_R	CAATGGTTGTACCCCCAGGT	59	
7	SlPDH1_F	TTGCGAAAAAGGCTGCAGAG	58	141
8	SlPDH1_R	GCGTGTGTTTGCTCAATGCT	58	
9	SlPDH2_F	AAAACTTGCTGCATCCAGGG	58	118
10	SlPDH2_R	TCCTGCATTTCTCAGCCCAA	58	
11	SlP5CDH2_FOR	TGGGGTTGACAATTATCTGGC	57	95
12	SlP5CDH2_REV	TTCGAAGAAGCACTAGAAAATTTGG	54	
13	SlP5CDH3_FOR	TGTTTTGGCAATGGTGTCCA	57	70
14	SlP5CDH3_REV	TCTCATCTTCCACTGCCGAA	57	
15	SlOAT5_FOR	CCAGGAAGGCTCTAATGCCC	60	82
16	SlOAT5_REV	GGAGACTGTTGCTGGCTTCT	59	
17	SlOAT8_FOR	GATTTGCTCGCACAGGAAGC	59	113
18	SlOAT8_REV	TACCACAGCTCCAAGGGGTA	59	
19	SlEF1α_FOR	ACAGGCGTTCAGGTAAGGAA		
20	SlEF1α_REV	GAGGGTATTCAGCAAAGGTCTC		
