# Supplementary material for: Comprehensive analysis of proline metabolizing genes reveals their functional diversification and abiotic stress response in Solanum lycopersicum
Source: PLoS One. 2025 Oct 27;20(10):e0335608. doi: 10.1371/journal.pone.0335608 (PMC12558507; doi:10.1371/journal.pone.0335608)
Supplement: S1 Dataset — (PDF) [file pone.0335608.s002.pdf]

S1 Dataset. Sequences used for the phylogenetic analysis

Solanum Lycopersicum
OAT:
>SlOAT1
MALQRKLINGCAGITKLRWFSNEGLRHFSGQVAAPTELPPFDYQPKPYKGPLADAVLEKRRKFLGPSLFYFYEKPLN
IVEGKMQYLYDENGKRYLDAFAGIVTVSCGHCHPEVLNAIIEQSKLLQHATTIYLHHAIADFAEALASKMPGNLKVV
YFVNSGTEANELAMLMARLYSGHLNMIALRNAYHGGSSNTIGLTALNTWKYPIPQGEIHHVMNPNPYRGAFGSDAKR
YAEDVQNHIDHGTSGKVAGFIAETIQGVGGTVELAPGYLKLVYDIVRKAGGVCIADEVQTGFGRTGSHYWGFQTQGV
TPDIVTMAKGIGNGLPLGAVVTTPEIASVMAQKIQFNTYGGNPVCSAGGHAVLKVIEKEQRQKHCAEVGSHLFGRLR
DLEKRYDIIGDVRGRGLMVGIELVTDRKEKTPAKAETGVLFEKLKDLGVLVGKGGIHGNVFRIKPPMCFSKDDADFL
VDALDYSLSKL
>SlOAT2
MAAVNGVGLSWPSKLTKNQTPKWGFSPSHRRCNPSSSSSSATIRMTASVDEKKKTFTLEKSEEAFSKAKELMPGGVN
SPVRAFKSVGGQPIIIDSVKGSRMRDIDGNEYIDYVGSWGPAIIGHADDEVLAALAETMKKGTSFGAPCLLENTLAE
MVISAVPSIEMVRFVNSGTEACMGVLRLARAFTCRPKIIKFEGCYHGHADPFLVKAGSGVATLGLPDSPGVPKAATI
DTLTAPYNDISAVESLFEEHKGEIAAVILEPVVGNAGFIPPKLEFLAAIRKITKENDALLIFDEVMTGFRLAYGGAQ
EYFGITPDLTTLGKIIGGGLPVGAYGGRRDIMEMVAPAGPMYQAGTLSGNPLAMTAGIHTLKRLQGQGTYEHLDKIT
AELTQGILDAGKKTGHAMCGGSIRGMFGFFFADGPIYNFSDAKKSDTEKFGRFYRGMLEEGVYFAPSQFEAGFTSLA
HTPEDIQRTVAAAEKVLKQI
>SLOAT3
MAKISRLFGSTVKAAITAQAGFHGKRIPAVSSLQEHIVKSTPARYNSTQACLENDISGTDNKGFKGHDMLAPFTAGW
QSTDVDPLIIEKSEGSHVYDMQGRKYIDTLAGLWCTALGGNEPRLVDAATKQLNTLPFYHSFWNRTTKPSLDLAKEL
LDMFTAKKMAKAFFTNSGSEANDTQVKLVWYYNNALGRPNKKKFIARAKAYHGSTLISASLTGLPALHQNFDLPAPF
VLHTDCPHYWRYHLPGETEEEFSTRLAKNLEDLILKEGPETIAAFIAEPVMGAGGVIPPPATYFDKIQAVVKKYDIL
FIADEVICAFGRLGTMFGSDMYNIKPDLVTLAKALSSAYMPIGAVLVSPEVSDVIHSQSNKLGSFSHGFTYSGHPVA
CAVALEAIKIYKERNMVERVNRISPKFQEGLKAFSDSPIIGEIRGLGLILATEFANNKSPNDPFPPEWGVGAYFGAQ
CQKNGMLVRVAGDTIMMSPPFVVTPEELDELIRIYGKALRETEKRVEELKSQK
>SlOAT4
MSLPTTLFAGILRRCRRRNSVRRFISTHSQPALPSIEYPLSHPIYTIWAANTSLGKTLVSAGLSTSFLSFPHRKFLY
LKPVQTGFPEDSDSRFVYNKYSEFFSQNRPEYSVFASNHVLKASVSASEAVYLGGFEIERDKDMSCGNGVVNLGLYE
ESKLQGSENWEMKGFSKLVCKTMYGWKEAVSPHLVAERENARVEDDELLGMLKGCLGSESESESEKGEVLCVIETAGGV
ASPGPSGSLQCDLYRPFRFPAILVGDGKLGGISGTISAYESLILRGYDVVAVIFEDHGLVNEGPLSSYLRRRVPVLV
LPPVPKEMSNNLMEWFQEALPTFHSLEEIMQSAFLERTSRLRNMPRKAHDVFWWPFTQHKLVPEENVTVIDSRCGEN
FAVHKVNNNVDLITQQFDACASWWTQGPDATLQIELARDMGYATARYGHVMFPENVYEPALECAELLLEGVGKGWAS
RVYFSDNGSTAIEIALKMALRKFLFDHKLVSDDLVAENVESCVDLKVLALRGSYHGDTLGAMEAQAPSPYTGFYQQP
WYKGRGYFLDPPTVAMHDGVWKLSLPQEIETLKPSLEDLTFSLRENIFKESRDTANLADIYHSYIWKTLQLIIDSSG
STQTGALIIEPVIQGAGGMVMVDPLFQRVLVKECRNQKIPVIFDEVFTGFWRLGAESATEFIRCKPDIACFAKLMTG
GIVPLAVTLASEAVFGAFIGDSKLQALLHGHSYTAHAVGCTAAVKSIKWFKDSKSNHNLISEAMLLRELWDSDMVRQ
ISLLPAVHRVVVLGTLCALELRAEGSNAGYASLYARSLLQKLREDGIYMRPLGNVIYLMCGPCTSPQSCSNLLNKVY
TRLEEL
>SlOAT5
MKVLAEQAQMLTLSSRAFYNDKFPVFAERITSMFGYDMVLPMNTGAEGVETALKLARKWGYMKKSIPKNEALIVSCC
GCFHGRTLAAISMSCDNEATHGFWPLLPGQLKVDFGDSVALEKLFKEKGDQIAGFLIEPIQGEAGVIIPPEGYLTAV
RDLCSKYNILMIADEIQSGLARSGRLLACDWEEVRPDVVILGKALGGGVLPVSAVLADKYVMLCIQAGEHGSTFGGN
PLASAVAIASLDVIRDEGLAERSAQMGEQLRHQLIKIQRQFPHFIKEVRGKGLFNAVELNSNSLLPVTAYDICMKLK
ERGILAKPTHDSIIRLTPPLSMSLEELQEGSNALHDVLVHDLPKMQKEKPATVSHATSNVCDRCGRDLYGSS
>SlOAT6
MSSSSTFFLTNSFSSVSGSTHLRRLFNNVRYGSGGGGGGVRIAASLNVEVQAPDLSKEVMDESNKVFVGTYARAPLV
LSSGKGCKLYDIEGREYLDLTSGIAVNALGHGDSDWIRAVTEQANVLTHVSNLYYTLPQLELAKRLVANSFADRVFL
SNSGTEANEAAIKFARKFQRFSHPDEKQPPVEFIAFSNCFHGRTMGAVALTSKEYYRSPFEPIMPGVTFLEYGNVQA
AKELIQSGKIAAVFVEPIQGEGGIYSATQEFLQALRTACDSAGSLLVFDEVQCGLGRTGHLWAHEAYGIYPDIMTLA
KPLAGGLPIGAVLVTERVAAAINYGDHGSTFAGGPLVCNAAVAVLDKISGPGFLASVAKKGQDFKELLVKKLGGNSH
VRDVRGVGLIIGIDLDVPASPLVEACQQSGLLVLTAGKGNVVRLVPPLTITEQELDHAAEILFNCLPVLDKTANN
>SlOAT7

MGEVNGFMGHNMLAPFTAGWQIDMGPLVIEKAEGSYVYDINGKKYLDSLSGLWCTSLGGSEARLIEAANKQLNTLPF
YHSFWNRTTKPSLDLAKELLDMFTARKMAKVFFTNSGSEANDTQVKLVWYYNNALGRPNKKKIIAQGNSYHGSTYLT
AGLSGLPILHQKFDLPPPRILHTQCPHYWSNHLPGETEEEFSTRLANNLENLILKEGPETVAAFIAEPVMGGAGVIV
PPATYFDKIQVVLKKYDILLIADEVICGFGRLGTMFGCDKYNIKPDLVSVAKALSGGYMPIGAVLVSPEISNVIHSE
SNKVGAFCHGFTYSGHPVACAVALEALKIYKERNILEVVNKLSTKFQEGLKAFADSPIIGEIRGTGLVLSTDFVNNK
SPNDPFPYDWAVGTYFGAQCQKYGMLVSFSGDHVNMAPPFSLTLEELDEMISIYKKALEDTEKRVEELKAMKK
>SlOAT8
MQRLVVTRAKWVGELRGLSQRCYSQLGLASQKEDDLIINPQMPHFDYSPPPYNGPSADEILSKRKEFLSPSMFYFYE
KNPLHLVHGKMQYLFDSNGRRYLDAFGGIATVSCGHCHPDVVEAIVNQTKRLQHSTILYLNNAITDFAEALASKLPG
DLKVVFFTNSGTEANELAIMMARLYTGCHDIISLRNAYHGNAAATMSTTGQSVWKFNVVQSGVHHAINPDPYRGVFG
SDGEKYAKDVEDLIQFGTSGRVAAFMSEAIQGVGGIVELAPGYLPAAYNAVRKAGGLCIADEVQSGFARTGSQFWGF
ENQGVVPDIVTMAKGIGNGIPLGAVVTTPEIAEVLCHSNYFNTFGGNPVCTSAGLAVLRVIEKENLQQNAHFVGSYL
KERLMAIKNKHEIVGDVRGRGLLLGVELVTDRKLKTPAKAETLHIMNHMKDMGVLVGKGGFRGNVFRITPPLCFTKE
DADFVADVMDCAMSKI
>SlOAT9
MAKTNGFMGHDMLAPFTAAWMIDMGPLVIDKAEGSYVYDVNGKKYLDSLSGLWCSVLGGSEPRLIEAANKQLNKLAF
YHSFWNRTTKPSLDLAKELINMFTANKMGKVFFTSSGSEANDTQVKLVWYYNNAIGRPNKKKIISRKNAYHGSTYMT
AGLSGLPSLHLKFDLPPPYILHTDCPHYWNYHLPGETEEEYSTRLANNLENLILKEGPETVAAFIAEPVMGGAGVII
PPATYFEKIQAVLKKYDILFIADEVICGFGRLGTMFGCDKYNIKPDLVSIAKALSGGYIPIGAVLVSEEISKVIMSQ
SNQLGVFCHGFTYSGHPVACAVALEALKIYKEKNITEVVNKLSPKFQEGLKAFIDSPIIGEIRGTGLVLSTEFVDNK
SPNDPFPPEWGVGTYFGSQCQKHGMLVSFSGDHVNMAPPFTLSLEELDEMISIYGKALKDTEKRVEELKSQKK
>SlOAT10
MAKITSLIGSGIVAATNQVGPHVKHIPAVGNLQKQIVSDQIQVRWSSTETSLKNDISATDVRGYKGHDMLAPFTAGW
HSTDLEPLVIQKSEGSYVYDVNGKKYLDALAGLWCTSLGGNEPRLVAAATKQLNELPFYHSFWNRSTKPSLDLAKEL
LDLFTANKMAKAFFTNSGSEANDTQVKLVWYYNNALGRPDKKKFIARTKSYHGSTLISASLSGLPALHQQFDLPAPF
VLHTDCPHFWRFHQPGETEEEFSTRLANNLENLILKEGPETIAAFIAEPVMGAGGVIPPPATYFEKVQAILKKYDIL
FIADEVICGFGRLGTMFGCEKYNIKPDLVSVAKALSSGYMPIGAVLVSPEVSDVIYSQSNKLGTFSHGFTYSGHPVS
CAVALETLKIYKERNIIEQVNRISPKFQEGLKAFSDSPIIGEIRGTGLLHGTEFTDNKSPNDPFPPEWGIGAYFGAR
CEKHGVLVRVAGDNIMMSPPYILSLEEIDELIIKYGKALKDTENRVEELKSQKK

P5CS:
>SlP5CS1
MDSADPARAFVKDVKRIIIKVGTAVVTRGDGRLALGRMGSLCEQIRELTSQGFEVILVTSGAVGVGRQRLRYRKLIN
SSFADLQKPQGDLDGKACAAVGQNGLMALYDTLFSQLDVTSAQLMVTDNDFRDPDFRRQLNETVNSLLCLKVVPIFN
ENDAISTRKAPYEDSSGIFWDNDSLAALLALELKADLLVLLSDVEGLYTGPPTDPQSELIHTYVKEKHEGLITFGDK
SRVGRGGMTAKVKAAVYAAYAGIPVVITSGFANNNIIKALDGQRVGTLFHREAIKWASIGDFDAREMAVSARECARR
LQTLSSQERSKILLDIADALEAKEEEILAENEADVAAAQQAGYENALISRLAMKPGKISSLANSVRVLANMDEPVGR
ILKRTELADGIILEKTSSPLGVLLIIFESRPDALVQIASLAVRSGNGLLLKGGKEAKRSNAILHKVITSSIPPIVGE
RLIGLVTSREEIPELLKLDDVIDLVIPRGSNKLVSQIKAATKIPVLGHADGICHVFIDKSADLDMAKRIVLDAKTDY
PAACNAMETLLVHEDLVQTGGLNDLILELQEKGVSLFGGPKASSVLNIPEANSFHHEYGALACTVEIVEDVNTAIEH
IHRHGSAHTDSIITEDKEVAELFLRQVDSAAVLHNASTRFSDGFRFGLGAEVGISTSRIHARGPVGVEGLLTTRWLA
RGSGQVVDGDKEIVYTHKDLNLEA
>SlP5CS2
QSQTHIRLEKWVKEVDCTFLLLHFSSSLEMETVDSTRAFVKNVKRLIVKVGTAVVTRADGRLALGRLGALCEQLQEL
NSQGYEVILVTSGAVGVGRQRLRYRKLLNSSFLDLQKPQTELDGKACAAVGQNGLMALYDSLFSQLDVTSAQLLVTD
NDFRDPDFRRQLNDTVNSLLSLKVIPIFNENDAISTRRAPYEDSSGIFWDNDSLAALLALELKADLLVLLSDVDGLY
SGPPRDPDSKLIYTYIKEIHERVITFGDKSRVGRGGMTAKVKAAMYAAYAGIPVVITSGFATDNIIKVLHGERIGTL
FHCDANKWASIGETDAREMAVAARACSRRLQALSSQERSKILQDIADALEANEKAILAENEADVVAAQQAGYEKSLI
SRLALNPGKISSLANSVRVLSNMDEPLGHTLKRTEIADGFILEKSSSPLGVVLIIFESRPDALVQIASLAVRSGNGL
MLKGGKEAKRSNAILHKVITSAIPVSVGERLIGLVTSREEIPELLKLDDVIDLVIPRGSNKLVSQIKASTKIPVLGH
ADGICHVYVDKSADMDMAKRITVDAKIDYPAACNAMETLLVHKDLAQNGGLNDLIVELQTKGVSLYGGPKASSLLMI
PEARTFRHEYSSLACTVEVVEDVYAAIDHIHQHGRHVIAHTDSIITEDQEVAEVFLRQVDSAAVFHNASTRFSDGFR
FGLGAEVGISTGRIHARGPVGVEGLLTTKWLARGSGQIVDGDKSIVYSHKDLTQQG
P5CR:
>SlP5CR
MANVCPIPTDSYKLGFIGAGKMAESIARGVVKSGILPASRIRTAHSGSARRTAFESIGVTVLDNNSQVVEDSDVIIF
SVKPQVVKNVVSELKPILSEKKLLVSVAAGIKLKDLQEWAGQGRFIRVMPNTPSAVGEAATVITLGEKATTEDGELI

SQLFGAIGKVWKADEKLFDAVTGLSGSGPAYVFLAIEALADGGVAAGLPRELALGLASQTVLGAASMVSGMSKHPGQ
HKDDVASPGGTTIAGIHELEKSGFRGILMNAVVAAAKRSKELSQN

PDH:

>SlPDH1
MANKVFCPKLLKNLGFHVRRLNSAPSPLSAVPPLNFTGDFNAVEPAQQINTTLHHHHDHHNIINFDDVKELFYGVPT
TKLIRSSMTLQMAAIDPMVDLGMWVMNSKLMEMPIFREVILGFVKNTFYEHFCAGKDLTEARRTVTNLSDSGLKAML
DYGVEHATDNESCEQSTAAFIQTIESTKSLPESSASFVVAKITAICTPRLLKRMSDLLRWEQKDPSFNLPWKRESLP
LFAESSPVYHTCSKPEPLSVEEERDLQLAHERLRKICEKCLEHEVPLLIDAEDTTIQPAIDYFAYSAAIKYHKDDQP
LIFGTIQAYLKDAKERMVIAKKAAEKMGVPMGFKLVRGAYMCSEKELASTLGFNSPIHDSIEQTHACFNSCAEFMIE
EIANGSGAVVLATHNIESGKLAATKAIDLGIKDERQNLQFAQLYGMADGLSFGLRNAGFQVSKYLPFGPVEQIMHYL
MRRAEENRGMLSTSAFDRQLMRKELSRRFEVATS

>SlPDH2
MANKVVCPKVFRDLRRFARCLNTAPTVPPMNFTGNYGSTNVTIPTLQPTDQILVNPEKKVLNFDDVKELFTGVSTSK
LIRSSLTLQMASIESMVDLGIWVMNSKFMRMPVFKEVILGFVKRTFYEHFCAGKDLIEVGKTVSKLSSLDLKGMLDY
GVEHAMDNESCDRSMNVFLQTAELTKSLPSSSVSFVVVKITAICTPKLLKRMSDLLRWEQKDPSFNLPWKQKTLPLF
AESSPFYHTLKRPEPLTIEEERDLQLGRDRLEKICKKCLELDVELLIDAEDTAIQPAIDYLAYSAAIKYHKEDHPLL
FGTIQAYLKDSKERMIIAKKAAEKMGVPMGFKLVRGAYMSSENQLASSLGFQSPIHDSIEYTHNCYNSCAEFMFDEI
ANGSGAVVLATHNIESGKLAASRAIDLGIRKDSKKLQFAQLYGMAEGLSFGLRNAGFQVSKYLPFGPVEQVMPYLIR
RAEENRGLLSTSAFDRQLMRKELIRRFDVATA

P5CDH:

>SlP5CDH1
MENFVKPQFFSFLREEAAAVVQRYSPHHKFMAQSGFGNQFAESTSSSADDVQDPEVSDSESSVVGSVHEERSSGLMR
IDEGDKIYGIISNKFLSGLGCFGLSTEITAIQKETCSSFVKQAKLQSFLIFSKAVEKKCSGNANVKYAWFGGSKDEI
SNIFSHGFSCRSNNGAYSQAICLSPDDNSHDCLQAAVPDKNGVRHLLLCRVILGKTEVVHPGSGQCHPSSEEYDTGV
DNLSSPRKFIVWSTHMNSYVFPEFMVSFRVSSHAKESQRNAVPIQNPKSPWITFPALISALSKFLPPQTVKLITKYH
NDHKGRKITRRDLIQQVRKLAGDELLTAIIKSCKNKQSKGSTGNSSSTSSINFEQRDGGCCACHRSC

> SlP5CDH2
MNQFGDHNDQVSMTIDDEKMLLSFDSQVESPSSTYHDSLRSFKKNGMIELEERNKEHDLIKAGFLSGMGQLGKEVEV
VAIHKNSCSTILGQARLESFRIYSEAMRKKCGGNANIKYAWFGSSKDEICNIISHGFSTITEPKSGECFGMGVHLYP
ANIHGVLSALEDENGLRHMLLCRVILGNTEIIEASSKQFQPTCQDFDSGVDNYLAPKTYIIWPSYMNSHILPNFLVL
LRKSRRFLPKSNSRIKFHDLLRVLSKYLHPSRMVLISKYYEDFQKNKITKLVLVRKLRRIAGDTSLRAVMKLYPNTT
I

> SlP5CDH3
MDQLGNDDQVSMTIENHEMLFSSDSEVESPSPRTNYHFRSLKSNEVMKLEEGDQDHDLIKAGFLSGMGQLGNEIEVV
AVHKNSCSTILGQARSESFRIYSEAMRKKCSGDANIKYAWFGSSKDEICNIISHGFSTIMEPNYGDCFGNGVHLYPA
NIDGVFSAVEDENGLRHMLLCRVILGNTEVIEASSKQFQPTCQDFDSGVDNYLAPKTYIIWPSNMNSHIYQIFLVSF
KGPLSLGTSLKIKIPLKPTPRIKFSNLLRVLSNFLHPSRMVLITKKYEDFQKNKISKMIFVQKLRQIAGDTSLLKSC
HDIVSKH

> SlP5CDH4
MEPKGVGFLDSGRRTVVGEKSKVVTQNLAHLLRASSEKISIQSNCDRKLEKRKRIVHCESNSQSHLRISEHKNYLNF
KRSRLPLRVLFYQNGEWTDFPQDIIPIVKEDFRAKKTVIEVKVCDFHIILDILHMVQIDVINGLQKPIAWIDEVGRC
FFPESYLISSEMLGNFETLSKRTEEFMTTEPDRITDMKLQLDTDLNGLDNRNLEEDVEESNIGYKRNKVCPLKDSQE
VADYKKSDAKIAQVAENKQNQETPSPDLEASLKFVNAESVKNMFIMGMNVNPNKCEIKINKCSSNYLTTRLELFEKQ
VEITQKYRGNSNVRYAWLAASKDLISTIMKYGLAPGGSKYRPKFGVGVHLSALHCASKSAINCDADENGVHYMVFAR
VILGNMEPLHCGSEQWHPSDEKYDSGVDDLENPTHYVIWNMNLNTHIYPVCVVSFRIPPGAEGPRVGNDSRIDVSGV
NTCPRGPVEQGSHRFQVSLVKLALEEAARIPKSSGVPFSLLIDAISNVVNAEKMNHVTRSYELLGFKKICRDEFEKR
LVAIVGLTLLKSTTKSLLCKIQSKPIEMVQPKQEPQSADFKGNTALFHWIEPQTE

> SlP5CDH5
METKFEKVLDRSRNIVVDLKRKREARCETHLSGATHVILPVRPLLNSSIRPLGKRTKLNGSRSRCGGYKFHQKESLL
RYYLNFRRSGLPQRLMYYQNSQWTDFPENIVSMAKQDLRTKRSAREVVFNGNNYVLDFFHMMLLDLKSGMQQPIAWI
DEAGKCFFPEVFAYCDELHEPCHCEDNDCVDVDSETEGSNDLELRLEIEVNGADISSSEESSGESNANFEQVNFCHE
PAAKNRIAEVGDDHVRISDTKAKDDSVENYQMVENAVGRYDSKWKHMDPKAVSEMFLKGISSSASANIIDLQRISSS
FMEVRKELFQKQVEILRKHRGDATVSYAWLPSSKGMITSIMKYGLANYYPSRTNSSYGIGVHLFPANCSEISAKCSD
VDENGVQYMVLCRVIMGNMEVVCPGSKQFHPSSEDFDNGVDSVENPKCYVVWTMNTSTHIFPEYVVSFKFSSDSEGY
LVGNRSPNVSAVTSCQGPADKVPADTLPAVLGSDCYQNSLGLASKKASRTPKSPWMPFPMLFAAISRKVRQEDMNLV
CSNYELFKGKKINRDEFVRKLRLIVGDTLLRSTITSLQCQVPPKSMEMVSVKQEQESICLE

> SlP5CDH6
MESNWVEVLDNDRRTADVDSKSKVASQYVARIVGAGTEKLEELSFQPNCSSFYSTLGKRKRTVEGETNCRSHLRKST
VKNYSNFIKSGLPQRVLYHQNGEWNDFPQDIVRIVKEDFRAKKAVIEVNFGGFHVILDILYMVQVNLVTGLEKPIAW
IDEAGGCFFPDLCLVSCKAHDNIEIQSQRTEGFSAAEPDRATNIKLHLEIDINGPNNCKLDEGMEESNVRVKRIKVD
PLKDNQQFAVDQKSYPKMEVVAENQQDQESLSPILEDTLKLVDAESVKTMFVKGMNTIYMVDIVKISQCSSKYMRNR
LELFEKQAEITQKYRGKSNVRHAWLAASKEVVSTIMNYGLMHGACGQKTKLGVGVHLIAQDCASKSAASCDIDENGI
RYMVLCRVILGNEELLHFGSHQSYPSNERYDSGVDDLESPTHYTVWNMNMNTHIYPEYVVSFRMSSGAEGAPIKEES
RLDVSRVTSQGSEERLDLNKLPTELGNCQQYQFVRNIQQSHGVGVGTNKSPKSPWMPFSMLFGAISAKVSPKDLKLV
HAHYDLFRSKKISRNDFIRRLRLIVGDQLLKSTITNLQCKPPSASPCFLNVPSGESNC

Arabidopsis thaliana
AtOAT:
>AT5G46180.1
MAATTRRLLYYVSKRFSTAGVRRSYGGLPQSNSKSPPSSSQRLMELESEFSAHNYHPVPVVFSRANGSTIWDPEGKR
YIDFLAAYSAVNQGHCHPKIMKALQEQVEKLTLSSRAFYNDKFPVFAERLTNMFGYDMVLPMNTGAEGVETALKLAR
KWGHEKKNIPKDEAIIVSCCGCFHGRTLAIVSMSCDNDATRGFGPLLPGNLKVDFGDADSLEKIFKEKGDRIAGFLF
EPIQGEAGVIIPPDGYLKAVRELCTKYNVLMIADEVQSGLARSGKMLACDWEEIRPDMVILGKALGGGVIPVSAVLA
DKDVMLHIKPGQHGSTFGGNPLASAVAMASLDVIVEEKLVERSASLGEELRIQLNEIKKQFPKYIKEVRGRGLFNAI
EFNSESLSPVSAYDICLSLKERGVLAKPTHNTIVRLTPPLSISSDELRDGSEALHDVLELDLPNLLKINSGKTPVSH
ITECDRCGRNLYA

AtP5CS1:
>AT2G39800.1
MEELDRSRAFARDVKRIVVKVGTAVVTGKGGRLALGRLGALCEQLAELNSDGFEVILVSSGAVGLGRQRLRYRQLVN
SSFADLQKPQTELDGKACAGVGQSSLMAYYETMFDQLDVTAAQLLVNDSSFRDKDFRKQLNETVKSMLDLRVIPIFN
ENDAISTRRAPYQDSSGIFWDNDSLAALLALELKADLLILLSDVEGLYTGPPSDPNSKLIHTFVKEKHQDEITFGDK
SRLGRGGMTAKVKAAVNAAYAGIPVIITSGYSAENIDKVLRGLRVGTLFHQDARLWAPITDSNARDMAVAARESSRK
LQALSSEDRKKILLDIADALEANVTTIKAENELDVASAQEAGLEESMVARLVMTPGKISSLAASVRKLADMEDPIGR
VLKKTEVADGLVLEKTSSPLGVLLIVFESRPDALVQIASLAIRSGNGLLLKGGKEARRSNAILHKVITDAIPETVGG
KLIGLVTSREEIPDLLKLDDVIDLVIPRGSNKLVTQIKNTTKIPVLGHADGICHVYVDKACDTDMAKRIVSDAKLDY
PAACNAMETLLVHKDLEQNAVLNELIFALQSNGVTLYGGPRASKILNIPEARSFNHEYCAKACTVEVVEDVYGAIDH
IHRHGSAHTDCIVTEDHEVAELFLRQVDSAAVFHNASTRFSDGFRFGLGAEVGVSTGRIHARGPVGVEGLLTTRWIM
RGKGQVV
DGDNGIVYTHQDIPIQA
>AT2G39800.2
MAYYETMFDQLDVTAAQLLVNDSSFRDKDFRKQLNETVKSMLDLRVIPIFNENDAISTRRAPYQDSSGIFWDNDSLA
ALLALELKADLLILLSDVEGLYTGPPSDPNSKLIHTFVKEKHQDEITFGDKSRLGRGGMTAKVKAAVNAAYAGIPVI
ITSGYSAENIDKVLRGLRVGTLFHQDARLWAPITDSNARDMAVAARESSRKLQALSSEDRKKILLDIADALEANVTT
IKAENELDVASAQEAGLEESMVARLVMTPGKISSLAASVRKLADMEDPIGRVLKKTEVADGLVLEKTSSPLGVLLIV
FESRPDALVQIASLAIRSGNGLLLKGGKEARRSNAILHKVITDAIPETVGGKLIGLVTSREEIPDLLKLDDVIDLVI
PRGSNKLVTQIKNTTKIPVLGHADGICHVYVDKACDTDMAKRIVSDAKLDYPAACNAMETLLVHKDLEQNAVLNELI
FALQSNGVTLYGGPRASKILNIPEARSFNHEYCAKACTVEVVEDVYGAIDHIHRHGSAHTDCIVTEDHEVAELFLRQ
VDSAAVFHNASTRFSDGFRFGLGAEVGVSTGRIHARGPVGVEGLLTTRWIMRGKGQVVDGDNGIVYTHQDIPIQA
>AT2G39800.3
MEELDRSRAFARDVKRIVVKVGTAVVTGKGGRLALGRLGALCEQLAELNSDGFEVILVSSGAVGLGRQRLRYRQLVN
SSFADLQKPQTELDGKACAGVGQSSLMAYYETMFDQLDVTAAQLLVNDSSFRDKDFRKQLNETVKSMLDLRVIPIFN
ENDAISTRRAPYQDSSGIFWDNDSLAALLALELKADLLILLSDVEGLYTGPPSDPNSKLIHTFVKEKHQDEITFGDK
SRLGRGGMTAKVKAAVNAAYAGIPVIITSGYSAENIDKVLRGLRVGTLFHQDARLWAPITDSNARDMAVAARESSRK
LQALSSEDRKKILLDIADALEANVTTIKAENELDVASAQEAGLEESMVARLVMTPGKISSLAASVRKLADMEDPIGR
VLKKTEVADGLVLEKTSSPLGVLLIVFESRPDALVQIASLAIRSGNGLLLKGGKEARRSNAILHKVITDAIPETVGG
KLIGLVTSREEIPDLLKLDDVIDLVIPRGSNKLVTQIKNTTKIPVLDGICHVYVDKACDTDMAKRIVSDAKLDYPAA
CNAMETLLVHKDLEQNAVLNELIFALQSNGVTLYGGPRASKILNIPEARSFNHEYCAKACTVEVVEDVYGAIDHIHR
HGSAHTDCIVTEDHEVAELFLRQVDSAAVFHNASTRFSDGFRFGLGAEVGVSTGRIHARGPVGVEGLLTTRWIMRGK
GQVVDGDNGIVYTHQDIPIQA
>AT2G39800.4
MEELDRSRAFARDVKRIVVKVGTAVVTGKGGRLALGRLGALCEQLAELNSDGFEVILVSSGAVGLGRQRLRYRQLVN
SSFADLQKPQTELDGKACAGVGQSSLMAYYETMFDQLDVTAAQLLVNDSSFRDKDFRKQLNETVKSMLDLRVIPIFN

ENDAISTRRAPYQDSSGIFWDNDSLAALLALELKADLLILLSDVEGLYTGPPSDPNSKLIHTFVKEKHQDEITFGDK
SRLGRGGMTAKVKAAVNAAYAGIPVIITSGYSAENIDKVLRGLRVGTLFHQDARLWAPITDSNARDMAVAARESSRK
LQALSSEDRKKILLDIADALEANVTTIKAENELDVASAQEAGLEESMVARLVMTPGKISSLAASVRKLADMEDPIGR
VLKKTEVADGLVLEKTSSPLGVLLIVFESRPDALVQIASLAIRSGNGLLLKGGKEARRSNAILHKVITDAIPETVGG
KLIGLVTSREEIPDLLKLDDVIDLVIPRGSNKLVTQIKNTTKIPVLGHADGICHVYVDKACDTDMAKRIVSDAKLDY
PAACNAMETLLVHKDLEQNAVLNELIFALQSNGVTLYGGPRASKILNIPEARSFNHEYCAKACTVEVVEDVYGAIDH
IHRHGSAHTDCIVTEDHEVAELFLRQVDSAAVFHNASTRFSDGFRFGLGAEVGVSTGRIHARGPVGVEGLLTTRWIM
RGKGQVVDGDNGIVYTHQDIPIQA

AtP5CS2:
>AT3G55610.1
MTEIDRSRAFAKDVKRIVVKVGTAVVTGKGGRLALGRLGAICEQLAELNSDGFEVILVSSGAVGLGRQRLRYRQLVN
SSFADLQKPQMELDGKACAGVGQSSLMAYYETMFDQLDVTVAQMLVTDSSFRDKDFRKQLSETVKAMLRMRVIPVFN
ENDAISTRRAPYKDSTGIFWDNDSLAALLSLELKADLLILLSDVEGLYTGPPSDSTSKLIHTFIKEKHQDEITFGEK
SKLGRGGMTAKVKAAVNAAYGGVPVIITSGYAAENISKVLRGLRVGTLFHQDAHLWAPVVDTTSRDMAVAARESSRK
LQALSSEDRKQILHDIANALEVNEKTIKAENDLDVAAAQEAGYEESLVARLVMKPGKISSLAASVRQLAEMEDPIGR
VLKKTQVADDLILEKTSSPIGVLLIVFESRPDALVQIASLAIRSGNGLLLKGGKEARRSNAILHKVITDAIPETVGG
KLIGLVTSREEIPDLLKLDDVIDLVIPRGSNKLVSQIKNSTKIPVLGHADGICHVYVDKSGKLDMAKRIVSDAKLDY
PAACNAMETLLVHKDLEQNGFLDDLIYVLQTKGVTLYGGPRASAKLNIPETKSFHHEYSSKACTVEIVEDVYGAIDH
IHQHGSAHTDCIVTEDSEVAEIFLRQVDSAAVFHNASTRFSDGFRFGLGAEVGISTSRIHARGPVGVEGLLTTRWIM
RGKGQVVDGDNGIVYTHKDLPVLQRTEAVENGI
>AT3G55610.2
MTEIDRSRAFAKDVKRIVVKVGTAVVTGKGGRLALGRLGAICEQLAELNSDGFEVILVSSGAVGLGRQRLRYRQLVN
SSFADLQKPQMELDGKACAGVGQSSLMAYYETMFDQLDVTVAQMLVTDSSFRDKDFRKQLSETVKAMLRMRVIPVFN
ENDAISTRRAPYKDSTGIFWDNDSLAALLSLELKADLLILLSDVEGLYTGPPSDSTSKLIHTFIKEKHQDEITFGEK
SKLGRGGMTAKVKAAVNAAYGGVPVIITSGYAAENISKVLRGLRVGTLFHQDAHLWAPVVDTTSRDMAVAARESSRK
LQALSSEDRKQILHDIANALEVNEKTIKAENDLDVAAAQEAGYEESLVARLVMKPGKISSLAASVRQLAEMEDPIGR
VLKKTQVADDLILEKTSSPIGVLLIVFESRPDALVQIASLAIRSGNGLLLKGGKEARRSNAILHKVITDAIPETVGG
KLIGLVTSREEIPDLLKLDDVIDLVIPRGSNKLVSQIKNSTKIPVLGHADGICHVYVDKSGKLDMAKRIVSDAKLDY
PAACNAMETLLVHKDLEQNGFLDDLIYVLQTKGVTLYGGPRASAKLNIPETKSFHHEYSSKACTVEIVEDVYGAIDH
IHQHGR

AtP5CR:
>AT5G14800.1
MEILPIPAESFKVGFIGAGKMAESIARGVVASGVLPPNRICTAVHSNLNRRDVFESFGVNVFSTSEEVVKESDVVIF
SVKPQVVKKAVTELKSKLSKNKILVSVAAGIKLNDLQEWSGQDRFIRVMPNTPAAVGEAASVMSLGTGATEEDGAIV
AMLFGAVGKILKADEKMFDAVTGLSGSGPAYIFLAIEALADGGVAAGLPRELALSLASQTVLGAATMVSKTGKHPGV
LKDDVTSPGGTTIAGVHELEKGSFRATLMNAVVAAAKRSRELSQS
>AT5G14800.2
MEILPIPAESFKVGFIGAGKMAESIARGVVASGVLPPNRICTAVHSNLNRRDVFESFGVNVFSTSEEVVKESDVVIF
SVKPQVVKKAVTELKSKLSKNKILVSVAAGIKLNDLQEWSGQDRFIRVMPNTPAAVGEAASVMSLGTGATEEDGAIV
AMLFGAVGKILKADEKMFDAVTGLSGSGPAYIFLAIEALADGGVAAGLPRELALSLASQTVSSWSCNDG

AtPDH1:
>AT3G30775.1
MATRLLRTNFIRRSYRLPAFSPVGPPTVTASTAVVPEILSFGQQAPEPPLHHPKPTEQSHDGLDLSDQARLFSSIPT
SDLLRSTAVLHAAAIGPMVDLGTWVMSSKLMDASVTRGMVLGLVKSTFYDHFCAGEDADAAAERVRSVYEATGLKGM
LVYGVEHADDAVSCDDNMQQFIRTIEAAKSLPTSHFSSVVVKITAICPISLLKRVSDLLRWEYKSPNFKLSWKLKSF
PVFSESSPLYHTNSEPEPLTAEEERELEAAHGRIQEICRKCQESNVPLLIDAEDTILQPAIDYMAYSSAIMFNADKD
RPIVYNTIQAYLRDAGERLHLAVQNAEKENVPMGFKLVRGAYMSSEASLADSLGCKSPVHDTIQDTHSCYNDCMTFL
MEKASNGSGFGVVLA
THNADSGRLASRKASDLGIDKQNGKIEFAQLYGMSDALSFGLKRAGFNVSKYMPFGPVATAIPYLLRRAYENRGMMA
TGAHDRQLMRMELKRRLIAGIA

AtPDH2:
>AT5G38710.1

MANRFLRPNLIHRFSTVSPVGPPTTIIPEILSFDQPKPEVDLDLSDQARLFASVPISTLLRSTAILHATSIGPMVDL
GSWLMSSKLMDTTVTRDLVLRIVKGTFYDHFCAGEDAAAAARRVSSVYESTGLKGMLVYGVEHAEDGGACDENIQKF
IETVEAAKTLPSSHLSSVVVKITAICPMNVLKRVSDLLRWQYKNPNFKLPWKLNSFPVFSGLSPLYHTTSEPEPLTV
EEERELEKAHERLKSVCLRCQESNVPLLIDAEDTILQPAIDYMAYWSAIMFNSDKDRPIVYNTIQAYLKDAGERLHL
ALRESEKMNVPIGFKLVRGAYMSSEAKLADSLGYKSPVHDTIQNTHDCYNDCMSFLMEKASNGSGIAVILATHNTDS
GKLGARKASELGINK
ENGKIEFAQLYGMSDALSFGLKRAGFNVSKYMPYGPVDTAIPYLIRRAYENRGMMSTGALDRQLMRKELKRRVMAW

ATP5CDH:
>AT5G62520.1
MDYVRTQVEAVFDDSEQDGSTISESGSCDSSSDRSFADELGLMELLEGDKAHDLIYRNCK SGLGDQCQIL SVLRN
GFRNV GSRAKLKTFQVFQEAVQMKHGGDGGAKVKYGWCSVSKHELKTIFEYGFSEPLRNDGSFGRGLYLSPDNSPL
DCLKDSASESEDGMRFLLLCRVLLGKSEIVPQGSTRSCPSSPEFDSGVDDLVSTKKYIVWSTHMNTHVLPEFLVCIK
APFNLTRSPKRLRSPWMAFPVLIKALSKFLPPSQILVIQKHYKDQQNRRITRSELIQRVRSITGDKLLVHIIKACGH
KVQH

Oryza Sativa
OsOAT:
>LOC_Os03g44150.1
MAAALARRGGGGLARALARGRGMCSATAAERAAGAALTSEELMRMERERSAHNYHPIPVVFSKGEGSHILDPEGNKY
IDFLSAYSAVNQGHCHPKVLRALKEQAERLTLSSRAFYNDKFPIFAEYLTSMFGYEMMLPMNTGAEGVETAIKLVRK
WGYEKKKIPKNEALIVSCCGCFHGRTLGVISMSCDNDATRGFGPLVPGHLKVDFGDTDGLEKIFKDHGERICGFLFE
PIQGEAGVIIPPDGYLKAVRDLCSRHNILMIADEIQTGIARTGKMLACDWENIRPDVVILGKALGAGVVPVSAVLAD
KDIMLCIKPGEHGSTFGGNPLASAVAVASLKVVTDEGLVERAAKLGQEFRDQLQKVQQRFPQIIREVRGRGLLNAVD
LSNEALSPASAYDICIKLKERGVLAKPTHDTIIRLAPPLSISPEELAEASKAFSDVLEHDLPQLQKQIKKTESAAEK
QSCDRCGRDLY

OsP5CS1:
>LOC_Os05g38150.1
MASVDPSRSFVRDVKRVIIKVGTAVVSRQDGRLALGRVGALCEQVKELNSLGYEVILVTSGAVGVGRQRLRYRKLVN
SSFADLQKPQMELDGKACAAVGQSGLMALYDMLFNQLDVSSSQLLVTDSDFENPKFREQLTETVESLLDLKVIPIFN
ENDAISTRKAPYEDSSGIFWDNDSLAGLLALELKADLLILLSDVDGLYSGPPSEPSSKIIHTYIKEKHQQEITFGDK
SRVGRGGMTAKVKAAVLASNSGTPVVITSGFENRSILKVLHGEKIGTLFHKNANLWESSKDVSTREMAVAARDCSRH
LQNLSSEERKKILLDVADALEANEDLIRSENEADVAAAQVAGYEKPLVARLTIKPGKIASLAKSIRTLANMEDPINQ
ILKKTEVADDLVLEKTSCPLGVLLIVFESRPDALVQIASLAIRSGNGLLLKGGKEAIRSNTILHKVITDAIPRNVGE
KLIGLVTTRDEIADLLKLDDVIDLVIPRGSNKLVSQIKASTKIPVLGHADGICHVYIDKSADMDMAKHIVMDAKIDY
PAACNAMETLLVHKDLMKSPGLDDILVALKTEGVNIYGGPIAHKALGFPKAVSFHHEYSSMACTVEFVDDVQSAIDH
IHRYGSAHTDCIVTTDDKVAETFLRRVDSAAVFHNASTRFSDGARFGLGAEVGISTGRIHARGPVGVEGLLTTRWIL
RGRGQVVNGDKDVVYTHKSLPLQ
>LOC_Os05g38150.2
MASVDPSRSFVRDVKRVIIKVGTAVVSRQDGRLALGRVGALCEQVKELNSLGYEVILVTSGAVGVGRQRLRYRKLVN
SSFADLQKPQMELDGKACAAVGQSGLMALYDMLFNQLDVSSSQLLVTDSDFENPKFREQLTETVESLLDLKVIPIFN
ENDAISTRKAPYEDSSGIFWDNDSLAGLLALELKADLLILLSDVDGLYSGPPSEPSSKIIHTYIKEKHQQEITFGDK
SRVGRGGMTAKVKAAVLASNSGTPVVITSGFENRSILKVLHGEKIGTLFHKNANLWESSKDVSTREMAVAARDCSRH
LQNLSSEERKKILLDVADALEANEDLIRSENEADVAAAQVAGYEKPLVARLTIKPGKIASLAKSIRTLANMEDPINQ
ILKKTEVADDLVLEKTSCPLGVLLIVFESRPDALVQIASLAIRSGNGLLLKGGKEAIRSNTILHKVITDAIPRNVGE
KLIGLVTTRDEIADLLKLDDVIDLVIPRGSNKLVSQIKASTKIPVLGHADGICHVYIDKSADMDMAKHIVMDAKIDY
PAACNAMETLLVHKDLMKSPGLDDILVALKTEGVNIYGGPIAHKALGFPKAVSFHHEYSSMACTVEFVDDVQSAIDH
IHRYGSAHTDCIVTTDDKVAETFLRRVDSAAVFHNASTRFSDGARFGLGAEVGISTGRIHARGPVGVEGLLTTRWIL
RGRGQVVNGDKDVVYTHKSLPLQ

OsP5CS2:
>LOC_Os01g62900.1
MGRGGIGGAGLVAAVAKADVENTDSTRGFVKDVKRIIIKVGTAVVTGPNGRLAMGRLGALCEQVKQLNFEGYEVILV
TSGAVGVGRQRLKYRKLVNSSFADLQNPQMDMDGKACAAVGQSVLMAIYDTLFSQLDVTSSQLLVTDRDFMDPSFGN
QLRETVNSLLDLKVIPVFNENDAISTRRQPYEDSSGIFWDNDSLARLLAQELKADLLIMLSDVEGLYSGPPSDPQSK
IIHTYVHEQHGKLISFGEKSRVGRGGMQAKVAAAFTASSKGIPVVIASGFAIDSIIKVMRGEKIGTLFHREANQWGC

SKEATAREMAVAARDCSRHLQKLSSEERKKILLDIADALEANEDLITSENQADLDLAQDIGYDKSLVARMTIKPGKI
KSLAGSIREIADMEDPISHTLKRTEVAKDLVFEKTYCPLGVLLIIFESRPDALVQIASLAIRSGNGLLLKGGKEAMR
SNTILHKVITGAIPDVVGKKLIGLVKNKDEIADLLKLDDVIDLVIPRGSNKLVSQIKAATKIPVLGHADGICHVYID
KSADMDMAKRIVLDAKVDYPAACNAMETLLVHKDLNRTEGLDDLLVELEKEGVVIYGGPVAHDTLKLPKVDSFHHEY
NSMACTLEFVDDVQSAIDHINRYGSAHTDCIITTDGKAAETFLQQVDSAAVFHNASTRFCDGARFGLGAEVGISTGR
IHARGPVGVDGLLTTRCILRGSGQVVNGDKGVVYTHRELPLQ

OsP5CR:
>LOC_Os01g71990.1
MAAPPQPVPAPAAASPEVFRLGFIGPGNLAESIARGVAASGVLPATAIRTAPHRRPERAEAFSSIGAHILETNAQVV
DDSDVIVISVKPQIVRQVLVELKPLLSEEKLLVSIAAGIKMEDLQGWSGHRRFIRVMPNTPSAVGQAASVMCLGEMA
TENDENRVRSLFSAIGKVWTAEEKYFDAVTGLSGSGPAYIFLAIEAMADGGVAAGLPRDLALGLASQTVLGAATMVN
KTGKHPGQLKDMVTSPAGTTITGIQELEKGAFRGTLINAVVAATKRCRELSQS

OsPDH:
>LOC_Os10g40360.1
MAIASRIQKRVLASFAAAAAAKLPEAAVAAAGGAAEAVEEVASSVQEQVQAQGAQVLEFGDTERLFAGERSTSLVRT
LAVLQALSVGPLVDVATAALRSPAVAGSAAGRAAARATAYQHFCAGETAEEAAAAVRRLWRGGMGGILDYGIEDAED
GPACDRNAAGFLAAIDVAAALPPGSASVCIKITALCPVALLEKASDLLRWQQKHPATKLPWKVHGFPVLCVSSPLYL
TAAEPPALEAEEERELEMAHGRLLAIGERCAEYDIPLLVDAEYATVQPAIDYFTFAGALAFNGGGRPIVHGTVQAYL
RDARDRLEAMARAAQGERVCLALKLVRGAYLAREARLAASLGVPSPVHRSIQDTHDCYNGCAAFLLDRVRRGAAAVT
LATHNVESGQLAAARALELGIGGGGDRGLQFAQLMGMADGLSLGLRNAGFQVSKYLPYGPVEQIIPYLIRRAEENRG
LLSSSSFDRQLLRKELVRRFKAAMLGRE

OsP5CDH:
>LOC_Os03g63770.1
MEPKNAIALDEHVLDSTIRKRKRVGATQCAEANDAPNLSQNGPKQNLALFPDSTGHKKSKMMCSADDILERYNNFKI
SGMPVRVMSHQHGGWRDFPEDVVNSVQQSFQLKRPITSAVFQNRHILLDFMQMVCLDSVMAISKPIAWIDDHGKCFS
PDSCAGVIPSEPLQHGKNEFLKSFHDLSSSYEAHEHDGMSAAESSSSASFDAVLSDVQEVNNVVEDKQKVLNESGEV
SGENKKGHLSHMNETADGAMQAPRNNQSVQRADSAVRNLLFQGSGHLFTEKDIIGIYRTPMLDQLGRSRYSLFQKEV
QVTKNQRGNANERYAWLACTKGTMEEMMMNGALEIAKPLQGPMYGVGAHLAPANSSNICVGLSDIDENGIIRMMLCR
VIMGNVEVVFPGSNQCQPTSESFDSGVDDLQRPKHYIIWDANVHKHIYAEYAVIIKVPYMNNGDTASNISEIRNSGA
LDNPTKDDSLQTIASSGDEQQACMLGRAPSPRSPSSPWMPFSMLFAAISAKVPRSDMDLVHKYYEEFKRRKISRPDL
VKQLRQIVGDKLLVSTVVRLQQKLPPMAATEQAPRAPGRGGGASP
>LOC_Os03g12820.1
MDFSGDVKPAIHRPSVAAARGGGNGGAIPLLRGWQAFRRSGAPARLLCFEGGAWADVAGEVVGLLRRAFMEGKAVCE
AACGGRVFLFDFMRMVRIDEATAEEAALGWIDDRGACFFPAPEGGRKRKRERDEAGSEVKGEDRRRRQPAAEEEDGD
EASSGVEERSGESRPEADEPDRKKARGTLWGKAVRLDEADKFYKVVEKLFVSRMAPVAAARGVAITAVHKVAQGPRA
RAFHLQGQLLAAARGVGDGSNAKFAWYGAPAADVAAAVEHGFGRTNGQFLGGRAHGDGVHLSPPQYPHASAMLTKPD
ENGEAHIVLCRVLMGRPEAVPASSPQFHPSSDEYDSAVDNLENPRWYVVWSTDMNTRILPEYVVSFRWPNLPQMEGS
SGLGSKLKKPSPAATRDMFPMLLTEIQRFVPSPKLQTLQRTYNCFKRGQMKKDQFIRFLRSHIGDNVLTTVAKKLRG
Y
>LOC_Os10g42710.1
MAAMNEKVLDKCGRNISSLKRKRDNPAARCADAGNTSKLHKHPADNSVVRFYVDEGHKAKIKCHFKMQIIQSYQNFM
TSALPKRILLRQGGEWKDFPKQIVKLAHSDFRTKKTITEGEHQTHLFLLDFVHMTFIDSKTGLQRPIAWIDENGKQY
FPEFFIEDKTLYRKKELGNGNNVYIIVEPNGTQEMNDHFGTSESSAESSNFESSTDDVSSPKRAKAERSVAGNKTGG
VKETIGENEPHALLPIPCRSLPQDKLGDHSRVQLAISAVQKLLLQGLGTVLGSKDIVGIYRTPAVDNHKEFRYNLFK
KQAEHTKCKRGNANVRYAWLACSKDAVDEMMLNGVMHFEKTVKCPDYGIGTILAPANCSNTCVNYSDVDENGIVHMM
LCRVVMGNVEIVHHGSKQHRPSNEYFDSGVDDIKNPQHYIVWDMNVNSHIYSEFVVTIKLPSRVKDSPATEEDCHNL
SEVSSLILSSGSPDSVSQDMNLQASPALGGHYEAPMLGDKVERAPSTPWMPFSMLFAAISTKVSAENMDMVNSCYEE
FKSKKISRVDLVKKLRHIVGDRMLISTIMRLQDKLPPMSRHEAPKHVGQDDG
>LOC_Os04g57640.1
MASPQESNSLCLKRKLVDDCLSKECKSRRIKTEKGPSSDSSAKRCKCCCTRPNLASDCVNYLKSGVPSRVMFYKQGS
WHNFPEQIMKSLIEEFRSNKSSVVAVMDDEPVLVDFLSMTLVNLKSRKQRSVAWFDDTGKCFYPSLFFDEEADEVAK
VGGDFEGATQGIMLDKVANSPPEVVKQVVLESSPPVPQKPATADILRKKIASVERGSEGFLFAQDLFLSGMGPFATP
NNILHIHRYSPNDITAQCRLQAFEKQMMSTKEERGDANVRYGWLGSRKNDIVRILINGFGNNGKPAEKAGLSAGVYL
SPEDRAFSSVGLCDVDEKGVQYMLLCRLILGNMEAVMPGSQDSFPSSDIYDSGVDDCSNPKCYVMWPSHLSTHIRLE

YLVSFRLSSKVRNYLLGLKGLWFHPSPKEVAVDISTLVPIMSGNAEGPTSPWISFRVLFAMIQENISSVARELLFHH
YEELKENKITREEMVKQMIILVGEKLLLETLKRLHYCPSLWYKSAGKIASSDPARTAAEDRSLDQTGNCSLIVSVAH
GDSHAPNAVAENSTSLCTKGCDTPATGMISKGYDSLAPKGVPETSTSVGPVHGASPSVEPKVRDSPIQTVLSGNIAT
DCAKRQDPLVSRVAPVAHNGLLRMPSGKSASLAAQVCNSVRPSTGPSGRASTEPNNASKSCGIFAPGIRPKGGESLV
PSLALGNSKYAGVEGLNSAPRATPPGIRPKGGESLVHGLALGNSKCAGAEGINSAPRVTPLGIRPKGGESIAPSLAL
GNSKCAGAEGINSAPRVTPPGIRPKGGESIAPSLALGNSKCAGAEGLNSAPRVTPPGIRPKGGESFVPSLALGNSKC
AGAEGLNSAPRVTPKDKEFLSLSISSQQSPVLNSGKGHDGTSGAARPVHAPGHGNPKALATEARGSLSLSIAPNVHD
PPASSKEPKDDASPIAGMVSESQHSQAPNAVTKGYNAPTPIPKESKGQHLQSGVHSQSSGPDDASSSNVARAADTIV
ALSTLREKGR

Zea mayes
P5CR:
>Zm00001eb090900
MYDVVFSLLYMAVALPFAAPAMLVTWTTVLVLLAFAGCLCRSLVAEGRRTTRDISGLALHVLLRKGNVVAVLLLGRH
DDYAGRHAS
>Zm00001eb405140
MAAPPVQPVPTAAAAAAAVNGDAFRLGFVGAGNLAESIARGVAASGVLPASAIRTAPHRRPERGEAFASFGACLLQT
NAHVVDDSDVIVISVKPQIVKQVLLQLRPRLSEKKLLVSIAAGIKMQDLQDWSGQRRIIRVMPNTPSAVGQAASVMC
LGEMATQDDENRVRKLFSAIGKVWTAEEKYFDAVTGLSGSGPAYIFLAIEAMADGGVAAGLPRDLALGLASQTVLGA
ATMVSQTGKHPGQLKDQVTSPAGTTIAGIQELEKGAFRGTLISAVVAAAKRCRELS
>Zm00001eb141220
MAAPPVQPVPTAAAAAAAVNGDAFRLGFVGAGNLAFASFGACLLQTNAHVVDDSDVIVISVKPQIVKQVLLQLRPRL
SEKKLLVSIAAGIKMQDLQDWSGQRRIIRVMPNTPSAVGQAASVMCLGEMATQDDENRVRKLFSAIGKVWTAEEKYF
DAVTGLSGSGPAYIFLAIEAMADGGVAAGLPRDLALGLASQTVLGAATMVSQTGKHPGQLKDQVTSPAGTTIAGIQE
LEKGAFRGTLISAVVAAAKRCRELS
>Zm00001eb163400
MDPAAWPPWTSLLLRALSRRRTWVALFLAVYAALLSSSWSLLASVRAWYYAASSASASAAASAAPAWPAALYASVTY
GAVFGLLSMAAALAVAAPAMLVTWTTVLVLLAFAGRRPRSLVAEGRRATRDIAGLALRVLLREGNAVAALCAAASFA
ALLLGRRDDDAARRAS

P5CDH:
>Zm00001eb294160
MYGDISAKAAHMLGQPAVLDFFAKLIQRVSPKSYQQALAEVQVSQKFLENFCGDQVRFLARSFAVPGNHLGQRSNGY
RWPYGPVAIITPFNFPLEIPLLQLMGALYMGNKPVLKVDSKVSIVMEQMIRLLHDCGLPAEDMDFINSDGAVMNKLL
LEANPKMTLFTGSSRVAEKLAADLKGRVKLEDAGFDWKILGPDVQEVDYVAWVCDQDAYACSGQKCSAQSVLFMHKN
WSSSGLLEKMKKLSERRKLEDLTIGPVLTVSQGRSQPFFGGQLTPVTTEAMIEHMNNLLKIRGSKVLFGGEPLANHS
IPKIYGAMKPTAVFVPLEEILKSGNFELVTKEIFGPFQVVTEYSEDQLELVLEACERMNAHLTAAVVSNDPLFLQDV
LGRSVNGTTYAGIRARTTGAPQNHWFGPAGDPRGAGIGTPEAIKLVWSCHREVIYDVGPVPESWALPSAT
> Zm00001eb289500
MATADQSRIFMKDVKRVIIKVGTAVVTRHDGRVSTGNFGVLCEQVKEINALGYEVIIVSSGAVGVGKQRLNYRKLVN
SSFADMQKPQTELDGKACAAVGQSGLMALYDMLFTQLDVSSSQLLVTDSDFENPNFRERLRETVESLLDLKVVPIFN
ENDAISTRKAPYEDSSGIFWDNDSLAGLLAIELKADLLVLLSDVDGLYSGPPSEPQSKMIHTYIKDKYHNEITFGDK
SRVGRGGMTAKVKAALVASNSGTPVVITSGSASGSIIRVLQGEKIGTLFHKDASLWEPSSDVSACEMAVTARECSRR
LQNLSSDERNKILLDVADALEKNENLIRTENEADVAAAQDAGYEKFLVDRLTLKPGKISALAKSIRTLARMEDPINQ
ILKRTEIAENLILEKTSCPLGVLLVIFESRPDALVQIASLAIRSGNGLLLKGGKEAMRSNKVITGAIPSNVGEKLIG
LVTSRDEIADLLKLHDVIDLVIPRGSNKLVSQIKTSTKIPVLGHADGICHVYIDKSANMNMAKQIVMDAKIDYPAAC
NAMETLLVHKDLIKAPGLDDLLLSLKTKGVAIFGGPVAHEVLCIPEANSFHHEYSSMACAIEFVDDVQSAIDHIHCY
GSAHTDCIVTTDDKVAETFLRQVDSAAVFHNASTRFSDGARFGLGAEVGISTGRIHARGPVGVEGLLTTRWIMRGSG
QVVNGDNDIAYTHKNLPLP
>Zm00001eb347680
MATADRTRTFMKDVKRVIIKVGTAVVTRGDDGRLAVGRLGCLCEQVKELNVLGYEVILVTSGAVGVGKQRLKYRKLV
NSSFADLQKPQMELDGKACAAVGQSGLMALYDMLFTQLDVSSSQLLVTDSDFENPNFRERLCETVESLLDLKVVPIF
NENDAISTRKAPYEDSSGIFWDNDSLAGLLAIELKADLLVLLSDVDGLYSGPPSEPGSKIIHTYIKDKHYSGITFGD
KSRVGRGGMTAKVKAAFVASNSGTPVVITSGFASQSIVRVLQGEKIGTLFHKDASLWEPSKDVSAREMAVAARECSR
RLQNLSSDERKKILLDIADALEQNEDLIRTENEADVSAAQDAGYQKSLVDRLTLKPEKIASLAKSIRTLANMEDPIN
QILKRTEVAEDLVLEKTSCPLGVLLIVFESRPDALVQIASLAVRSGNGLLLKGGKEAMRSNTVLHKVITGAIPDNVG
QKLIGLVTSRDEIADLLKLDDVIDLVIPRGSNKLVSQIKASTKIPVLGHADGICHVYIDKSADMNMAKRIVMDAKTD

YPAACNAMETLLVHKDLIKAPGLDDILLSLKTEGVAIYGGPVAHEVLCIPKADSFHHEYSSMACTIEFVDDVQSAIN
HIHRYGSAHTDCIITTDDKVAETFLRQVDSAAVFHNASTRFSDGARFGLGAEVGISTGRIHARGPVGVEGLLTTRWI
MRGSGQVVNGDKNVAYTHKNLPLQ

P5CS:
> Zm00001eb367740
MGRGGIGGAAAMAMAMETADPARAFVKDVKRIIIKVGTAVVTGMNGRLAMGRLGSLCEQVKQLNFQGYEVILVTSGA
VGVGRQRLQYRKLIHSSFADLQNPQMNFDGKACAAVGQSVLMAIYDTLFSQLDVTSSQLLVTDRDFKDPSFGDQLRE
TVFSLLDLKVVPLFNENDAISTRRQPYEDSSGIFWDNDSLAALLAAELNADLLIMLSDVEGLYSGPPSDPQSKIIHT
YVNEKHGKLISFGEKSSVGRGGMQAKVSAAANAASKGVPVVIASGFATDSIITVLKGEKIGTLFHNEANLWACSKEA
TAREMAVAARDCSRRLQKLSSEERKQILLDIADALEANEDAIRSENDADVEAAQVAGYEKSLVARMTLKPGKITNLA
RSIRKTADMEDPISHTLKRTEVAKDLVFEKAYCPLGVLLIIFESRPDALVQIASLAIRSGNGLLLKGGKEVMRSNAI
LHKVITGVIPDTVGKKLIGLVTSKEEIADLLALDDVIDLVIPRGSKSLVSQIKATTKIPVLGHADGICHVYIDKSAD
MDMAKRIVLDAKIDYPAACNAMETLLVHKDLNKSEGLDDLLVELEKEGVVIYGGPVAHDKLKVPKVDSFRHEYSSMA
CTVEFVDDVQSAIDHINRYGSAHTDCIITTDRSAAEAFLQQVDSAAVFHNASTRFCDGTRFGLGAEVGISTERIHAR
GPVGVDGLLTTRCILRGSGQVVNGDKGVVYTHKDLPLQ

PDH:
>Zm00001eb022980
MAIASRATKRALSTFAAAKLPEEAVAAAAAGEAIAARSHQAVPLPPAPASSVRPLQFEDTGRLFAGEPTSALLRTLA
ALQALSVGPLVDAATAALRSPAVAGSALGRAAARATAYRHFCAGETADEAAAVVRRLWRGGMGGILDYGIEDAEDGH
ACDRNAAGFVSAVDVAASLPPGSASVCIKITALCPIALLEKASDLLRWQKKHPSFSLPWKTHSFPVLSDSSPLHLTA
SEPAALTAEEERELQLAHSRLLAVCARCAEHGIPLLVDAEYATVQPAIDYFTLVGALAFNDAGAADGGRPIVHGTIQ
AYLRDARDRLEAMVRGAERERVRLGLKVVRGAYLTREARLAAALGVPSPVHGSIQETHDCYNGCAAFLLDRVRRGSA
SVMLATHNVESGQLAAARAQELGIPKGDRNLQFAQLMGMADGLSLSLRNAGFQVSKYLPYGPVEQIIPYLIRRAEEN
RGLLSASSFDRQLLRKELVRRVKAMVAGRE
>Zm00001eb391720
MAIASRATKRALSTFAAAKLPEEAIAATAAVPVPLAPAPASSERALLQFEDTGRLFAGEPTPALLRTLAALQALSVG
PLVDAATAALRSPAVAGSALGRAAARATAYRHFCAGETAGEAAAAVRRLWRGGMGGILDYGIEDAEDGGACDRNAAG
FASAVDVAAKLPPGSASVCIKITALCPIALLEKASDLLRWQKRHPSLNLPWKTHDFPILSDSSPLHLTASEPPALSA
EEERELELAHERVLAVCARCAERGVPLLVDAEYAAVQPAIDYLTLAGALACNAERSIVHGTVQAYLRDARERLETMA
RGVERARVRLGVKLVRGAYLAREARVAAALGVPSPVHGSIRETHDCYNGCAAFLLDRVRRGSASVVLATHNVESGQL
AAARAEELGIPRGDRNLQFAQLMGMADGLSLGLRNAGFQVSKYLPYGPVEQIVPYLIRRAEENRGLLSASSFDRQLL
REELVRRFKAAVLGRE

OAT:
>Zm00001eb050460
MRMEQDCSAHNYHPIPMVFSKGEGSHIVDPEGNKYIDFLSAYSAVNQGHCHPKVLRALIEQAERLTLSSRAFYNDKF
PIFAEYLTSMFGYDMMLPMNTGAEGVETAIKLARKWGYEKKHIPKNEALLVSCCGCFHGRTLGVISMSCDNDATRGF
GPLVPGHLKVDFGDIDGLKKIFEELGDRICGFLFEPIQGEAGVVIPPDGYLKGARDLCSKHNVLMIADEIQTGIART
GKMLACDWENIRPDMVILGKALGAGVVPVSAVLADKDVMLCIRPGEHGSTFGGNPLASAVAVASLKVVRDEGLVERA
AKLGQEFRDQLQKVQQKFPQILREVRGRGLLNAVDLNNDALSPASAYDICIKLKERGILAKPTHDTIIRLAPPLTIS
PEELAEASKALSDVLEHDLPQLQLQKQIKKPESEAEKPVCDRCGRDLYG

Glycine max
P5CR:
>GLYMA_13G296500
MSMVKVEEEEEQQATTPIHTSSPSSYVLLLQIMSKRRTWVCIFVLVYGLLFTSSWNFLKSMISWYKLQADQSSTTSW
WPALYASVLLGAVFGVLWMVAALAVVVPAVLVTWIAIVVLLAFFGKPKRTLVAEGRMITKEIFGFVMKVLLKEGNVV
AAVCAVLGYFVLGRTNGKGVD
>GLYMA_06G292300
MAKEEEQQQQQHPPYAYTLILNIFSKRRTWACLFLVVYGTLLASSWNLLKSTLSWYNLQVESSTTSAWPALYASVL
LGTVFGVLSMVAAMVVMVPAVVVTWITIVVLLAFFGKPKRTLVVEGRKITKEIFGFVMRTLLKEGNFVAAVCAVLGY
FALVRRNGTQGGGVAVVGES
>GLYMA_12G204900

MAKVEEEEQPTSPIHTSPSSYVLLLQIMSKRKTWVCIFVLVYGLLFTSSWNFLKSMVSWYKLRAAESSITSWWPALY
ASVLLGAVFGVLSMVAALAVVVPAVLVTWIALVGLLAFFGKPKRTLVVEGRMITKEIFGFVMQVLLKEGNIVAAVCA
VLGYFVLGRTNGKGNE
>GLYMA_05G041500
MSKIVIIITLLIAVTASCFVCLLRDRKTEGFYLLLALVTIASAMVLAFRATMETFITVLVLLAFAGYRRRVLIQQRW
RISLHVGWCLLDQPYIQIKEGALRSGLCHLFQLVCHLLMNEVNAIKLVSVQGN
>GLYMA_17G084800
MTKISIIITLLIAVTASCFVCVLLDRKIEGFYMVVALVTIIASAMVLAFRATMVTFITVLVLLTFAGFRRRVLIQQR
WRIFLDVAWYLVSVIFRSEKGLLGLACATFISLFATYR
>GLYMA_19G131500
MEIFPIPAESYTLGFIGAGKMAESIARGAVRSGVLPPSRIRTAVHFNLARRGAFESFGVTVLPSNDDVVRESDVVVL
SVKPQLVKDVVSKLTPLLTKNKLLVSVAAGTKLKDLQEWAGNDRFIRVMPNTPAAVGQAASVMSLGGSATEEDGNII
AQLFGSIGKIWKAEEKYFDAITGLSGSGPAYVYLAIEALADGGVAAGLPRDLSLSLASQTVLGAASMVSQTGKHPGQ
LKDDVTSPGGTTITGIHELENGGFRGTLMNAVVAAAKRSRELS
>GLYMA_03G129100
MEIVPIPADSYNLGFIGAGKMAESIARGAVRSGVLPPSRIRTAVHSNPARRDTFESFGVTVLPSNDDVVRESNVVVF
SVKPQLVKDVVFKLTPLLTKNKLLVSVAAGTKLKDLQEWAGNDRFIRVMPNTPAAVGEAASVMSLGGSATEEDGNII
AKLLGSIGKIWKAEEKYFDAITGLSGSGPAYVYLAIEALADGGVAAGLPRDLSLSLASQTVLGAASMVIQTGKHPGQ
LKDDVTSPGGTTITGIHELENGGFRGTLMNAVVAATKRSRELS
>GLYMA_12G113500
MAKEEEQQQPTSPMHPPYAYTIILNIFSKRRTWACLFLLVYGTLLTSSWNFLKSTLSWYNLQVESSTTSAWPALYAS
VLLGTVFGVLSMVAALVVMVPALVVTWITIVVLLAFFGKPKRTLVVEGRKITREIFGFVMRTLLKEGNFVAAVCAVL
GYFALVRRNGTQGGVVVVGES

P5CDH:
>GLYMA_20G086900
MFGEISAKAAHMLSLHKVSDFFTRLIQRISPKSYQQAFGEVYVTQMFLENLCGDQVRFLVRSFAVPGNHVGQRCHGF
RWPYGPVTIITPFNFPLEIPALQLMGALYMANKPVLKVDSKNWSKTSLLSKLEDLAERRKLANLTIDSVLTVSIACL
LFPPSSNCSRI
>GLYMA_05G029200
MFMFLVSRVTKDSISRNRNAFASFAFSRCAHSLSFATVEAEEISGSRPAEVLNLVQGKWVGSSNWNTIADPLNGDSF
IKVAEVDETGIQPFIKSLSSCPKHGVHNPFKAPERYLMYGDISTKAAHMLSLPKVSDFFTKLIQRVSPKSYQQAFGE
VYVTQKFLENFCGDQVRFLARSFGVPGNHLGQQSHGFRWPYGPVAIITPFNFPLEIPVLQLMGALYMGNKPVLKVDS
KVSIVMEQMLRLLHTCGLPAEDVDFINSDGKTMNRLLLEANPRMTLFTGSSRVADKLAVDLKGRVKLEDAGFDWKIL
GPDVHQEDYIAWVCDQDAYACSGQKCSAQSLLFMHENWSKTSLLSKLKDLAERRKLEDLTIGPVLTCTTGMMLEHKN
KLLEIPGSKLLFGGSPLENHSIPPIYGAIKPTAVYVPLEEIMKDKNFDLVTKEIFGPFQVITDYKNSQLSVVLDAVE
RMHNHLTAAVVSNDPLFLQEVVGNSVNGTTYAGLRARTTGAPQNHWFGPAGDARGAGIGTPEAIKLVWSCHREVIYD
FGPVPKDWKTPQST
>GLYMA_17G097800
MPYIVHIGDCCPNGMLLWIYLMFGEISAKAAHMLSLPKVLDFFTRLIQRVSPKSYQQAFGEVYVTQKFLENFCGDQV
RFLARSFAVPGNHLGQQSHGFRWPYGPVAIITPFNFPLEIPVLQLMGALYMGNKPVLKVDSKVSIVMEQMLRLLHTC
GLPLEDVDFINSDGKTMNKLLLEGNPRMTLFTGSSRVAEKLAVDLKGRVKLEDAGFDWKILGPDVHQEDYVAWVCDQ
DAYACSGQKCSAQSLLFMHENWSKTSLLSKLKDLAERRKLADLTIGPVLTVTTDSMLEHVNKLLEIPGSKLLFGGSP
LENHSIPPIYGAIKPTAVYVPLEEIMKDKNFELVTKEIFGPFQVITDYQNSQLAVVLDALERMHNHLTAAVVSNDPL
FLQEVIGKSVNGTTYAGLRARTTGAPQNHWFGPAGDARGAGIGTPEAIKLVWSCHREIIYDFGPVPKNWEVPPST
>GLYMA_05G029100
MGALYMGNKPVLKVDSKVSIVMDQMLRLLHNCGLPLEDVDFINSDGKTMNKLLLEANPRMTLFTGSSRVAEKLAVDL
KGRVKLEDAGFDWKILGPDVLQEDYIAWVCDQDAYACSGQKCSAQSLLFMHENWSKTSLLSKLKDLADRRKLADLTV
GPVLTVTTDSMLEHINKLLEIPGSKLLFGGQPLEDHSIPPIYGAMKPTAVYVPLEEIMKAKNFELVTREIFGPFQIV
TDYKSSQLSVVLDALERMHNHLTAAVVSNDPLFLQEVIGQSVNGTAYAGLRARTTGAPQNHWFGPAGDARGAGIGTP
EAIKLVWSCHREIIYDFGPVPKNWEVPPST
>GLYMA_07G137300
MLAAYLGTEPQTLTTSIHQKNNTRNPLPLCFFYFSLLLLLLHSPSMDPTRAFVKSVKRVVVKVGTAVVTRSDGRLAL
GRLGALCEQLKELNNNDYEVILVTSGAVGLGRQRLRYRRLVNSSFSDLQNPQGDLDGKACAAVGQSSLMALYDLMFS
QLDVTSSQLLVNDGFFRDTAFRKQLSDTVSSLLDLRVIPIFNENDAVSTRKAPYEGKNCLQDSSGIFWDNDSLAGLL
ALELKADLLVLLSDVEGLYSGPPSDPKSKLIHTYVKEKHQSEITFGEKSRLGRGGMTAKVNAAVCAAYAGTPVIITS
GYATDNIIRVLRGERIGTVFHKDAHLWTSIKEVSAHEMAVAARNSSRRLQVLNSEERRKILLAMADALEINESVIRL

ENGADVADAEEMGYEKALISRLTLRPEKISSLVKSVRMLADMEEPIGQILKRTELADKLILEKISCPLGVLLVIFES
RPDALVQIAALAIRSGNGLLLKGGKEAKRSNAILHKVITSIIPDTVGDKLIGLVTSREDIPDLLKLDDVIDLVVPRG
SNKLVSQIKGTTKIPVLGHADGICHVYVDKTANIDMAKKIIRDAKIDYPAACNAMETLLVHVDLSRNGGLDELVAEL
QREGVQLYGGPRASNLLNISETHSFHLEYSSLACTVEIVDDVFAAIDHIHHHGSAHTECIVAEDSEVAEAFLSQVDS
AAVFHNASTRFCDGTRFGLGAEVGISTGRIHARGPVGVEGLLTNRWILRGSGHVVNSDRGVTYTYKDLPVKA
>GLYMA_18G188000
MDPTRAFVKSVKRVVVKVGTAVVTRSDGRLALGRLGALCEQLKELNNNDYEVILVTSGAVGLGRQRLRYRRLVNSSF
SDLQNPQGDLDGKACAAVGQSSLMALYDIMFSQLDVTSSQLLVNDGFFRDTAFRKQLSDTVSSLLDLRVIPIFNEND
AVSTRKAPYEDSSGIFWDNDSLAGLLALELKADLLVLLSDVEGLYSGPPSDPKSKLIHTYVKEKHQREITFGEKSRL
GRGGMTAKVNAAVCAAYAGTPVIITSGYATDNIIRVLRGERIGTVFHKDAHLWTSIKEVSAHEMAVAARNSSRRLQV
LNSEERRKILLAMADALKNNESVIRLENGADVADAEEMGYEKALISRLTLRPEKISSLVKSVRMLAEMEEPIGQILK
RTELADKLILEKISCPLGVLLVIFESRPDALVQIAALAIRSGNGLLLKGGKEAKRSNAILHKVITSIIPDTVGDKLI
GLVTSREHIPDLLKLDDVIDLVVPRGSNKLVSQIKDSTKIPVLGHADGICHVYVDKTANIDMAKKIIRDAKIDYPAA
CNAMETLLVHVDLSRNGGLDELVAELRHEGVQLYGGPRASSLLKISETHSFHLEYSSLACTIEIVDDVFAAIDHIHH
NGSAHTECIVAEDSEIAEAFLSQVDSAAVFHNASTRFCDGARFGLGAEVGISTSRLHARGPVGVEGLLTNRWILRGS
GQVVNGDRGVTYTYKDLPVKA
>GLYMA_18G034300
MENTDPCRHFLKDVKRIIIKVGTAVVTRQDGRLAVGKLGALCEQIKELNSLGYEIILVSSGAVGLGRQRLRYRKLIN
SSFADLQKPQVELDGKACAAVGQNSLMALYDVLFSQLDVTSAQLLVTDNDFRDKDFRMQLSETMKSLLALKVIPIFN
ENDAVSTRKAPYEDSSGIFWDNDSLSALLALELKADLLILLSDVEGLYSGPPSDPRSKLIHTYIKEKHQSEITFGDK
SRVGRGGMTAKVKASIHAAEAGIPVIITSGYAAENIIKVLQGQRIGTLFHKDAHKWAPVKEVDAREMAVAARDCSRR
LQALSSEERKQILLKIADALEAHQNEIRIENEADVADAKEAGYEKSLVARLVLKNEKLASLANNIRIIANMEDPIGR
VLKRTELAEGLILEKTSSSLGVLLIVFESRPDALVQIASLAIRSGNGLLLKGGKEAKRSNAILHKVITEAIPDIVGS
KLIGLVTSRAEIPELLKLDDVIDLVIPRGSNKLVTQIKSSTKIPVLGHADGICHVYVDKSADLEMARRIVLDAKIDY
PAGCNAMETLLVHKDLVEKGWLNSIIIDLRTEGVTLYGGPKASPLLNIPMARMLHHEYNSLACTVEIVDDVYAAIDH
INLYGSAHTDSVVAEDHEVANVFLRQVDSAAVFHNASTRFSDGARFGLGAEVGISTSRIHARGPVGVDGLLTTRWIL
KGSGQIVDGDKAVNYTHRDLSI
>GLYMA_02G251100
MADPSRSFMKDVKRVIIKVGTAVVTREEGRLAVGRLGALCEQIKQLNSLGYDIILVSSGAVGIGRQRLRYRKLINSS
FADLQKPQHELDGKACAAVGQNSLMALYDTLFTQLDVTSAQLLVTDNDFRDKDFRKQLTETVKSLLSLKVIPVFNEN
DAVSTRKAPYEDSSGIFWDNDSLSALLALELKADLLVLLSDVEGLYSGPPSDPHSKLIHTYIKEKHQNEITFGDKSR
VGRGGMTAKVKAAVHAADAGIPVVITSGFAAENIINVLQGQRIGTLFHKDAHEWVQVKEVDAREMAVAARECSRRLQ
AISSEERNQILHKIADALEANEKIIRTENEADIAVAQEAGYEKSLVARLAIKPGKIASLANNMRIIANMEDPIGQVL
KRTELSDGLILEKTSSPLGVLLIVFESRPDALVQIASLAIRSGNGLLLKGGKEARRSNAILHKVITEAIPDTVGGKL
IGLVTSREEIPELLKLDDVIDLVIPRGSNKLVSQIKSSTKIPVLGHADGVCHVYVDKSANVEMARRIVLDAKIDYPA
ACNAMETLLVHKDLIEKGWLNDIVVDLRTEGVKLYGGPRASSLLNIPQAQTFHHEYSSLACTVEIVDDVYAAIDHIN
LYGSAHTDSIVAEDKEVANVFLRQVDSAAVFHNASTRFSDGARF
GLGAEVGISTSRIHARGPVGVEGLLTTRWILKGSGQVVDGDKGIVYTHKDIAT
>GLYMA_01G099800
MELLQNGHKNFVSIKPSELPLTNGAALTLLNSLSKTQYLGNIDPSRVFVTKVKRIIVKVGTAVVTRSDGRLALGRIG
ALCEQLKELSSQGYEVILVTSGAVGLGRQRLRYRKLANSSFSDLQKPQEELDGKACAAVGQSSLMALYDTMFSQLDV
TSSQLLVNDGFFRDSGFRKQLSDTVNSLLDLRVIPIFNENDAVSTRKAPYEDSSGIFWDNDSLAGLLALELKADLLV
LLSDVEGLYSGPPSDPNSRLIHTYIKEKHQGEITFGDKSRLGRGGMTAKVNAAVCAAHAGIPVIITSGYATNNIIRV
LQGERIGTVFHKDAHLWTNIKEVSAREMAVAAREGSRRLQILKSEERRKILLAIADALETSESMIRHENEADVADAV
ATGYEKSLMSRLILKQEKISSLAKSVRMLADMEEPIGQILKRTELVDKLILEKISCPLGVLLVIFESRPDALVQIAA
LAIRSGNGLLLKGGKEARRSNAILHKVITSVMPDTVGDKLIGLVTSRDEILDLLKLDDVIDLVVPRGSNKLVSQIKE
STKIPVLGHADGICHVYVDKSANIDMAKQIVRDAKTDYPAACNAMETLLVHKDLSNNGGLHELVLELQREGVKMFGG
PRASGLLNIAETNTFHHEYSSLACTVEIVEDVFAAIDHINQHGSAHTECIVTEDSEVAETFLSQVDSAAVFHNASTR
FCDGARFGLGAEVGISTSRIHARGPVGVEGLLTNRWILRGSGHVVDGDQGINYTYKELPLKA
>GLYMA_03G069400
MELLQNGHKNLVSIKPSELPLLNGAALTLLNSLSETHEYYGNIDPSRVFVTKVKRIIVKVGTAVVTRSDGRLALGRI
GALCEQLKELSSQGYEVILVTSGAVGLGRQRLRYRKLANSSFSDLQKPQGELDGKACAAVGQSSLMALYDTMFSQLD
VTSSQLLVNDGFFRDSGFRKQLSDTVNSLLDLRVIPIFNENDAVSTRKAPYEDSSGIFWDNDSLAGLLALELKADLL
VLLSDVEGLYSGPPSDPNSKLIHTYVKEKHQGEITFGDKSRLGRGGMTAKVNAAVCAAHAGIPVIITSGYATNNIIR
VLQGERIGTVFHKDAHLWTNIKEMSAREMAVAAREGSRQLQILKSEDRRKILLAIADALEKNESMIRHENEADVADA
VVAGYEKSLISRLTLKQEKISSLAKSVRLLADMEEPIGQILKRTELVDKLILEKTSCPLGVLLVIFESRPDALVQIA
ALAIRSGNGLLLKGGKEARRSNAILHKVITSVMPDTVGDKLIGLVTSRDEIPDLLKLDDVIDLVVPRGSNKLVSQIK

ESTKIPVLGHADGICHVYVDKSANFDMAKQIVRDAKTDYPAACNAMETLLIHKDLSNNGGLNELVLELQREGVKMFG
GPRASGLLNIAETNTFHHEYSSLACTVEIVEDVFAAIDHINQHGSAHTECIVTEDSEVAETFLSQVDSAAVFHNAST
RFCDGARFGLGAEVGISTSRIHARGPV
GVEGLLTNRWILRGSGHVVDGDQGIDYTYKELPLKA

P5CS:
>GLYMA_14G06560
MADRSRSFMKDVKRVVIKVGTAVVTREEGRLAVGRLGALCEQIKQLNSLGYDIILVSSGAVGIGRQRLRYRKLINSS
FADLQKPQLELDGKACAAVGQNSLMALYDILFTQLDVTSAQLLVTDNDFRDEDFRKQLTETVKSLLSLKVIPVFNEN
DAVSTRKAPYEDSSGIFWDNDSLSALLALELKADLLVLLSDVEGLYSGPPSDPHSKLIHTYIKEKHQNEITFGDKSR
VGRGGMTAKVKAAVHAADAGIPVVITSGFAAENIINVLQGQRIGTLFHKDAHEWVQVKEVDAREMAVAARECSRRLQ
AISSEERKQILLKIADDLEANEKIIRTENEADVAVAQQAGYENSLVARLALKPGKIASLANNVRIIANMEDPIGQVL
KRTELSDGLILEKTSSPLGVLLIVFESRPDALVQIASLAIRSGNGLLLKGGKEAKRSNAILHKVITEAIPDTVGGKL
IGLVTSREEIPELLKLDDVIDLVIPRGSNKLVSQIKSSTKIPVLGHADGVCHVYVDKSANVEMARGIVLDAKLDYPA
ACNAMETLLIHKDLIEKGWLNDIVVDLRTEGVKLYGGPRASSLLNIPQAHSFHHEYSSLACTVEIVDDVYAAIEHIN
LYGSAHTDSIIAEDKEVANVFLRQVDSAAVFHNASTRFSDGARFGLGAEVGISTSRIHARGPVGVEGLLTTRWILKG
SGQVVDGDKGIVYTHKDLAA
>GLYMA_13G049700
MATRVIPPRILKNLRYNTTTKPLNAAQPSISPAIASPSLFERSPSPPAADVIPASAAGTAALNLDDAERLFASVSTK
RLLRSSAVLHATAVGPMVDLGMWMMKSRVFQSGVLKDLVMAATKETFFSHFCAGEDAASAGRSIRALNDAGLRGMLG
YGVEDAHENDGCDRNLNGFLHTVDVSKSLPPSSVSFVIVKITAICPMALLERMSDLLRWQQKDPSFVLPWKQDSLPI
FAESSPLYHTQKRPEPLTPEEESDLQLANQRLLELCQKCEEANMPLLVDAEHTTVQPAIDYFTYSSSIRHNKDDNPI
VFGTIQTYLKDAKERLLLTTKAAEKMGVPMGFKLVRGAYMSTESKLAEFFGYASPIHNTIQDTHNCFNDCSSFLLEK
IANGPGSVVLATHNIESGKLAATKAYELGVGKVNHKLEFAQLYGMSEALSFGLSNAGFQVSKYMPFGPVDMVMPYLL
RRAEENRGLLAASGFDRQLMRKELGRRLKAAVF
>GLYMA_18G278900
MATRVIPPRILRNLRYNTATKPLNSSHPPLSPSLSPSLCIPAPPPISAVLPPSDDLSFRDVEKLFSSVPTTTLLRST
AVLHATALEPMVDFGTWLMRSNLMQVPGLSDLILATVRNTFFDHFCAGEDATTTADSVRHLNKAGLRGMLVYGVEDA
NNNDACHRNFKGFLHTIDVSRSLPPSSVSFVIVKITAICPMSLLERMSDLLRWQHKDPSFSLPWKQDCFPIFSESSP
LYHTSKRPEPLTREEESDLQLAMQRFLELCQKCVQANIPLLVDAEHTSVQPAIDYFTYSSAILHNKGDNPIVFGTIQ
TYLKDAKERLVLAAEAADNMGIPMGFKLVRGAYMSSETKLAESLGYSSPIHNTIEDTHKCFNDCSSFMLEKVANGPG
GVVLATHNVESGKLAAAKAHELGVGKVNHKLEFAQLHGMSEALSFGLSNAGFQVSKYMPFGPVETVMPYLLRRAEEN
RGMLAASGFDRQLMRKELGRRLKAAFF
>GLYMA_19G043000
MATRVIPPRILKKLRYNTTTKPLNAAHPSISPVIAPPSLFERSPSPVADAVSTTSTTMETANLNLDDAERLFASVST
EKLLRSSAVLHATAVGPMVDLGMWLMKSPVFQTGLPKDLIMAATKETFFSHFCAGEDAAAAGRSISALKEAGLRGML
VYGVEDAHENDGCDRNLKGFLHTVDVSKSLPPSSVSFVIVKITAICPMTLLERMSDLLRWQQKDPSFVLPWKQDSLP
IFAESSPLYHTQKRPEPLTPEEESDLQLANQRLLELCQRCEEANMPLLVDAEHTTVQPAIDYFTYSSSIRHNKDDNP
IVFGTIQTYLKDAKERLLLTTKAAEKMGVPLGFKLVRGAYMSTESKLAESFGYASPIHNTIQETHNCFNGCSSFLLE
KIANGPGSISVVLATHNIESGKLAAAKAYELGVGKVNHKLEFAQLYGMSEALSFGLSNAGFQVSKYMPFGPVDMVMP
YLLRRAEENRGLLAASGFDRQLMRKELGRRLKAAMF
>GLYMA_19G042900
MATRVIPPRMILKNIRYNTATKPLKTTHPSLSPVTATASLVKKPSSPATDAWASFAQASVTTETAALNLEDAEQLFA
SVSTRKLLQSSAVMHATAVGPVVDLGMRVMKSRVFQSGVLRNLLMAATKETFYAQFCAGEDAATAGRSISALNEVGL
RGMLVYGVEDAHENDGCDRNLKGFLHTVDVSKSLPPSSVSFVIVKITAICPMALLERMSDLLRWQQRDPSFVLPWKQ
DSLPIFAESSPLYHTQKRPEPLTPEEESDLQLANQRLLELCQRCEEANMPLLVDAEHTTVQPAIDYFTYSSAIRHNK
DDNPIVFGTIQTYLKDAKERLLLATKAAEKMGVPMGFKLVRGAYMSIESKLAESFGYASPVHNTIQDTHNCFNDCSS
FMLEKIANGIGSVVLATHNIESGKLAVAKAHELGVGKVNHKLEFAQLYGMSEALSFGLNNEGFQVSKYMPFGPVDMV
MPYLLRRAEENRGLLAASGFDRQLMRKELARRLKAAVF
>GLYMA_08G255900
MATRVIPPRILRKLRYNTATKPLNSSHPPLSPSLSPSPCLLPAPLPSPAVLRPSAAASDDLSFRDVEKLFSSVSTTS
LLRSSAVLHATAVEPMVDFGTWLLRSNLMHVHGIRDLILATVRNTFFDHFCAGEDAITTAASIRHLNRAGLRGMLVY
GVEDANDNDACHRNFKGFLHTIDVSRSLPPSSVSFVIVKITAICPMSLLERMSDLLRWQHKDPCFSLPWKQDCFPIF
SESSPLYHTSNKPEPLTPEEEKDLQLAIQRFHELCHKCVQVNIPLLVDAEHTSVQPAIDYFTYSSAILHNKGDNPIV
FGTMQTYLKDAKERLLLAAEAADNMGIPMGFKLVRGAYMSSETKLAESLGYSSPIHDTIEDTHKCFNDCSSFMLEKV
ANGPGGLVLATHNVESGKLAAAKAHELGIGKVNHKLEFAQLHGMSEALSFGLSNAGFQVSKYMPFGPVETVMPYLLR
RAEENRGMLAASGFDRQLMRKELGRRLKAAVF

PDH: No gene found in the ensemble plants database

OAT:
>GLYMA_19G189900
MGEELAGQLLKIQQQYPNYVKEVQGRGLFIGVEFNSKNLFPVSGYELCKKLKYKGVLAKPTHDTFICFTPPLHNENK
KQCLYIKLSLYYVKVLPLENY
>GLYMA_08G245800
MHNCFNCLFTQILGKALGGGVIPVSAVLANKDVMLCIQPGQHGSTFGGNPLASAVAIASLEVIKIERLVERSAQMGE
ELAGQLLKIQQQYPDYVKEVRGRGLFIGVEFNSKNLFPVSGYEVCKKLKYRGVLAKPTHDTIIRFTPPLCIR
>GLYMA_17G184700
MLACEWEEVRPDIVILGKALGGGVIPVSAVLADKDVMLCIQPGQHGRSAQMGEELVGQLLKIQQQYPDYVKEVRGRG
LFIGVEFNSKNLFPVSGCELCKKLKYRGVLAKPTHDTIIRFTPPLCIRRENLHYHPRLY
>GLYMA_15G181100
MHNCFNCLFTQILGKASGGGVIPISAVLADKDVMLCIQPGQHGSTFGGNPMASAIAIGSVEVIKNERLVERYQMHCQ
ILYCQYILITMQT
>GLYMA_02G189300
MHNCFNCLFTQILGKASGGGVIPVSAVLADKDVMLCIQPGQHGSTFGGNPMASAVAIGSVEVIKNERLVERYQMHCK
ILYCQYILIIMQT
>GLYMA_05G141900
MVGICFFFGCQLICYHPLPIVFSQAKGTSVWDPEGNKYLDFLSGYSAVNQGHCHPKILKALQEQAQRLTVSSRAFYN
DRFPVFAEYVTNMFGYDMVLPMNTGAEGVETALKLARKWGYEKKRIPKDEAIIVSCCGCFHGRTLGVISLSCDNEAT
RGFGPLLPGNLKVDFGDAEALERIFKEKGEHIAAFILEPIQGEAGVIFPPDGYLKAVRDICSKYNVLMIADEIQTGL
ARTGKMLACEWEEVRPDIVILGKALGGGVIPVSAVLADKDVMLCIQPGQHGSTFGGNPLASAVAIASLEVIKNERLV
ERSAQMGEELAGQLLKIQQQYPDYVKEVRGRGLFIGVEFNSKNLFPVSGYELCKKLKYRGVLAKPTHDTIIRFTPPL
CISLDEIQQGSKVLADVLEIDLPKLQKTKPKDAAPVASSACDRCGRVLY
>GLYMA_08G097800
MAATRPVQCLLRRVCRGSRTIGVATEVNASSSSQKIIDKEYEHSAHNYHPLPIVFAQAKGTSVWDPEGNKYLDFLSG
YSAVNQGHCHPKILKALQEQAERLTVSSRAFYNDRFPDFAEYVTNMFGYDMVLPMNTGAEGVETALKLARKWGYEKK
RIPKDEAIIVSCCGCFHGRTLGVISLSCDNEATRGFGPLLPGNLKVDFGDAEALEQIFKEKGEHIAAFILEPVQGEA
GVIFPPDGYLKAVRDLCSKYNVLMIADEIQTGLARTGKMLACEWEEVRPDVLILGKALGGGVIPVSAVLADKDVMLC
IQPGQHGSTFGGNPMASAVAIASLEVIKNERLVERSAQMGEELTGQLLKIQQQYPDYVKEVRGRGLFIGVEFNSKKL
FPVSGYELCKKLKYRGVLAKPTHDAIIRFTPPLCISVDEIQQGSKALADVLEIDLPKLQKTKPIDAAPVATSACDRC
GRVLY

Solanum tuberosum
P5CR:
>PGSC0003DMG400010441
MAESIARGVVKSGILPASRIRTAHSGSARRTAFESIGVTVFDNNSQVVEDSDVIIFSVKPQVVKNVVSQLKPILSEK
QLLVSVAAGVKLKDLQEWAGQGRFIRVMPNTPSAVGEAATVITLGEKATTEDGELISQLFGAIGKVWKADEKLFDAV
TGLRHVPSLIGSGPAYVFLAIEALADGGVAAGLPRELALGLASQTVLGAASMVSGMGKHPGQLKDDVASPGGTTIAG
IHELEKSGFRGILMNAVVAAAKRSKELS
>PGSC0003DMG400018339
MEQQQETQIEETESIISSVFFIKIMSKRRTWVFLFISVYAILLSISWNFLKSVLSWYESTISADSSNSTISISSSGW
PALYASVLLGVAFGVLSMVAALAVVLPATLVTWITILVLLTFAGKPRRDLVLEGKKLTLDIIGFVIKILIKEGNVVA
ALCAVLAYFVLVISRNKQLQNGLDDH
>PGSC0003DMG400027074
MKVTITTKYFLILILILILISFLDLVPFLVISTVVLVGTTILIVLAVRTTVITWIMVLVLLAFTGKRRRGVVKDGKSIT
SEVAMYAANVVFKERGLFAFTGTAIILGFTSMALYC
>PGSC0003DMG400018121
MNISEQSFFNKTTATTTTTTCLFFLLISFSTTTIIFSSGDREAASVFPFIFAAAVVVAGFVVLAVRTTIVAWITVVV
LLAFVGKRRRIFAKDGKKITSEVVVYVVNEVIKEKGFVAISGVMILGLIATALL
>PGSC0003DMG400013145
MAETKQQQEEPPLFPPSSTSSSSFMLLLKNLMSKRRTWVFLFLTVYTILLSISWNFLNSVLSWYESTMKLTPTSALY
GSMILGLAFGVLSIVAALIVVVPATLVTWITILVLLTFAGKGRRDLVMEGKKLTAEITGFVVRVLIREGNLVAVICA
VLGYFALVRRNKEDGIDY

P5CDH:
>PGSC0003DMG400033072
MYRLSAYRQLKNRASSSHLNWITLFNSTRSNHTLSFATVKAEEVSGSQPAEVHNLVQGKWTKSSSWNTILDPLNGQP
FIKVAEVNESELQPFVESLSKCPKHGLHNPFKAPERYLMLGDVSTKAAHALGLPEVSDFFAKLIQRVSPKSYQQALI
EVLVTQKFLENFCGDQVRFLARSFAVPGNHLGQQSHGFRWPYGPVAVIAPFNFPLEIPLLQLMGALYMGNKPVLKVD
SKVCIVMEQMLRLLHECGLPVDDVDFINSDGKTMNKLLVEAKPRMTLFTGSSRVAEKLADDLSGRVKLEDAGFDWKI
LGPDVNEVDYVAWVCDQDAYACSGQKCSAESILFMHENWSKSSLIDKMTELAARRKLDDLTIGPVLTVTTETMLDHA
KKLLQIPGSRLLFGGEALQNHSIPKIYGAIKPTAIFVPLEEILKDEHYPLVTKEIFGPFQVVTEYKDNQLPLVLDAL
EKMHAHLTAAVVSNDILFLQKVIGNSVNGTTYAGLRARTTGAPQNHWFGPAGDPRGAGIGTPEAIKLVWSCHREIIY
DVGPMPLGWKVPAST

P5CS:
>PGSC0003DMG402026767
MDSADPARAFVKDVKRIIIKVGTAVVTRGDGRLALGRMGSLCEQIRELTSQGFEVILVTSGAVGVGRQRLRYRKLIN
SSFADLQKPQGDLDGKACAAVGQNGLMALYDTLFSQLDVTSAQLMVTDNDFRDPDFRRQLNETVNSLLCLKVVPIFN
ENDAISTRKAPYEDSSGIFWDNDSLAALLAMELKADLLVLLSDVEGLYTGPPSDPQSELIHTYVKEKHEGLITFGDK
SRVGRGGMTAKVKAAVYAAYAGIPVVITSGFANNNIIKALDGQRVGTLFHREAIKWASIGDFDAREMAVSARECARR
LQTLSSQERSKILLDIADALEAKEEEILAENEADVAAAQQSGYENSLISRLAMKPGKISSLANSVRVLANMDEPVGR
ILKRTELADGIILEKTSSPLGVLLIIFESRPDALVQIASLAVRSGNGLLLKGGKEAKRSNAILHKVITSSIPPTVGE
RLIGLVTSREEIPELLKLDDVIDLVIPRGSNKLVSQIKAATKIPVLGHADGICHVFIDKSADLDMAKRIVLDAKTDY
PAACNAMETLLVHEDLVQTGGLNDLILELQVKGVSLFGGPKASSVLSIPEANSFHHEYGALACTVEIVEDVNTAIEH
IHRHGSAHTDSIITEDKEVAELFLRQVDSAAVLHNASTRFSDGFRFGLGAEVGISTSRIHARGPVGVEGLLTTRWLA
RGSGQVVDGDKEIVYTHRDLNLEA

PDH:
>PGSC0003DMG400010050
MANKVVCPKVFRDLRRFARCLNTAPVVPPMNFTGNYGSTNVTTPTLQPTDQILADPEKKVINFDDVKELFTGVSTSK
LIRSSLTLQMASIESMVDLGVWVMNSKFMHMPIFKEVILGFVKSTFYEHFCAGKDLIEVRKTVSKLSNVGLKGMLDY
GVEHATENESCDQSMKVFIQTAELAKSLPSSSVSFVVVKITAICTPKLLKRMSDLLRWEQKDPSLNLPWKQKTLPLF
AKSSPFYHTLKRPEPLTVDEERDLQLAHDRLEKICKKCLELDVELLIDAEDAAIQPAIDYFTYSAAIKYHKDDHPLI
FGTIQAYLKDSKERMIIAKKAAEKIGVPMGFKLVRGAYMSSESELASSLGFKSPIHDSIQQTHNCYNSCAEFMLDEI
ANGSGAVVLATHNIESGKLAASKAIDLGIRKDSKKLQFAQLYGMAEGLSFGLRNAGFEVSKYLPFGPVEQVMPYLIR
RAEENRGLLSTSAFDRQLMRKELIRRFDVGTS

OAT:
>PGSC0003DMG400029872
MLPHNICLTLQVIIPPEGYLKAVRDLCSKYNILMIADEIQSGLARSGRLLACDWEEVRPDVVILGKALGGGVLPVSA
VLADKDVMLCIQAGEHGSTFGGNPLASAVAIASLDVIRDEGLAERSAQMGEQLRHQLIKIQRQFPHFIKEVRGKGLF
NAVELNSKSLLPVTAYDICMKLKERGILAKPTHDSIIRLTPPLSMSLEELQEGSNALHDVLVHDLPKMQKEKPARVS
HATSNVCDRCGRDLYGSS

Triticum aestivum
P5CR:
>TraesCS3D02G483400.1
MAAAPPQPAAPAPPAPASGGDAFRLGFVGAGNLAESIARGVAASGVLPASAVRTAPHRRPERAAAFASLGATILASN
AQVVDDSDVIVISVKPQIVKQVLVELKPLLSEEKLLVSIAAGIKMKDLQDWSGQRRIIRVMPNTPSAVGQAASVMCL
GETATENDENRVKSLFSAIGKVWTAEEKYFDAVTGLSGSGPAYIFLAIEAMADGGVAAGLPRDLALGLAAQTVLGAA
TMVSETGKHPGQLKDQVTSPAGTTIAGVHELEKGSFRGTLINAVVAATTRCRELSKN
>TraesCS3B02G538100.1
MAAAPPQPAAPAPGPANGGDAFRLGFVGAGNLAESIARGVAASGVLPASAVRTAPHRRPERGAAFASLGATILASNA
QVVDGSDVIVISVKPQIVKQVLVELKPLLSEEKLLVSIAAGIKMKDLQDWSGQRRIIRVMPNTPSAVGQAASVMCLG
ETATEKDENRVKSLFSAIGKVWTAEEKYFDAVTGLSGSGPAYIFLAIEAMADGGVAAGLPRDLALGLAAQTVLGAAT
MVSETGKHPGQLKDQVTSPAGTTIAGVHELEKGSFRGTLINAVVAATTRCRELSKN
>TraesCS5B02G239000.1
MADQEAAEPGPPSWPPWTSLLLRAMSKRRTWAALFLAVYAALLSSSWSLLASVRVWYYAAAASAGAAAAWPAALYAS
VMYGAVFGLLSMGAALAVAAPAMLVTWITVLVLLAFAGKPRRSLVAEARRATADIARLALRVLLCEGNAVAAVCAVA
SFAALLFGRRDDAERRLI

>TraesCS5A02G240900.1
MADQEAAEPGPPSWPPWTSLLLRAMSRRRTWAALFLAVYAALLSSSWSLLASVRAWYYAAAAGSAAHPAAWPAALYA
SVMYGAVFGLLSMGAALAVAAPAMLVTWITVLVLLAFAGKPRRSLVAEARRATADIARLALRVLLCEGNAVAAVCAA
ASFAALLFGRRDDAGADAGRRLI
>TraesCS5D02G247400.1
MADQEAAEPGPPSWPPWTSLLLRAMSKRRTWAALFLAVYAALLSSSWSLLASVRAWYYAAAAGSAAHPAAWPAALYA
SVMYGAVFGLLSMGAALAVAAPAMLVTWITVLVLLAFAGKPRRSLVAEARRATADIARLALRVLLCEGNAVAAVCAA
ASFAALLFGRRDDADAERRLI

P5CDH:
>TraesCS7D02G493800.1
MAIYDTLFSQLDVTLSQLLVIDRDFRDPSFGLQLHETEFCSRLTVKILFVKYGDPYSGISEASLCLLSHQQAVIQSL
VHQRPSTRTMLTAKESSFPTADSRNRSCAHQG
>TraesCS1D02G160400.1
MAIYDTLFSQLDVTLSQLLVIDRDFRDPSFGLRLHETEFCSRLTVKILFVKYGDPYSGISEASLCLLSHQQAVIQSL
VHQRPSTRTMLTAKESSFPTADSRNRSCAHQG
>TraesCS1A02G357900.2
MSRLVSRRHLAAATALRRSPAAFASRWLHTPAFATVSPEEISGSSPAEVQNFVQGKWIKSANWNWIVDPLNGEKFIK
VGEVQGSEIKPFVESLSKCPKHGLHNPLRAPERYLMYGDISTKAAHMLGQPEVLDFFAKLVQRVSPKSYQQALLEVQ
VSQKFLENFCGDQVRFLARSFAVPGNHVGQMSNGYRWPFGPVAIITPFNFPLEIPLLQLMGALYMGNKPVLKVDSKV
SIVMEQMIRLLHECGLPAEDVDFINSDGITMNKLLLEANPKMTLFTGSSRVAEKLAADLQGRIKLEDAGFDWKILGP
DVQEVDYISWVCDQDAYACSGQKCSAQSMLFMHKNWSSSGLLEKMKKLSERRKLEDLTIGPVLTVTTATMIEHMNNL
LKIPGSEVLFGGEPLENHSIPEIYGAFKPTAVFVPLVEILKNGNFELVTKEIFGPFQVVTEYSEEQLDLVLEACERM
NAHLTAAVVSNDKLFLQAILGRSVNGTTYAGIRARTTGAPQNHWFGPAGDPRGAGIGTPEAIKLVWSCHREIIYDIG
PLPKKWALPAAT
>TraesCS1A02G357900.1
MSRLVSRRHLAAATALRRSPAAFASRWLHTPAFATVSPEEISGSSPAEVQNFVQGKWIKSANWNWIVDPLNGEKFIK
VGEVQGSEIKPFVESLSKCPKHGLHNPLRAPERYLMYGDISTKAAHMLGQPEVLDFFAKLVQRVSPKSYQQALLEVQ
VSQKFLENFCGDQVRFLARSFAVPGNHVGQMSNGYRWPFGPVAIITPFNFPLEIPLLQLMGALYMGNKPVLKVDSKV
SIVMEQMIRLLHECGLPAEDVDFINSDGITMNKLLLEANPKMTLFTGSSRVAEKLAADLQGRIKLEDAGFDWKILGP
DVQEVDYISWVCDQDAYACSGQKCSAQSMLFMHKNWSSSGLLEKMKKLSERRKLEDLTIGPVLTVTTATMIEHMNNL
LKIPGSEVLFGGEPLENHSIPEIYGAFKPTAVFVPLVEILKNGNFELVTKEIFGPFQVVTEYSEEQLDLVLEACERM
NAHLTAAVVSNDKLFLQEVLGRSVNGTTYAGIRARTTGAPQNHWFGPAGDPRGAGIGTPEAIKLVWSCHREIIYDIG
PLPKKWALPAAT
>TraesCS1B02G374500.1
MSRLVSRRHLAAAAALRRSPAAFASRWLHTPAFATVSPEEISGSSPAEVQNFVQGKWIKSANWNWIVDPLNGEKFIK
VAEVQGSEIKPFVESLSKCPKHGLHNPLRAPERYLMYGDISTKAAHMLGQPEVLDFFAKLVQRVSPKSYQQALLEVQ
VSQKFLENFCGDQVRFLARSFAVPGNHVGQMSNGYRWPFGPVAIITPFNFPLEIPLLQLMGALYMGNKPVLKVDSKV
SIVMEQMIRLLHECGLPAEDVDFINSDGITMNKLLLEANPKMTLFTGSSRVAEKLAADLQGRIKLEDAGFDWKILGP
DVQEVDYISWVCDQDAYACSGQKCSAQSMLFMHKNWSSSGLLEKMKKLSERRKLEDLTIGPVLTVTTATMIEHMNNL
LKIPGSKVLFGGEPLENHSIPEIYGAFKPTAVFVPLVEILKNGNFELVTKEIFGPFQVVTEYSEEQLDLVLEACERM
NAHLTAAVVSNDKLFLQEVLGRSVNGTTYAGIRARTTGAPQNHWFGPAGDPRGAGIGTPEAIKLVWSCHREIIYDIG
PLPKKWALPAAT
>TraesCS1B02G374500.2
MSRLVSRRHLAAAAALRRSPAAFASRWLHTPAFATVSPEEISGSSPAEVQNFVQGKWIKSANWNWIVDPLNGEKFIK
VAEVQGSEIKPFVESLSKCPKHGLHNPLRAPERYLMYGDISTKAAHMLGQPEVLDFFAKLVQRVSPKSYQQALLEVQ
VSQKFLENFCGDQVRFLARSFAVPGNHVGQMSNGYRWPFGPVAIITPFNFPLEIPLLQLMGALYMGNKPVLKVDSKV
SIVMEQMIRLLHECGLPAEDVDFINSDGITMNKLLLEANPKMTLFTGSSRVAEKLAADLQGRIKLEDAGFDWKILGP
DVQEVDYISWVCDQDAYACSGQKCSAQSMLFMHKNWSSSGLLEKMKKLSERRKLEDLTIGPVLTVTTATMIEHMNNL
LKIPGSKVLFGGEPLENHSIPEIYGAFKPTAVFVPLVEILKNGNFELVTKEIFGPFQVVTEYSEEQLDLVLEACERM
NAHLTAAVVSNDKLFLQAILGRSVNGTTYAGIRARTTGAPQNHWFGPAGDPRGAGIGTPEAIKLVWSCHREIIYDIG
PLPKKWALPAAT
P5CS:
>TraesCS1A02G281400.1
MAGADPNRSFMKDVKRIIIKVGTAVITRNDGRLALGRIGALCEQVKDLNAQGYEVIMVTSGAVGVGRQRLRYRKLVN
SSFADLQKPQMELDGKACAAVGQSGLMALYDMLFTQLDVSSSQLLVTDSDFDNSNFRERLRETVESLLELRVIPIFN
ENDAISTRKAPYEDSSGIFWDNDSLAGLLALELKADLLVLLSDVDGLYSGPPSEPSSKLIHTYIKEKHYHEITFGDK

SRVGRGGMTAKVQAAVWASTGGVPVVITSGCASQSLVKVLRGEKIGTLFHKNASLWEPSKDTSVREMAVAARDCSRH
LQNLSSEERKKILLDVADALEANEDLIRSENEADLAAAHEAGYESALVSRLTLKPGKIASLAKSVRTLANMEDPINE
ILKRTEVADGLVLEKTSCPLGVLLIIFESRPDALVQIASLAIRSGNGLLLKGGKEAMRSNAILHKVITNAIPDNVGE
KLIGLITTRDEIADLLKHDDVIDLVIPRGSNKLVAQIKSSTKIPVLGHADGVCHVYIDKSADMDMAKRIVMDAKIDY
PAACNAMETLLVHKDLMKIPELNDILVALKTAGVNLYCGPVAHKVLGYPKADSLHLEYSSMACTVEIVDDVQSAIDH
IHRYGSAHTDCVVTTDDKVAETFLRQVDSAAVLYNASTRFSDGARFGLGAEVGISTGRIHARGPVGVEGLLTTRWLL
RGKGQVVNGDKDVEYTHKSLPLQ
>TraesCS1A02G281400.3
MAGADPNRSFMKDVKRIIIKVGTAVITRNDGRLALGRIGALCEQVKDLNAQGYEVIMVTSGAVGVGRQRLRYRKLVN
SSFADLQKPQMELDGKACAAVGQSGLMALYDMLFTQLDVSSSQLLVTDSDFDNSNFRERLRETVESLLELRVIPIFN
ENDAISTRKAPYEDSSGIFWDNDSLAGLLALELKADLLVLLSDVDGLYSGPPSEPSSKLIHTYIKEKHYHEITFGDK
SRVGRGGMTAKVQAAVWASTGGVPVVITSGCASQSLVKVLRGEKIGTLFHKNASLWEPSKDTSVREMAVAARDCSRH
LQNLSSEERKKILLDVADALEANEDLIRSENEADLAAAHEAGYESALVSRLTLKPGKIASLAKSVRTLANMEDPINE
ILKRTEVADGLVLEKTSCPLGVLLIIFESRPDALVQIASLAIRSGNGLLLKGGKEAMRSNAILHKVITNAIPDNVGE
KLIGLITTRDEIADLLKMVFVMYILTNQQTWIWQNVL
>TraesCS1A02G281400.2
MAGADPNRSFMKDVKRIIIKVGTAVITRNDGRLALGRIGALCEQVKDLNAQGYEVIMVTSGAVGVGRQRLRYRKLVN
SSFADLQKPQMELDGKACAAVGQSGLMALYDMLFTQLDVSSSQLLVTDSDFDNSNFRERLRETVESLLELRVIPIFN
ENDAISTRKAPYEDSSGIFWDNDSLAGLLALELKADLLVLLSDVDGLYSGPPSEPSSKLIHTYIKEKHYHEITFGDK
SRVGRGGMTAKVQAAVWASTGGVPVVITSGCASQSLVKVLRGEKIGTLFHKNASLWEPSKDTSVREMAVAARDCSRH
LQNLSSEERKKILLDVADALEANEDLIRSENEADLAAAHEAGYESALVSRLTLKPGKIASLAKSVRTLANMEDPINE
ILKRTEVADGLVLEKTSCPLGVLLIIFESRPDALVQIASLAIRSGNGLLLKGGKEAMRSNAILHKVITNAIPDNVGE
KLIGLITTRDEIADLLKHDDVIDLVIPRGSNKLVAQIKSSTKIPVLGHADGVCHVYIDKSADMDMAKRIVMDAKIDY
PAACNAMTRTKP
>TraesCS1B02G290600.2
MAGADPNRSFMKDVKRIIIKVGTAVITRNDGRLALGRIGALCEQVKDLNAQGYEVIMVTSGAVGVGRQRLRYRKLVN
SSFADLQKPQMELDGKACAAVGQSGLMALYDMLFTQLDVSSSQLLVTDSDFDNSNFRERLRETVESLLELRVIPIFN
ENDAISTRKAPYEDSSGIFWDNDSLAGLLALELKADLLVLLSDVDGLYSGPPSEPSSKLIHTYIKEKHYHEITFGDK
SRVGRGGMTAKVQAAVWASTGGVPVVITSGCASQSLVKVLRGEKIGTLFHKNASLWEPSKETSVREMAVAARDCSRR
LQNLSSEERKKILLDVADALEANEDLIRSENEADLAAAHEAGYESALVSRLTLKPGKIASLAKSVRTLANMEDPINE
ILKRTEVADGLVLEKTSCPLGVLLIIFESRPDALVQIASLAIRSGNGLLLKGGKEAMRSNAILHKVITNAIPDNVGE
KLIGLITTRDEIADLLKHDDVIDLVIPRGSNKLVAQIKSSTKIPVLGHADGVCHVYIDKSADMDMAKRIVMDAKIDY
PAACNAMETLLVHKDLMKTPELNDILVALKTAGVNLYCGPVAHKVLGYPKADSLHLEYSSMACTVEIVDDVQSAIDH
IHRYGSAHTDCVVTTDDKVAETFLRQVDSAAVLYNASTRFSDGARFGLGAEVGISTGRIHARGPVGVEGLLTTRWLL
RGKGQVVNGDKDVEYTHKSLPLQ
>TraesCS1B02G290600.1
MTAKVQAAVWASTGGVPVVITSGCASQSLVKVLRGEKIGTLFHKNASLWEPSKETSVREMAVAARDCSRRLQNLSSE
ERKKILLDVADALEANEDLIRSENEADLAAAHEAGYESALVSRLTLKPGKIASLAKSVRTLANMEDPINEILKRTEV
ADGLVLEKTSCPLGVLLIIFESRPDALVQIASLAIRSGNGLLLKGGKEAMRSNAILHKVITNAIPDNVGEKLIGLIT
TRDEIADLLKHDDVIDLVIPRGSNKLVAQIKSSTKIPVLGHADGVCHVYIDKSADMDMAKRIVMDAKIDYPAACNAM
TLLVHKDLMKTPELNDILVALKTAGVNLYCGPVAHKVLGYPKADSLHLEYSSMACTVEIVDDVQSAIDHIHRYGSAH
TDCVVTTDDKVAETFLRQVDSAAVLYNASTRFSDGARFGLGAEVGISTGRIHARGPVGVEGLLTTRWLLRGKGQVVN
GDKDVEYTHKSLPLQ
>TraesCS1D02G280700.2
MAGADLNRSFIKDVKRIIIKVGTAVITRNDGRLALGRIGALCEQVKDLNAQGYEVIMVTSGAVGVGRQRLRYRKLVN
SSFADLQKPQMELDGKACAAVGQSGLMALYDMLFTQLDVSSSQLLVTDSDFDNSNFRERLRETVESLLELRVIPIFN
ENDAISTRKAPYEDSSGIFWDNDSLAGLLALELKADLLVLLSDVDGLYSGPPSEPSSKLIHTYIKEKHYHEITFGDK
SRVGRGGMTAKVQAAVWASTGGVPVVITSGCASQSLVKVLRGEKIGTLFHKNASLWEPSKDTSVREMAVAARDCSRR
LQNLTSEERKKILVDVADALEANEDLIRSENEADLAAAHEAGYESALVSRLTLKPGKIASLAKSVRTLANMEDPINE
ILKRTEVADGLVLEKTSCPLGVLLIIFESRPDALVQIASLAIRSGNGLLLKGGKEAMRSNAILHKVITNAIPNNVGE
KLIGLITTRDEIADLLKHDDVIDLVIPRGSNKLVAQIKSSTKIPVLGHADGVCHVYIDKSADMDMAKRIVMDAKIDY
PAACNAMETLLVHKDLMKTPELNDILVALKTAGVNLYCGPVAHKVLGYPKADSLHLEYSSMACTVEIVDDVQSAIDH
IHRYGSAHTDCVVTTDDKVAETFLRQVDSAAVLYNASTRFSDGARFGLGAEVGISTGRIHARGPVGVEGLLTTRWLL
RGKGQVVNGDKDVEYTHKSLPLQ
>TraesCS1D02G280700.1
MTAKVQAAVWASTGGVPVVITSGCASQSLVKVLRGEKIGTLFHKNASLWEPSKDTSVREMAVAARDCSRRLQNLTSE
ERKKILVDVADALEANEDLIRSENEADLAAAHEAGYESALVSRLTLKPGKIASLAKSVRTLANMEDPINEILKRTEV

ADGLVLEKTSCPLGVLLIIFESRPDALVQIASLAIRSGNGLLLKGGKEAMRSNAILHKVITNAIPNNVGEKLIGLIT
TRDEIADLLKHDDVIDLVIPRGSNKLVAQIKSSTKIPVLGHADGVCHVYIDKSADMDMAKRIVMDAKIDYPAACNAM
TLLVHKDLMKTPELNDILVALKTAGVNLYCGPVAHKVLGYPKADSLHLEYSSMACTVEIVDDVQSAIDHIHRYGSAH
TDCVVTTDDKVAETFLRQVDSAAVLYNASTRFSDGARFGLGAEVGISTGRIHARGPVGVEGLLTTRWLLRGKGQVVN
GDKDVEYTHKSLPLQ
>TraesCS3A02G363700.2
MASFFLLLLPSRYETNAAAVAAVLLGLFAAVPFVSPPLLLRRDPGPSAAGTGDRRKRRGWHGRKGSDLVGTAVVTGQ
NGRLAMGRLGALCEQVKELNFQGYEVILVTSGAVGVGRQRLKYRKLINSSFADLQNPQLDLDGKACAAVGQSGLMAI
YDTLFSQLDVTSSQLLVTDRDFRDPSFGHQLRETVVSLLDLKVIPVFNENDAISTRRAPYEDSSGIFWDNDSLATLL
AKELDADLLIMLSDVEGLYSGPPSDPQSKIIHTYINEKHGKLINFGEKSRVGRGGMQAKVAAAVTAASKGVPAVIAS
GFVTDSIIKIMRGEKIGTLFHNEANVWDCSKEVTTREMAVAAKDCSRHLQNLSSEERKKILLDIAGALDANVDLIIS
ENEADLAAAQDSGYEKSLVARMTLKAGKITSLAESIRAIADMEDPISHTLKKTEVAKDLVFEKMYCPLGVLLIIFES
RPDALVQIAALAIRSGNGLLLKGGKEAMRSNTILHKVITSVIPDVVGKKLIGLVKSKDEIADLLKLDDVIDLVIPRG
SNRLVSQIKAQTKIPVLGHADGICHVYIDKSADMDMAKRIVLDAKVDYPAACNAMETLLVHKDLNKTEGLDDLLMEL
AKEGVVIYGGPVAHDTLKVPKVDSFHHEYSSMACTLEFVDDVQSAIDHINRYGSAHTDCIITTDKKSADTFLQQVDS
AAVFHNASTRFCDGTRFGLGAEVGISTGRIHARGPVGVDGLLTTRCILRGSGQVVNGDKGVVYTHKDLPLQ
>TraesCS3A02G363700.3
MGRLGALCEQVKELNFQGYEVILVTSGAVGVGRQRLKYRKLINSSFADLQNPQLDLDGKACAAVGQSGLMAIYDTLF
SQLDVTSSQLLVTDRDFRDPSFGHQLRETVVSLLDLKVIPVFNENDAISTRRAPYEDSSGIFWDNDSLATLLAKELD
ADLLIMLSDVEGLYSGPPSDPQSKIIHTYINEKHGKLINFGEKSRVGRGGMQAKVAAAVTAASKGVPAVIASGFVTD
SIIKIMRGEKIGTLFHNEANVWDCSKEVTTREMAVAAKDCSRHLQNLSSEERKKILLDIAGALDANVDLIISENEAD
LAAAQDSGYEKSLVARMTLKAGKITSLAESIRAIADMEDPISHTLKKTEVAKDLVFEKMYCPLGVLLIIFESRPDAL
VQHIIVQIAALAIRSGNGLLLKGGKEAMRSNTILHKVITSVIPDVVGKKLIGLVKSKDEIADLLKLDDVIDLVIPRG
SNRLVSQIKAQTKIPVLGHADGICHVYIDKSADMDMAKRIVLDAKVDYPAACNAMETLLVHKDLNKTEGLDDLLMEL
AKEGVVIYGGPVAHDTLKVPKVDSFHHEYSSMACTLEFVDDVQSAIDHINRYGSAHTDCIITTDKKSADTFLQQVDS
AAVFHNASTRFCDGTRFGLGAEVGISTGRIHARGPVGVDGLLTTRCILRGSGQVVNGDKGVVYTHKDLPLQ
>TraesCS3A02G363700.1
MGRGGIGGAVAAADLENSDSTRGFVRDVKRIVVKVGTAVVTGQNGRLAMGRLGALCEQVKELNFQGYEVILVTSGAV
GVGRQRLKYRKLINSSFADLQNPQLDLDGKACAAVGQSGLMAIYDTLFSQLDVTSSQLLVTDRDFRDPSFGHQLRET
VVSLLDLKVIPVFNENDAISTRRAPYEDSSGIFWDNDSLATLLAKELDADLLIMLSDVEGLYSGPPSDPQSKIIHTY
INEKHGKLINFGEKSRVGRGGMQAKVAAAVTAASKGVPAVIASGFVTDSIIKIMRGEKIGTLFHNEANVWDCSKEVT
TREMAVAAKDCSRHLQNLSSEERKKILLDIAGALDANVDLIISENEADLAAAQDSGYEKSLVARMTLKAGKITSLAE
SIRAIADMEDPISHTLKKTEVAKDLVFEKMYCPLGVLLIIFESRPDALVQIAALAIRSGNGLLLKGGKEAMRSNTIL
HKVITSVIPDVVGKKLIGLVKSKDEIADLLKLDDVIDLVIPRGSNRLVSQIKAQTKIPVLGHADGICHVYIDKSADM
DMAKRIVLDAKVDYPAACNAMETLLVHKDLNKTEGLDDLLMELAKEGVVIYGGPVAHDTLKVPKVDSFHHEYSSMAC
TLEFVDDVQSAIDHINRYGSAHTDCIITTDKKSADTFLQQVDSAAVFHNASTRFCDGTRFGLGAEVGISTGRIHARG
PVGVDGLLTTRCILRGSGQVVNGDKGVVYTHKDLPLQ
>TraesCS3D02G357200.2
MGRLGALCEQVKELNFQGYEVILVTSGAVGVGRQRLKYRKLINSSFADLQNPQLDLDGKACAAVGQSGLMAIYDTLF
SQLDVTSSQLLVTDRDFRDPSFGHQLRETVVSLLDLKVIPVFNENDAISTRRAPYEDSSGIFWDNDSLATLLAKELD
ADLLIMLSDVEGLYSGPPSDPQSKIIHTYINEKHGKLINFGEKSRVGRGGMQAKVAAAVTAASKGVPAVIASGFVTD
SIIKIMRGEKIGTLFHNEANVWDCSKEVTTREMAVAAKDCSRHLQNLSSEERKKILLDIAGALDANVDLIISENEAD
LAAAQDSGYEKSLVARMTLKAGKITSLAESIRAIADMEDPISHTLKKTEVAKDLVFEKMYCPLGVLLIIFESRPDAL
VQHIIVQIAALAIRSGNGLLLKGGKEAMRSNTILHKVITSVIPDAVGKKLIGLVKSKDEIADLLKLDDVIDLVIPRG
SNRLVSQIKAQTKIPVLGHADGICHVYIDKSADMDMAKRIVLDAKVDYPAACNAMETLLVHKDLNKTEGLDDLLMEL
AKEGVVIYGGPVAHDTLKVPKVDSFHHEYSSMACTLEFVDDVQSAIDHINRYGSAHTDCIITTDKKSADTFLQQVDS
AAVFHNASTRFCDGTRFGLGAEVGISTGRIHARGPVGVDGLLTTRCILRGSGQVVNGDKGVVYTHKDLPLQ
>TraesCS3D02G357200.1
MGRGGIGGAVAAADLENSDSTRGFVRDVKRIVVKVGTAVVTGQNGRLAMGRLGALCEQVKELNFQGYEVILVTSGAV
GVGRQRLKYRKLINSSFADLQNPQLDLDGKACAAVGQSGLMAIYDTLFSQLDVTSSQLLVTDRDFRDPSFGHQLRET
VVSLLDLKVIPVFNENDAISTRRAPYEDSSGIFWDNDSLATLLAKELDADLLIMLSDVEGLYSGPPSDPQSKIIHTY
INEKHGKLINFGEKSRVGRGGMQAKVAAAVTAASKGVPAVIASGFVTDSIIKIMRGEKIGTLFHNEANVWDCSKEVT
TREMAVAAKDCSRHLQNLSSEERKKILLDIAGALDANVDLIISENEADLAAAQDSGYEKSLVARMTLKAGKITSLAE
SIRAIADMEDPISHTLKKTEVAKDLVFEKMYCPLGVLLIIFESRPDALVQIAALAIRSGNGLLLKGGKEAMRSNTIL
HKVITSVIPDAVGKKLIGLVKSKDEIADLLKLDDVIDLVIPRGSNRLVSQIKAQTKIPVLGHADGICHVYIDKSADM
DMAKRIVLDAKVDYPAACNAMETLLVHKDLNKTEGLDDLLMELAKEGVVIYGGPVAHDTLKVPKVDSFHHEYSSMAC

TLEFVDDVQSAIDHINRYGSAHTDCIITTDKKSADTFLQQVDSAAVFHNASTRFCDGTRFGLGAEVGISTGRIHARG
PVGVDGLLTTRCILRGSGQVVNGDKGVVYTHKDLPLQ
>TraesCS3B02G395900.1
MGRGGIGGAVAAADLENSDSTRGFVRDVKRIVVKVGTAVVTGQNGRLAMGRLGALCEQVKELNFQGYEVILVTSGAV
GVGRQRLKYRKLINSSFADLQNPQLDLDGKACAAVGQSGLMAIYDTLFSQLDVTSSQLLVTDRDFRDPSFGHQLRET
VVSLLDLKVIPVFNENDAISTRRAPYEDSSGIFWDNDSLATLLAKELDADLLIMLSDVEGLYSGPPSDPQSKIIHTY
INEKHGKLINFGEKSRVGRGGMQAKVAAAVTAASKGVPAVIASGFVTDSIIKIMRGEKIGTLFHNEANVWDCSKEVT
TREMAVAAKDCSRHLQNLSSEERKKILLDIAGALDANVDLIISENEADLAAAQDSGYEKSLVARMTLKAGKITSLAE
SIRAIADMEDPISHTLKKTEVAKDLVFEKMYCPLGVLLIIFESRPDALVQIAALAIRSGNGLLLKGGKEAMRSNTIL
HKVITSVIPDAVGKKLIGLVKSKDEIADLLKLDDVIDLVIPRGSNRLVSQIKAQTKIPVLGHADGICHVYIDKSADM
DMAKRIVLDAKVDYPAACNAMETLLVHKDLNKTEGLDDLLMELAKEGVVIYGGPVAHDTLKVPKVDSFHHEYSSMAC
TLEFVDDVQSAIDHINRYGSAHTDCIITTDKKSADTFLQQVDSAAVFHNASTRFCDGTRFGLGAEVGISTGRIHARG
PVGVDGLLTTRCILRGSGQVVNGDKGVVYTHKDLPLQ

PDH:
>TraesCS1A02G209100.2
MLCLCVQASVCIKITALCPIALLEKTSDLLRWQHKNPSVHLPWKQHAFPILSDSSPLYLTPSEPAALTAEEERELQL
AHDRLLAVGARCAEHGIPLLVDAEYASVQPSIDYFTFVGALACNGGGRPIVHGTVQAYLRDARDRLEAMVRAAEEER
VCLGVKIVRGAYLTREARLAESLGVPSPIHGSIQDTHDCYNGCAAFLLERVRRGSASVMLATHNVESGQLAAARAQE
LGIGKGDRNLQFAQLMGMADGLSLGLRNAGFQEGAG
>TraesCS1A02G209100.1
MRPRPGGRRGRRGRRRALLRMGGILDYGIEDAEDGAACDRNVAGFLAAVDVAAKLPPGSASVCIKITALCPIALLEK
TSDLLRWQHKNPSVHLPWKQHAFPILSDSSPLYLTPSEPAALTAEEERELQLAHDRLLAVGARCAEHGIPLLVDAEY
ASVQPSIDYFTFVGALACNGGGRPIVHGTVQAYLRDARDRLEAMVRAAEEERVCLGVKIVRGAYLTREARLAESLGV
PSPIHGSIQDTHDCYNGCAAFLLERVRRGSASVMLATHNVESGQLAAARAQELGIGKGDRNLQFAQLMGMADGLSLG
LRNAGFQVSKYLPYGPVEHIIPYLIRRAEENRGLLSASAFDRQLLRKELVRR
FKNAVMGRE
>TraesCS1D02G212400.1
MAIASRITKRALSTFAAAAKLPEAAVAAAGAEAVPAPSSAQHQQQQQVLEFEDTERLFTGEPSTALVRTLAALQLMS
AGPLVDVGLAALRSPAVAASPVVQAAARATAYKHFCAGETADEAAARVQRLWRGGMGGILDYGIEDAEDGAACDRNV
AGFLAAVDVAAALPPGSASVCIKITALCPIALLEKTSDLLRWQHKNPSVHLPWKQHAFPILSDSSPLYLTPSEPAAL
TAEEEHELQLAHDRLLAVGARCAEHDIPLLVDAEYASVQPSIDYFTFVGALACNGGGRPIVHGTVQAYLRDARDRLE
AMVRAAEEERVCLGVKIVRGAYLTREARLAESLGVPSPIHGSIQDTHDCYNGCAAFLLERVRRGSASVMLATHNVES
GQLAAARAQELGIGKGDRNLQFAQLMGMADGLSLGLRNAGFQVSKYLPYGPVEHIIPYLIRRAEENRGLLSASAFDR
QLLRKELVRRFKNAVMGRE
>TraesCS1B02G223300.1
MAIASRITKRALSTFAAAAKLPEAAVAAAGAEAVPVPSSAQQQQHQVLEFEDTGRLFTGEPSTALVRTLAALQLMSA
GPLVDVGLAALRSPAVAASPVVQAAARATAYKHFCAGETADEAAARVQRLWRGGMGGILDYGIEDAEDGAACDRNVA
GFLAAVDVAAALPPGSASVCIKITALCPIALLEKTSDLLRWQHKNPSVHLPWKQHAFPILSDSSPLYLTPSEPAALT
AEEERELQLAHDRLLAVGARCAEHDIPLLVDAEYASVQPSIDYFTFVGALACNGGGRPIVHGTVQAYLRDARDRLEA
MVRAAEEERVCLGVKIVRGAYLTREARLAESLGVPSPIHGSIQDTHDCYNGCAAFLLERVRRGSASVMLATHNVESG
QLAAARAQELGIGKGDRNLQFAQLMGMADGLSLGLRNAGFQVSKYLPYGPVEHIIPYLIRRAEENRGLLSASAFDRQ
LLRKELVRRFKNAVMGRE

OAT:
>TraesCS5D02G384500.2
MAAAVARRGAARALALAMARRGMCSASVPAAERAAAALSSEELIRMERDCSAHNYHPIPMVFSKGEGSHILDPEGNK
YIDFLSAYSAVNQGHCHPKVLRALIEQAERLTLSSRAFYNDKFPVFAEYLTSMFGYDMMLPMNTGAEGVETAIKLAR
KWGYEKKNIPKNEALIVSCCGCFHGRTLGVISMSCDNDATRGFGPLVPGHLKVDFGDIDGLEKIFKEHGDRICGFLF
EPIQGEAGVIIPPDGYLKAVRDLCSRHNILMIDDEIQTGIARTGKMLACDWEDVRPDMVILGKALGAGVVPVSAVLA
DKDIMLCIKPGEHGSTFGGNPLASAVAVASLKVVKDEGLVERAAELGQEFRDQLQKVQQKFPHIIREIRGRGLLNAV
DLCSKALYPASAYDICIKLKERGILAKPTHDTIIRSEELAEASKALSDVLEHDLPQLQKQIKKPESEAKTPVCDRCG
RDL
>TraesCS5D02G384500.1
MAAAVARRGAARALALAMARRGMCSASVPAAERAAAALSSEELIRMERDCSAHNYHPIPMVFSKGEGSHILDPEGNK
YIDFLSAYSAVNQGHCHPKVLRALIEQAERLTLSSRAFYNDKFPVFAEYLTSMFGYDMMLPMNTGAEGVETAIKLAR
KWGYEKKNIPKNEALIVSCCGCFHGRTLGVISMSCDNDATRGFGPLVPGHLKVDFGDIDGLEKIFKEHGDRICGFLF

EPIQGEAGVIIPPDGYLKAVRDLCSRHNILMIDDEIQTGIARTGKMLACDWEDVRPDMVILGKALGAGVVPVSAVLA
DKDIMLCIKPGEHGSTFGGNPLASAVAVASLKVVKDEGLVERAAELGQEFRDQLQKVQQKFPHIIREIRGRGLLNAV
DLCSKALYPASAYDICIKLKERGILAKPTHDTIIRLAPPLSISSEELAEASKALSDVLEHDLPQLQKQIKKPESEAK
TPVCDRCGRDL
>TraesCS5A02G375000.1
MAAAAMARRGVARRGVARALAIARRGMCSTSAPAAALSSEELIRMEQDCSAHNYHPIPMVFSKGEGSHILDPEGNKY
IDFLSAYSAVNQGHCHPKVLRALIEQAERLTLSSRAFYNDKFPVFAQYLTSMFGYDMMLPMNTGAEGVETAIKLARK
WGYEKKNIPKNEALIVSCCGCFHGRTLGVISMSCDNDATRGFGPLVPGHLKVDFGDIDGLEKIFKEHGDRICGFLFE
PIQGEAGVIIPPDGYLKAVRDLCSRHNILMIDDEIQTGIARTGKMLACDWEGVRPDMVILGKALGAGVVPVSAVLAD
KDIMLCIKPGEHGSTFGGNPLASAVAIASLKVVKDEGLVERAAELGQEFRDQLRKVQQKFPDIIREIRGRGLLNAVD
LSSKALYPASAYDICIKLKERGILAKPTHDTIIRLAPPISISPEELTEASKALSDVLEHDLPQLQKQIKKPESEAKT
PVCDRCGRDL
>TraesCS5B02G376900.1
MAAIISRRGAARALALAMARRGMCSAPAPAAALSSEELIRMEQDCSAHNYHPIPMVFSKGEGSHILDPEGNKYIDFL
SAYSAVNQGHCHPKVLRALIEQAERLTLSSRAFYNDKFPVFAQYLTSMFGYDMMLPMNTGAEGVETAIKLARKWGYE
KKNIPKNEALIVSCCGCFHGRTLGVISMSCDNDATRGFGPLVPGHLKVDFGDIDGLEKIFKEHGDRICGFLFEPIQG
EAGVIIPPDGYLKAVRDLCSRHNILMIDDEIQTGIARTGKMLACDWEDVRPDMVILGKALGAGVVPVSAVLADKDIM
LCIKPGEHGSTFGGNPLASAVAIASLKVVKDEGLVERAAELGQEFRDQLQKVQQKFPRIIREIRGRGLLNAVDLSSK
ALYPASAYDICIKLKERGILAKPTHDTIIRLAPPISISPEELAEASKALSDVLEHDLPQLQKQIKKPDSEAKIPVCD
RCGRDL

Sorghum bicolor
P5CR:
>SORBI_3003G424300
MAAPPVQPVPPAAAAAVNGDAFRLGFVGAGNLAESIARGVAASGVLPASAIRTAPHRRPERGEAFASFGACLLQTNA
QVVDDSDVIVISVKPQIVKQVLLELRPRLSEEKLLVSIAAGIKMQDLQDWSGQRRIIRVMPNTPSAVGQAASVMCLG
EMATQDDENRVRKLFSAIGKVWTAEEKYFDAVTGLSGSGPAYIFLAIEAMADGGVAAGLPRDLALGLASQTVLGAAT
MVSQTGKHPGQLKDQVTSPAGTTIAGIQELEKGAFRGTLISAVVAAAKRCRELS
>SORBI_3008G013300
MDPSAEPPAAAWPPWTSLLLRALSRRRTWVVLFLAVYAALLSSSWSLLASVRAWYYSSASSTSSSAPAWPAALYASV
MYGAVFGLLSMGAALAVAAPAMLVTWTTVLVLLAFAGRPPRSLVAEGRRATRDIAGLALRVLLREGNAVAALCAAAS
FVALLLGRRDVEDESATTLPNQ

P5CDH:
>SORBI_3009G212600
MSRLLSRRQIDAVRRSAPLACVSRWLHTPSFATVSPHEVSGSSPAEVQNFVQGSWTASANWNWIVDPLNGDQFIKVA
EVQGTEIKPFVESLSKCPKHGLHNPLKAPERYLMYGDISAKAAHMLGQPAVSDFFAKLIQRVSPKSYQQALAEVQVS
QKFLENFCGDQVRFLARSFAVPGNHLGQRSNGYRWPYGPVAIITPFNFPLEIPLLQVMGALYMGNKPVLKVDSKVSI
VMEQMIRLLHDCGLPAEDMDFINSDGVTMNKLLLEANPKMTLFTGSSRVAEKLAADLKGRVKLEDAGFDWKILGPDV
QEVDYVAWVCDQDAYACSGQKCSAQSVLFMHKNWSSSGLLEKMKKLSERRKLEDLTIGPVLTVTTEAMIEHMNNLLK
IQGSKVLFGGEPLANHSIPKIYGAMKPTAVFVPLEEILKSGNFELVTKEIFGPFQVVTEYSEDQLELVLEACERMNA
HLTAAVVSNDPLFLQDVLGRSVNGTTYAGIRARTTGAPQNHWFGPAGDPRGAGIGTPEAIKLVWSCHREIIYDVGPV
PKSWALPSAT
>SORBI_3009G160100
MATADPTRTFMKDVKRVIIKLGTAVVTRHDGRLALGRLGALCEQVKELNALGYEVIIVTSGAVGVGKQRLKYRKLVN
SSFADLQKPQMELDGKACAAVGQSGLMALYDMLFTQLDVSSSQLLVTDSDFENPNFRERLRETVESLLDLKVVPIFN
ENDAISTRKAPYEDSSGIFWDNDSLAGLLAIELKADLLVLLSDVDGLYSGPPSEPQSKIIHTYIKEKHHNEITFGDK
SRVGRGGMTAKVKAAFVASNSGTPVVITSGFASQSIVRVLQGEKIGTLFHKDASLWEPSKDVSAREMALGARESSRR
LQNLSSDERKKILLDVADALEENVDLIRTENEIDVSAAQEAGYEPSLVARLTLKPGKIASLAKSIRTLAYMEDPINQ
IIKRTEVAEDLVLEKTSCPLGVLLIVFESRPDALVQIASLAIRSGNGLLLKGGKEAMRSNTILHKVITSAIPSNVGE
KLIGLVTSRDEIADLLKLDDVIDLVIPRGSNKLVSQIKSSTKIPVLGHADGICHVYIDKSADMNMAKRIVLDAKIDY
PAACNAMETLLVHKDLINAPGLDDLLLALKTEGVAIYGGPVAHELLCIPKADSLHHEYSSMACTIEFVDDVQSAIDH
IHRYGSAHTDCIVTTDDKVAETFLRQVDSAAVFYNASTRFSDGARFGLGAEVGISTGRIHARGPVGVEGLLTTRWIM
RGSGQVVNGDKDIAYTHKNLPLQ

P5CS:
>SORBI_3003G356000

MGRGGIGGAVGMAMENADSARAFVKDVKRIIIKVGTAVVTGQNGRLAMGRLGSLCEQVKQLNFQGYEVILVTSGAVG
VGRQRLQYRKLIHSSFADLQNPQMNFDGKACAAVGQSGLMAIYDTLFSQLDVTSSQLLVTDRDFKDPSFGDQLRETV
FALLNLKVIPLFNENDAISTRRQSDEDSSGVFWDNDSLAALLAAELNADLLIMLSDVEGLYSGPPSDPQSKIIHTYV
NEKHGKLISFGEKSNVGRGGMQAKVAAAANAASKGVPVVIASGFATDSIIKVLKGEKIGTLFHNEANLWECSKEATA
REMAVAARDCSRRLQKLSSDERKKILLDIADALEANEGAIRSENEADVEAAQGAGYEKSLVARMTLKPGKITNLARS
IRAIADMEDPISHTLKRTEVAKDLVFEKAYCPLGVLLIIFESRPDALVQIASLAIRSGNGLLLKGGKEAMRSNAILH
KVITGVIPDIVGKKLIGHVTSKDEIADLLALDDVIDLVIPRGSKNLVSQIKATTKIPVLGHADGICHVYIDKSADMD
MAKRIVLDAKVDYPAACNAMVYL

OAT:
>SORBI_3001G156100
MVFSKGEGSHIVDPEGNKYIDFLSAYSAVNQGHCHPKVLRALIEQAERLTLSSRAFYNDKFPIFAEYLTSMFGYDMM
LPMNTGAEGVETAIKLARKWGYEKKRIPKNEALLVSCCGCFHGRTLGVISMSCDNDATRGFGPLVPGHLKVDFGDID
GLKKIFEEHGDRICGFLFEPIQGEAGVVIPPDGYLKGVRDLCSKHNILMIADEIQTGIARTGKMLACDWENIRPDMV
ILGKALGAGVVPVSAVLADKDVMLCIRPGEHGSTFGGNPLASAVAVASLKVVRDEGLVERAAKLGQEFRDQLQKVQQ
KFPQIIREVRGRGLLNAVDLNNDALSPASAYDICIKLKERGILAKPTHDTIIRLAPPLSISPEELAEASKALSDVLE
HDLPQLQKQIKKPESEAEKPVCDRCGRDLYG

PDH:
>SORBI_3001G304700
MAIASRVTKRALSTFAAAKLPEEAVAAAAAGEAIAATTAAAVPLASSERTAIARAALLQFEDTGRLFAGEPTSALLR
TLAALQALSVGPLVDAATAALRSPAVAGSALGRAAARATAYRHFCAGETADEAAAVVRRLWRGGMGGILDYGIEDAE
DGDACDRNAAGFISAVDVAAALPPGSASVCIKITALCPIALLEKTSDLLRWQKKHPSFNLPWKTHSFPILSDSSPLH
LTPSEPPALTSEEETELQLAHERLLAVCARCAEHGIPLLVDAEYATVQPAIDYFTFVGALAFNDGAGAGDCEQRPIV
HGTIQAYLRDARDRLEAMVRSAERERVRLGLKVVRGAYLARETRLAATLGVPSPIHGSIQETHDCYNGCAGFLLDRV
RRGTASVMLATHNVESGKLAAARAQELGIPRGDRNLQFAQLMGMADGLSLSLRNAGFQVSKYLPYGPVEQIIPYLIR
RAEENRGLLSASSFDRHLLRYVLYGRPITPSGMLQLMV

Cucumis sativus
P5CR:
>Csa_4G354630
MEVLPISIDAYRLGFIGAGKMAESIAKGIVQSGLLPPSRISTAVHSNPSRRIAFESFGVRVLPKNDNVVEESDVVIL
SVKPQVVKNVVLKLRPLLSGKKLLVSVAAGVKLKDLQDWAGHNRFIRVMPNTPAAVGEAASVMSLGEGAIKEDGQLV
AKLFGSVGKIWEAEEKYFDAVTGLSGSGPAYIFLAIEALADGGVAAGLPRELAMGLASQTVLGAASMVTKTGKHPGQ
LKDDVTSPGGTTIAGIHELEKGGLRGMFMNAVVAAAKRGQELSPK
>Csa_6G127980
MNPPQIIQLIIRTTPPPHKIRKESSSSSVEMAVLYKTSQKEERTKEPSWSSPKQWPLVLLLLGCTFGSILFGSVHQN
SSASVVVVGGLMVLAAVGVAVAVAVIGVAGLVTWITVVVFLWLIGRSRRRLVAEGRKISKEVMVGFMVRVLLKEGNV
LGAISAAVFGYLGLCMGSIIISSYSKLFWLK
>Csa_7G048040
MADNKEEEPETAPPQQAIPKSYSLFMKVMSKRRTWVCLFVLVYALLLSSSWNFLVSILSWYKSQVEASSSSFGWPAI
YASLLLGGVFGVISMVAALAVMIPATLVTWIAIVVLLYFFGKPRRLLVVEGRKITKEIFGVVVKILLKEGNLVAAVC
AVLGYFALFRKTNES
>Csa_1G003480
MATATSAIFVLLSGLCFLVFNSDNLMGRWLPILGLLAVAVVVLMAARATMVAWITVLVLLAFAGNRRRVLVKDGRKI
TADVAMYLASVIVKERGLLAVAFAALFSFLAVLRSTEIDLLSSFSA
>Csa_1G003500
MLTPTTATATSAIFVLLSGLCFLVFNSDNLMGRWLPILGLLAVAVVALMAARATMVAWITVLVLLAFAGNRRRVLVK
DGRKITADVAMYLASVIVKERGLLAVAFAALFSFLAVLRSTEIYLLSSFSA

P5CDH:
>Csa_1G045490
MAPVLSRLLLRRNVHTPFLRTSTTAFNFFRTIHSTVFSTVEVDQISGSKPGDVLNLVQGKWIGSSGWNTIVDPLNGE
PFIRVAEVNETEIQPFVKSLTKCPKHGLHNPFKSPERYLLFGDVSSKSADVLSKPEVTDFFARLIQRVSPKSYQQAC
AEVNVTVKFLRNFSGDQVRFLARSFAVPGDHLGQQSHGFRWPYGPVAIITPFNFPLEIPVLQLMGALYMGNKPVLKV
DSKVSIVMEQMIRLLHHCGLPLEDLDFINCDGKTMNKFLMEANPSMTLFTGSSRVADKLAVDLKGRIKLEDAGFDWK
VLGPDVREEDYVAWVCDQDAYACSGQKCSAQSILFMHENWSTTSLISKIKDLAERRNLTDLTIGPVLTLTTEAILDH

LNKLIKIPGAKLLFGGEPLKNHSIPPVYGAIKPTALYIPLEEMMKDENYELVTKEIFGPFQIVTEYKRDQLSVVLDA
LERMHAHLTAAVVSNDPLFLQEVIGNTVNGTTYVGLRARTTGAPQNHWFGPAGDPRGAGIGTPEAIKLVWSCHREII
YDIGPLPSHWTVPSST
>Csa_3G733920
MDPSRVFTKNARRIVVKVGTAVVTRGDGRLALGRLGALCEQLKELNSRGYEVILVTSGAVGLGRQRLRYRRLANSSF
ADLQNPQGDFDGKACAAVGQSSLMALYDTMFSQLDVTSSQLLVTDSVFSDTGFRQQLSETVNALLDLRVIPIFNEND
AVSTRKTPYEDSSGIFWDNDSLAGLLALELKADLLVLLSDVEGLYSGPPSDPNSKLIQTFVKEKHLGQITFGAKSRV
GRGGMTAKVNAAVYASCAGIPVVITSGFATDNIIKVLQGEMKGTLFHKDAHLWTLVKEVSAREMAISARESSRRLQA
LKSEDRRKILMDVADALEDNENMILAENSADIKAAEEAGYEKPLISRLALKPGKVKLLANSVRKLADMEEPIGRILK
RSELARDLVLESVSCSLGVLLVVFESRPDALVQIASLAIRSGNGLLLKGGKEARRSNAALHKVITSAIPDTVGEKLI
GLVTSREEIPDLLKLDNVIDLVIPRGSNKLVSQIKESTKIPVLGHSDGICHVYVDKSADIEMAKRIVSDAKVDYPAA
CNAMETLLVHEDLANNGGLIKLITELRKEGVTLYGGKRASELLKINEAHSFHHEYNSLTCTVEIVDDVFAAIDHINE
HGSSHTDCIITEDQKVAEIFLNQVDSAAVFHNASTRFCDGARFGLGAEVGISTSRIHARGPVGVEGLLTTRWILRGT
GQVVNGDNGVAYIHKDLALKN

P5CS:
>Csa_6G008780
MDSMDRSRTFLRDVKRLVIKVGTAVVTRSDGRLALGRLGALCEQIKELNSQEYEVILVSSGAVGIGRQRLRYRKLVN
SSFADLQKPQVDLDGKACAAVGQNSLMALYDTLFSQLDVTSAQLLVTDNDFRDKDFRRQLSDTVKSLLTLKVVPIFN
ENDAVSTRKAPYEDSSGIFWDNDSLAALLALELKADLLVLLSDVDGLYSGPPSDPHSKLIHTYVKERHQGEITFGDK
SRVGRGGMTAKVKAATGAAYAGIPVVITSGYAPGNILKVLKGDRIGTLFHQDAHLWTPQKDVGARDMAVAARESSRR
LQAISSQERRKILLDIADALEANEKLISTENEADVAEAQQSGYEKALVSRLAMKPGKISSLANSIRTLANMEDPIGH
VLKRTEVSDGLVLEKTSSPLGVLLIVFESRPDALVQIASLAIRSGNGLLLKGGKEARRSNAILHKVITEAIPESIGG
TLIGLVTSREEIPDLLKLDDVIDLVIPRGSNKLVSQIKNSTKIPVLGHADGICHVYVDKSADLEMSKRIVLDAKIDY
PAACNAMETLLVHKDLVQTGGLNELVVNLRIEGVTLNGGPRASSLLNIPEARTFHHEYNSLACTLEIVDDVFDAIDH
IHQHGSAHTDCIVTEDHEVAEVFLRQVDSAAVFHNASTRFSDGARFGLGAEVGISTSRIHARGPVGVEGLLTTRWIL
RGSGQVVDGDKGVVYTHRDIKIES

PDH:
>Csa_5G643300
MAQAIVSTQSKISKRVFFYVFNRPLNTAASSPAAAFTAATATTSPPSPLHLNPPSTPTVDFTDSRALFGSIPTSDLL
HATATLHAAAVGPVVDVGMWVMNSKLMDVELFRDVVLGTVKHSFYRHFCAGEDDTSVAKTVRRLHDVGLRSMLDYAL
EYADDEASCDRNLDGFLSTVEATKSLPSGSASFVVTKVSAICPLRLLERVSDLLRWQQKNPSFNLPWKLQTLPIFSE
SSPLYHTLEQPEPLTREEEKSLQMSHERLMKICQSCVDANVPLAIDAEHTKVQPAIDYFTYSAAIIHNKDRNPIVYG
TIQAYLKDAKDRLLLANKEASKLKVPLGIKLVRGAYMSSESKLASSLGFESPIHDTIQDTHSCYNTCASFLLDDIAK
GSTGAILATHNVESGKLAASRAYEIGIGKLKQKLEFAQLYGMSEALSFGLRNAGFQVSKYMPFGPVDMVMPYLLRRA
EENRGLLSASNLDRELMRKELGRRMKEYIS

OAT:
>Csa_1G025890
MASRRQLQCLVGLVFRGRRPFSTVPESSASSFTVSEKLIDMEKDHSAHNYHPIPVVFSEAKGSSIWDPEGKRYLDFL
SAYSAVNQGHCHPKIVKAFQEQAEKLTLSSRAFYNDKFPHFADYLTQLFGYDMVLPMNTGAEGVETALKLARKWGYK
KKRIPKDEAFIVSCCGCFHGRTLGVISMSCDNEATREFGPLLPGHLKVDFGDADALEKIFKEHGDRIAGFLFEPIQG
EAGVIIPPDGYLKAVRDLCTKYNILMIADEIQSGLARSGKLLACDWENVRPDLVILGKALGGGVIPVSAVLADKEVM
LCIQPGEHGSTFGGNPLASAVAIASLEVIKDERLAERSAKLGEELKEQLIKIQERFPQYIKNVRGRGLFNAVELKGK
ALSPVSAYDICMKLKERGVLAKPTHDTIIRLTPPLSISLDELKEGSKAVHDVLEIDLPKLMKEKPHTASSSDSNICD
RCGRNLYASED
